# ZMAT1 Promotes Osteoclastogenesis Through TRIM46 Mediated YAP1 Degradation and Inhibits Osteoblastogenesis

**DOI:** 10.1002/advs.202521783

**Published:** 2026-03-02

**Authors:** Xinyu Chang, Yijin Hou, Likun Cui, Huiqi Yu, Rong Liu, Junhao Sui, Zhong Zheng, Lu Liu, Jie Chen, Mengchen Chen, Chen Ding, Shuogui Xu, Sheng Xu, Hao Zhang

**Affiliations:** ^1^ Department of Traumatic Orthopedics Changhai Hospital Second Military Medical University Shanghai China; ^2^ National Key Laboratory of Medical Immunology and Institute of Immunology Second Military Medical University Shanghai China

**Keywords:** transcription factor, TRIM46, ubiquitination, YAP1, Zmat1

## Abstract

Osteoclasts and osteoblasts play critical roles in bone remodeling, and their dysregulation leads to pathological bone loss. However, the precise mechanisms underlying the regulation of differentiation remain unclear. This study investigated the role of the transcriptional regulator Zinc Finger Matrin‐Type 1 (*Zmat1*) in both osteoclastogenesis and osteoblastogenesis. *Zmat1* deficiency resulted in decreased osteoclast activity, and bone resorption. Mechanistically, ZMAT1 was significantly upregulated during osteoclast differentiation and acted as a transcriptional repressor of the E3 ubiquitin ligase TRIM46, which regulates YAP1 degradation via K48‐linked ubiquitination. Furthermore, *Zmat1* deficiency enhanced osteoblast activity and bone formation. These findings highlight a novel ZMAT1/TRIM46/YAP1 axis, providing new insights into the transcriptional regulation of both osteoclast and osteoblast differentiation, and present potential therapeutic targets for osteoporosis.

## Graphical abstract

*Zmat1* deficiency mitigates pathological bone loss by impairing osteoclastogenesis and promoting osteoblastogenesis. Mechanistically, in osteoclasts, *Zmat1* loss relieves transcriptional repression of the E3 ligase TRIM46, promoting YAP1 degradation and inhibiting osteoclastogenic genes. These findings highlight the ZMAT1–TRIM46–YAP1 axis as a potential therapeutic target for restoring bone remodeling balance.

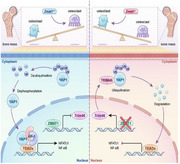

## 1 Introduction

Bone is a dynamic organ that continually remodels to maintain skeletal integrity and adapt to mechanical stress [1]. This remodeling process is meticulously regulated by the coordinated actions of osteoclasts, osteoblasts, and osteocytes [2, 3]. Osteoclasts, derived from myeloid lineage cells, are responsible for bone resorption, whereas osteoblasts are involved in bone formation [4, 5]. The equilibrium between these two processes is crucial for maintaining bone density and structural integrity. Disruption of this balance leads to pathological conditions such as osteoporosis, where increased osteoclast activity results in excessive bone resorption and subsequent bone loss [6, 7].

Emerging research has highlighted the functionality of certain transcription factors in the regulation of osteoblast and osteoclast activities, offering potential new avenues for therapeutic intervention. Transcription factors such as NFATc1, PU.1, and C‐FOS are crucial for osteoclast differentiation and function [8, 9, 10], while RUNX2, Osterix, and ATF4 are key regulators of osteoblast differentiation [11, 12, 13]. NFATc1 is essential for osteoclast differentiation by promoting the expression of osteoclast‐specific genes, whereas PU.1 and C‐FOS are involved in the early stages of osteoclast lineage commitment [14, 15]. Conversely, RUNX2 is indispensable for osteoblast differentiation and bone formation, and acts as a master regulator by promoting the expression of bone matrix proteins and osteoblast‐specific genes [16]. Osterix functions downstream of RUNX2 and is required for the maturation of pre‐osteoblast into fully functional osteoblast [17, 18]. ATF4 enhances osteoblast differentiation and function by regulating the expression of genes involved in amino acid transport and collagen synthesis [19, 20, 21]. SOX4 promotes bone resorption by inhibiting osteoblastogenesis and increasing osteoclastogenesis [22]. The intricate balance between these transcription factors ensures the proper regulation of bone remodeling, and disruptions in their expression or activity can lead to bone metabolic disorders such as osteoporosis.

Zinc finger matrin‐type 1 (*Zmat1*) is a transcription factor mapped to Xq22.1 and encodes a protein with three U1‐like zinc fingers of the C2H2‐type. While *Zmat1* and its family members (*Zmat1‐5*) are implicated in various cellular stress response pathways, including DNA damage, cell cycle arrest, and apoptosis, their roles in bone biology and osteoclast differentiation are largely unexplored [23, 24]. Considering that transcriptional control is central to osteoclast lineage commitment and activity, investigation of novel transcription factors such as ZMAT1 could provide new insights into the molecular mechanisms governing bone resorption. The role of ZMAT1 in the skeletal system, particularly in bone cell differentiation and remodeling—remains largely unexplored, making it a potentially critical regulator of bone metabolism and a promising target for further investigation.

The Hippo signaling pathway, a well‐conserved regulatory network, is essential for controlling organ size by modulating cell proliferation, differentiation, and apoptosis [25]. YAP1, a key effector of the Hippo signaling pathway, functions as a transcriptional co‐activator when dephosphorylated, promoting the expression of genes involved in cell survival and differentiation [26]. Activation of the Hippo signaling pathway leads to phosphorylation of YAP1 by LATS1/2 kinases, resulting in its cytoplasmic retention and inhibition [27, 28, 29]. YAP1 has been reported to play crucial roles in both osteoblast and osteoclast functions. YAP1 promotes osteoblast proliferation and differentiation by interacting with transcriptional co‐regulators and signaling pathways [30, 31, 32]. In osteoclasts, YAP1 influences the differentiation and resorption activity [33, 34]. Despite reports of YAP1's involvement in bone cell functions, the regulatory mechanisms governing YAP1 activity in osteoblasts and osteoclasts are not yet well defined. This study aimed to address this gap by exploring the role of ZMAT1 in the regulation YAP1 in the context of bone homeostasis.

In this study, we investigated the expression and functional impact of *Zmat1* in the bone tissues from *Zmat1^−/−^* mice and their littermates. We found that *Zmat1* expression increases during osteoclastogenesis and regulates osteoclast differentiation. Mechanistically, ZMAT1 acts as a transcriptional repressor of TRIM46, the E3 ubiquitin ligase. *Trim46* facilitates YAP1 degradation through K48‐linked ubiquitination, indicating that the ZMAT1/TRIM46/YAP1 axis regulates osteoclast differentiation. Furthermore, *Zmat1* deficiency protects mice from cranial bone erosion by enhancing osteoblast activity, highlighting its potential as a therapeutic target in osteoporosis. ZMAT1's dual regulatory function in osteoclasts and osteoblasts underscores its significance in bone homeostasis. Our study provides mechanistic insights into the role of ZMAT1 in bone metabolism and offers novel therapeutic avenues for the treatment of osteoporosis and related skeletal disorders.

## 2 Results

### 2.1 ZMAT1 is Upregulated in Osteoclastogenesis, and is Associated with Impaired Fracture Healing

**FIGURE 1:**
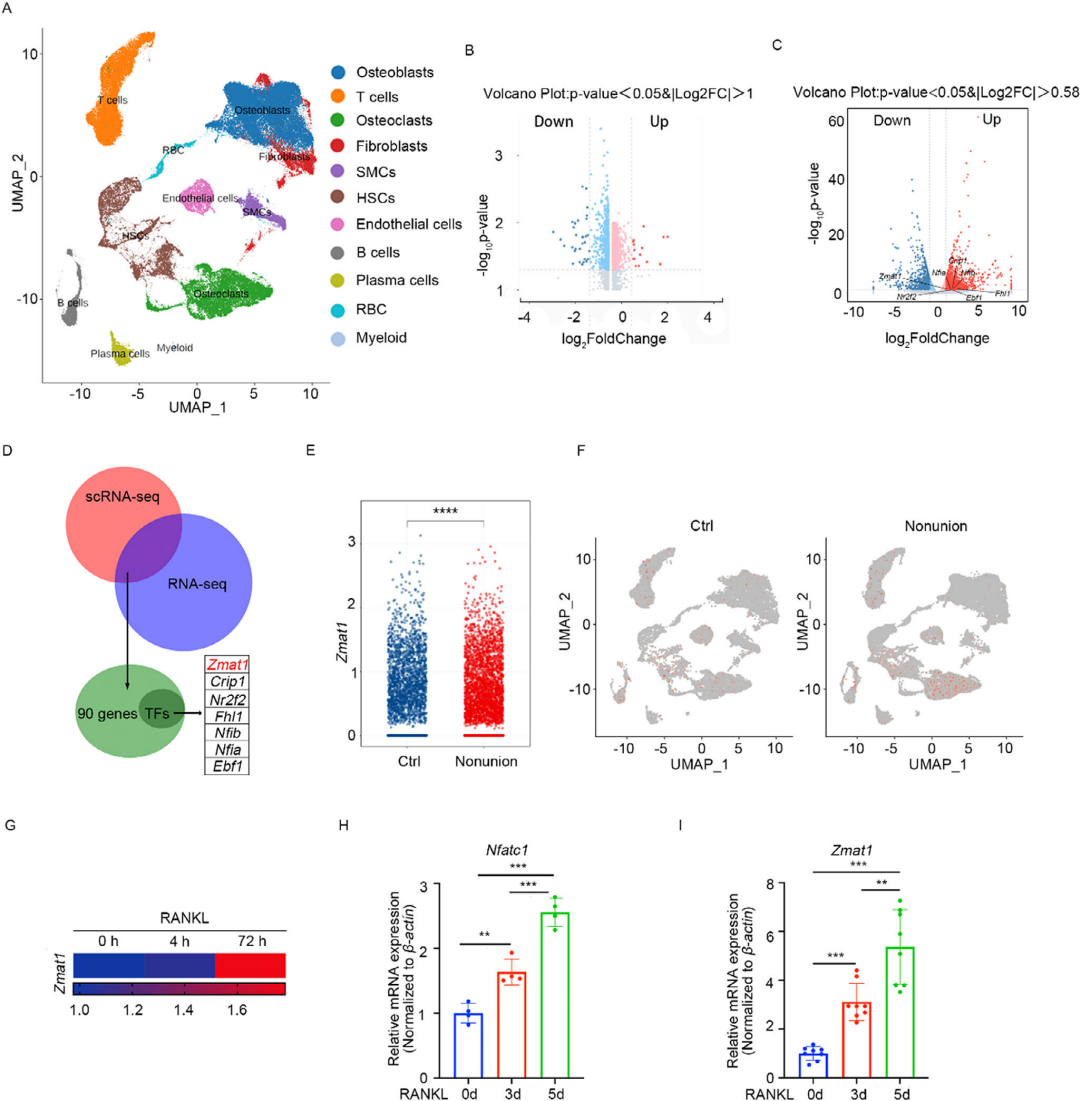
*Zmat1* is upregulated in osteoclastogenesis and correlates with fracture healing. (A) Uniform manifold approximation and projection visualization of 11 cell type clusters in the healing bone of patients after fracture. (B,C) Volcano plot showing DEGs in osteoblasts (B) and osteoclasts (C) from (A). (D) Venn analysis identified seven transcription factor genes. (E) Plot showing the relative expression of *Zmat1* in the nonunion bone of patients after fracture and in control cases. (F) Expression pattern of *Zmat1* in UMAP. (G) Heatmap of RNA‐seq data showing relative *Zmat1* expression in osteoclastogenesis from GSE274540. (H,I) Osteoclast marker gene *Nfatc1* (H) and transcription factor *Zmat1* (I) mRNA levels in BMDMs from wild‐type mice. All bar graphs are presented as the mean ± SD. ^**^ *p* < 0.01; ^***^ *p* < 0.001; n.s. not significant by one‐way ANOVA for multiple comparisons or two‐tailed Student's t‐test for paired comparisons.

To investigate the genes involved in osteoclastogenesis, we analyzed single‐cell RNA sequencing (scRNA‐seq) data from tissue samples collected from patients with non‐union and normal fracture healing (GSE217792). Clustering analysis revealed that osteoblasts and osteoclasts were the main populations in the single‐cell profiling of bone healing (Figure 1A). Differentially expressed genes (DEGs) in osteoblasts and osteoclast were enriched between non‐union and normal fracture healing patients (Figure 1B,C). Subsequently, the DEGs in osteoclast were further intersected with the set of genes enriched specifically under RANKL stimulation during osteoclast differentiation (deposited in the GEO database: GSE274540), thereby filtering out non‐specific responses and focusing on RANKL‐dependent regulatory candidates. A gene set of seven transcription factors (TF) was enriched (Figure 1D), as transcription factors are believed to play key roles in determining cell differentiation. To investigate the functional role of the candidate genes, we constructed specific siRNAs and transfected them into bone marrow‐derived macrophages (BMDMs), followed by RANKL stimulation, to evaluate their effects on osteoclast differentiation (Figure S1A). TRAP staining and quantitative analysis revealed that si*Zmat1* suppressed osteoclast differentiation of BMDMs (Figure S1B,C). Consistently with this, the mRNA levels of several key osteoclastic markers were significantly reduced (Figure S1D), including *Ctsk*, which encodes Cathepsin K, the major protease responsible for bone resorption [35]; *Itgb3*, which mediates osteoclast adhesion and activation on the bone surface [36]; and *Dcstamp*, a critical fusion factor required for the formation of multinucleated osteoclasts [37].

*Zmat1* was highly expressed in osteoclasts in nonunion bone compared to that in normal fracture healing (Figure 1E,F), indicating its inhibitory role in the healing process. *Zmat1* was upregulated during osteoclast differentiation after 72 h in the RNA‐seq data (Figure 1G). During osteoclastogenesis, increased levels of *Zmat1* mRNA were observed on days 3 and 5, which correlated with *Nfatc1* RNA expression in mice (Figure 1H,I). Since *Nfatc1* is a well‐established master transcription factor essential for osteoclast differentiation, its expression serves as a reliable indicator of osteoclast activation [2, 38]. These results strongly indicated that *Zmat1* plays an important role in osteoclastogenesis, and is related to impaired bone healing.

### 2.2 ZMAT1 Promotes Osteoclast Differentiation and Bone Resorption

**FIGURE 2:**
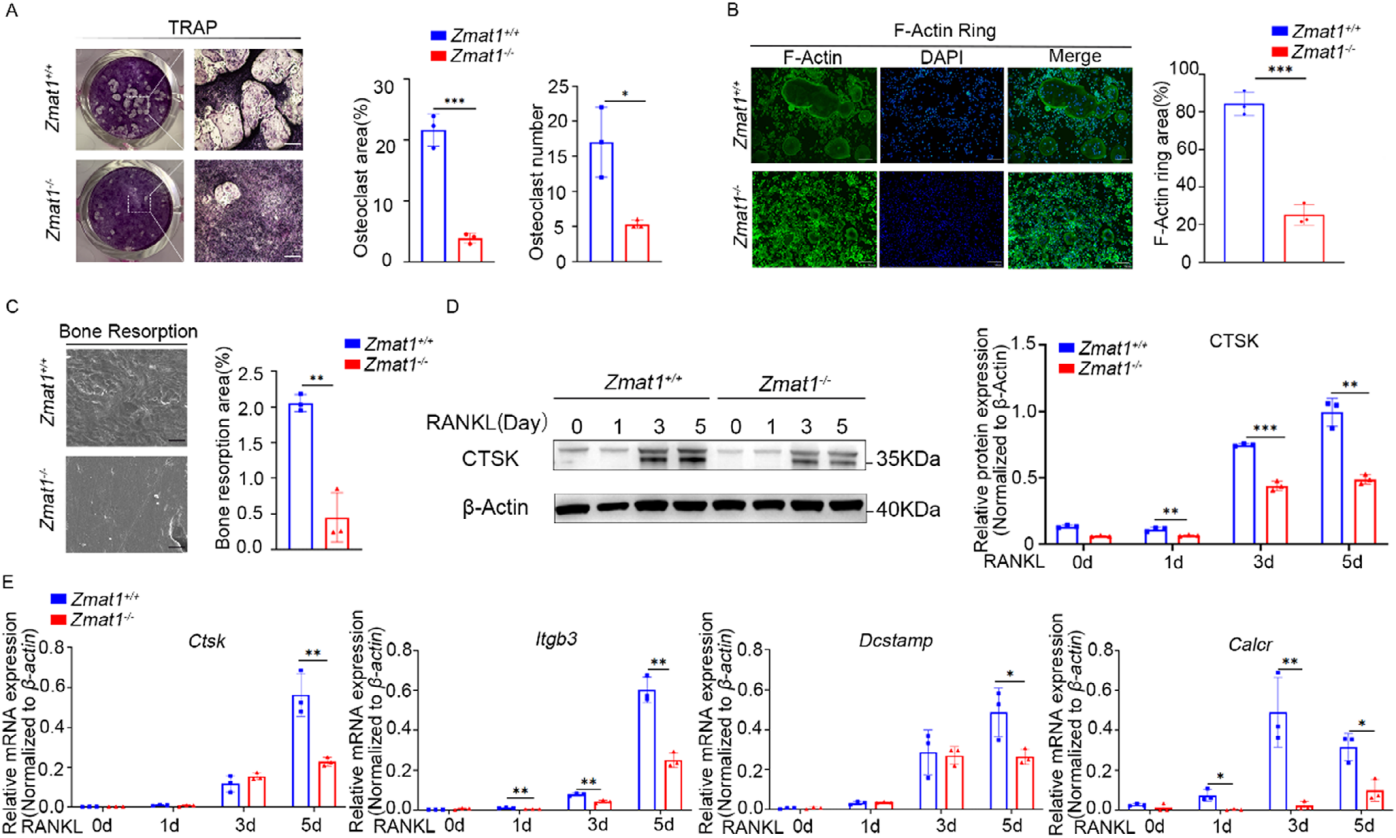
Osteoclast *Zmat1* deletion reduces osteoclastogenesis. (A) Representative image of TRAP staining in *Zmat1^+/+^* or *Zmat1^−/−^* mouse osteoclasts. Scale bar, 100 µm. Cumulative data show the area and number of osteoclasts from three independent experiments (right panel). (B) Mature osteoclasts differentiated from BMDMs of *Zmat1^+/+^* or *Zmat1^−/−^* mice were subjected to immunofluorescence staining for F‐actin. Nuclei stained with DAPI were observed blue and imaged using a fluorescence microscope. Scale bars, 100 µm. (C) BMDMs from *Zmat1^+/+^* or *Zmat1^−/−^* mice were cultured and differentiated into mature osteoclasts on bovine bone discs for 7 days. Mature osteoclasts were then completely removed from the discs, and the resorption pit areas were visualized under a light microscope (right panel). Scale bars, 100 µm. (D) Representative immunoblots of mouse Cathepsin K in *Zmat1^+/+^* or *Zmat1^−/−^* osteoclastogenesis. β‐Actin was used as a loading control. Right panels: Densitometric quantification of band intensity from three independent experiments. (E) RT‐qPCR analysis of *Zmat1^+/+^* and *Zmat1^−/−^* mouse *Ctsk*, *Igtb3*, *Dcstamp*, and *Calcr* mRNA during osteoclastogenesis on day 0, 1, 3, and 5. Normalized relative to *β‐actin* mRNA. *n* = 3. Osteoclast precursor cells were cultured with M‐CSF and RANKL on days 0, 1, 3, and 5. All bar graphs are presented as the mean ± SD. ^*^ *p* < 0.05; ^**^ *p* < 0.01; ^***^ *p* < 0.001; n.s. not significant by Student's *t* test and two‐way ANOVA for multiple comparisons.

The increase in *Zmat1* expression during osteoclast formation and nonunion prompted us to examine whether *Zmat1* regulates osteoclast differentiation. *Zmat1* siRNA effectively knocked down the expression of ZMAT1 in mouse BMDMs (Figure S2A), which resulted in a marked decrease in the number of TRAP^+^ multinuclear cells (MNCs) compared to that in the controls (Figure S2B,C). To further confirm the role of ZMAT1 in osteoclast differentiation, we generated *Zmat1^−/−^* mice (Figure S3A–C), indicating normal macrophages differentiation (Figure S3D,E). Compared with littermate controls, *Zmat1^−/−^* bone marrow cells generated a decreased number of osteoclasts with reduced area per well (Figure 2A). Accordingly, *Zmat1*‐depleted BMDMs failed to form large actin rings with areas comparable to those of control cells (Figure 2B). Likewise, when *Zmat1^−/−^* osteoclasts were plated onto bovine bone slices that are degraded by osteoclast enzymes, we observed significantly smaller and less frequent “resorption pits” in *Zmat1*‐deficient osteoclast cultures (Figure 2C), indicating defective osteoclast bone resorption activity. Upon RANKL stimulation for 0, 1, 3, and 5 days, *Zmat1* knockout significantly reduced the expression level of CTSK protein (Figure 2D). The mRNA levels of several key osteoclastic markers were significantly reduced, including *Ctsk*, *Itgb3*, *Dcstamp*, and *Calcr*, the calcitonin receptor that negatively regulates bone resorption [39] (Figure 2E).

To confirm the role of *Zmat1* in osteoclast differentiation, we overexpressed *Zmat1* in RAW264.7. Successfully transfected cells were enriched by sorting based on GFP expression (Figure S4A). In the presence of RANKL, *Zmat1*‐overexpressed RAW264.7 cells displayed increased expression of NFATc1 protein compared to the control group, with corresponding changes in mRNA levels. (Figure S4B,C). Additionally, the overexpression of *Zmat1* resulted in a significant increase in the area of TRAP^+^ MNCs (Figure S4D,E). Consistently, formation of the F‐actin ring also increased (Figure S4F,G). In addition, we found that *Zmat1* overexpression significantly enhanced the bone resorption capacity of osteoclasts (Figure S4H).

We collected monocytes from non‐osteoporotic individuals and induced their differentiation into osteoclasts, followed by qPCR analysis of *Zmat1* mRNA expression. *Zmat1* expression gradually increased following RANKL stimulation (Figure S5A). *Zmat1* knockdown inhibited human osteoclast differentiation (Figure S5B–D). Finally, monocytes from healthy individuals and osteoporosis patients were collected and analyzed for *Zmat1* mRNA expression. Compared with healthy controls, peripheral blood mononuclear cells (PBMCs) from patients with osteoporosis exhibited significantly higher levels of *Zmat1* expression (Figure S5E). These results suggest that ZMAT1 is a positive regulator of osteoclast formation, which increased the expression of osteoclast marker genes, actin ring formation and the bone‐resorbing activity of mature osteoclasts.

### 2.3 *Zmat1^−/−^* Mice are Protected From OVX‐Induced Osteoporosis

**FIGURE 3:**
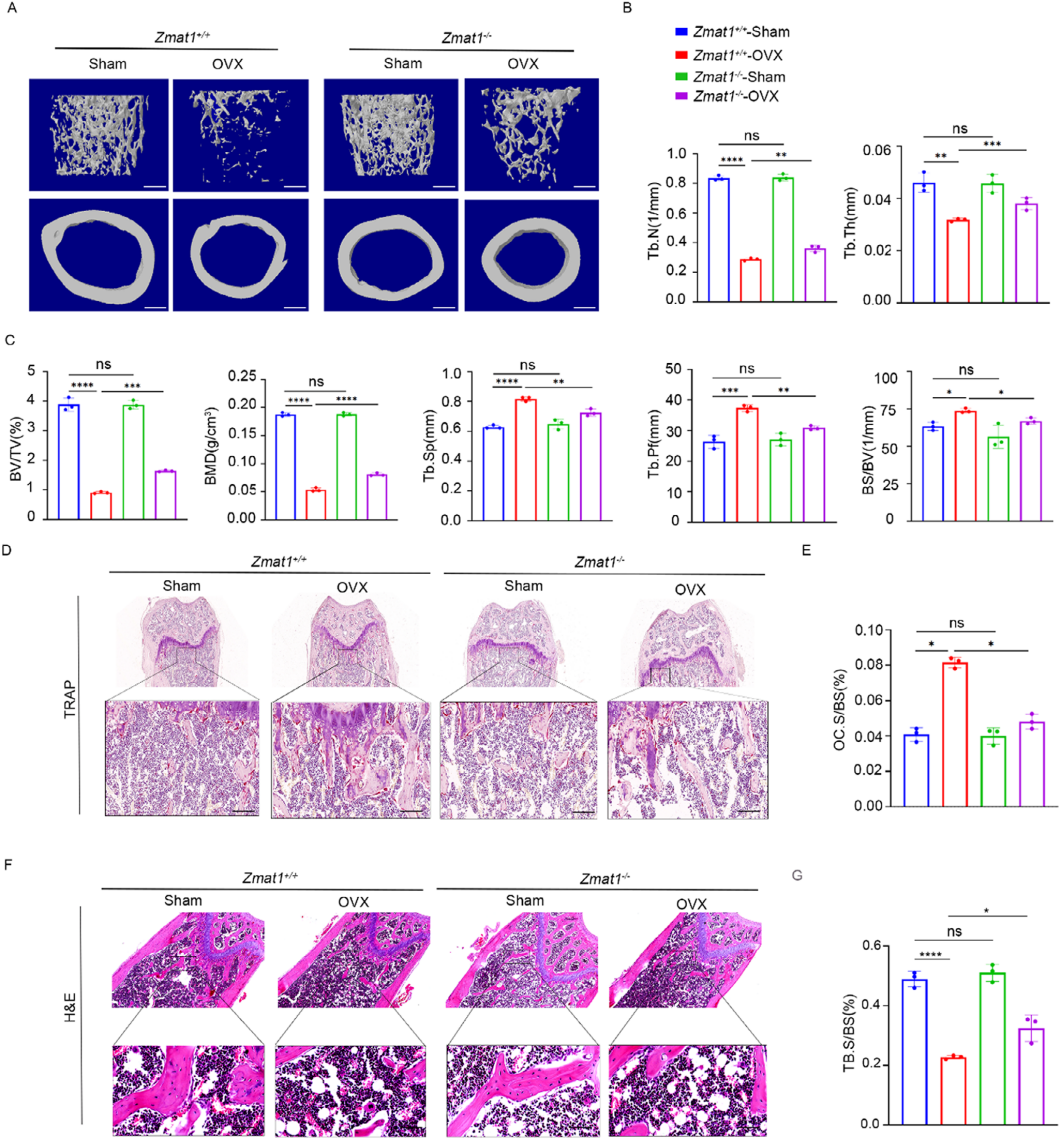
*Zmat1^−/−^* mice show increased bone mass after OVX with decreased osteoclast numbers. (A) Representative micro‐CT images of whole femoral trabecular (top and bottom) and cortical (middle) bones from *Zmat1^−/−^* and *Zmat1^+/+^* mice after OVX for 8 weeks. Scale bars, 100 µm. (B) Histomorphometric analysis of the trabecular bones from (A), including trabecular number (Tb.N) and trabecular thickness (Tb.Th). *n* = 3 per group. (C) Histomorphometric analysis of trabecular bones from A, including bone volume per tissue volume (BV/TV), bone mass density (BMD), trabecular separation (Tb.Sp), trabecular bone pattern factor (Tb.Pf), and bone surface per bone volume (BS/BV). *n* = 3 per group. (D) TRAP staining of femurs from sham and OVX mice compared to *Zmat1^−/−^* or *Zmat1^+/+^* mice (*n* = 3). Scale bars represent 200 µm. (E) Quantification of osteoclast surface per bone surface (OC. S/BS, %) in (D) (*n* = 3). (F) H&E staining of femurs of Sham, OVX over *Zmat1^−/−^* or *Zmat1^+/+^* mice (*n* = 3). Scale bars represent 200 µm. (G) Quantification of the trabecular bone surface per bone surface (Tb.BS/BS, %) in (F) (*n* = 3). All bar graphs are presented as the mean ± SD. ^*^ *p* < 0.1; ^**^ *p* < 0.01; ^***^ *p* < 0.001; ^****^ *p* < 0.0001; n.s. not significant by one‐way ANOVA for multiple comparisons.

To investigate the role of *Zmat1* in vivo, we conducted ovariectomized (OVX) in *Zmat1^+/+^* and *Zmat1^−/−^* littermate mice. After 8 weeks, trabecular number (Tb.N) and trabecular thickness (Tb.Th) were increased significantly in *Zmat1^−/−^* ‐OVX mice (Figure 3A,B). Also, bone volume per tissue volume (BV/TV) increased in *Zmat1^−/−^‐*OVX mice compared with that in *Zmat1^+/+^*‐OVX mice (Figure 3C). On the contrary, *Zmat1^−/−^*‐OVX mice had increased bone mineral density (BMD) in trabecular bones while trabecular separation (Tb.Sp), trabecular pattern factor (Tb.Pf) and bone surface area to bone volume ratio (BS/BV) showed a mild decrease in *Zmat1^−/−^*‐OVX mice compared with those in *Zmat1^+/+^* mice, indicating enhanced of mechanical intension (Figure 3C). Compared to *Zmat1^+/+^* mice, *Zmat1^−/^* ^−^‐OVX mice had fewer osteoclasts (Figure 3D,E) and exhibited a higher density of trabecular bones (Figure 3F,G). Notably, there was no significant difference in the bone mass between *Zmat1^+/+^* and *Zmat1^−/−^* mice in the sham group. These data confirmed that *Zmat1* knockout prevented bone loss in female mice exposed to estrogen deficiency.

### 2.4 *Zmat1* Deficiency Inhibits OVX‐Induced Bone Loss by Impairing Osteoclasts

**FIGURE 4:**
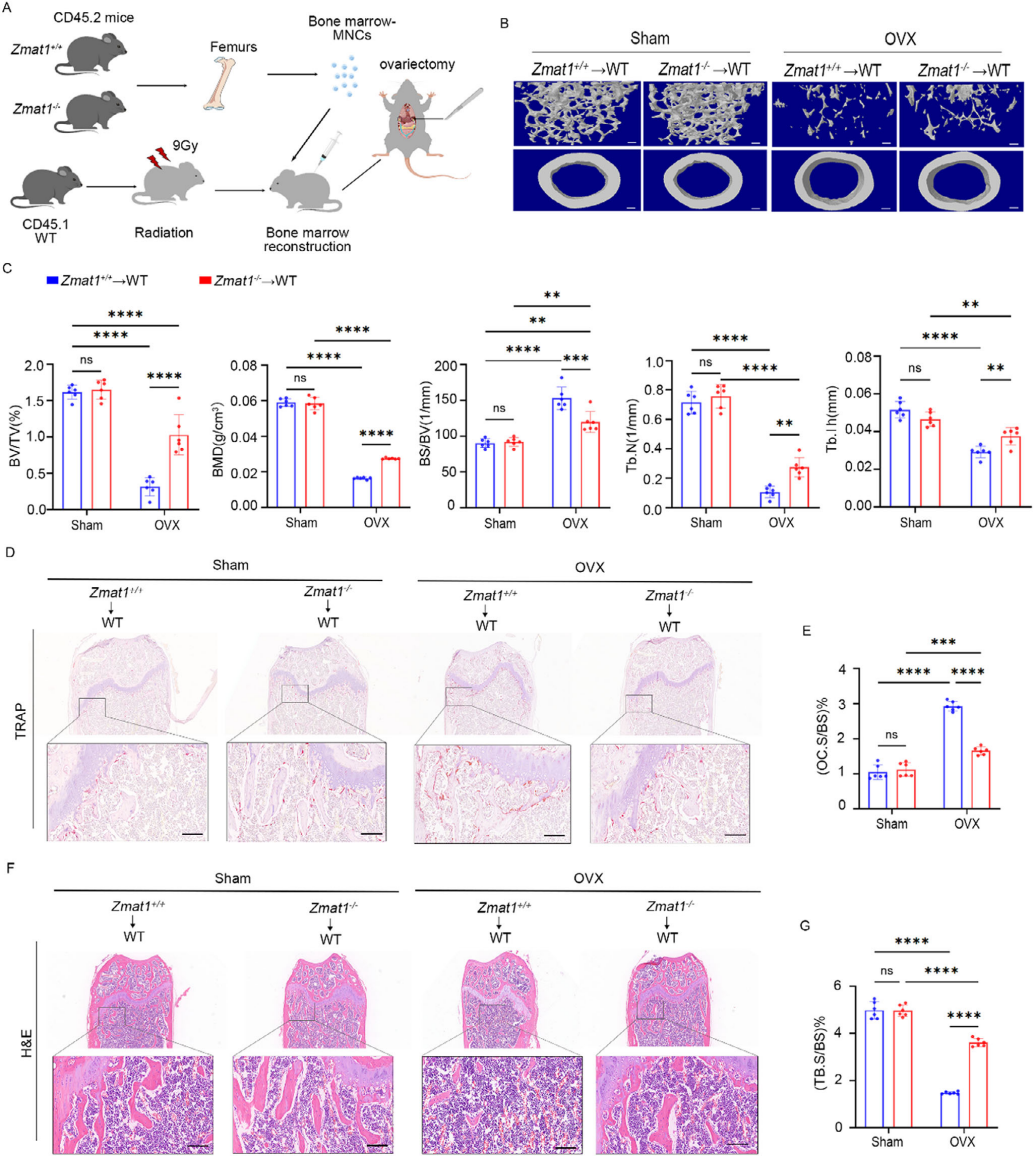
*Zmat1* deletion improves ovariectomy‐induced bone loss by inhibiting osteoclast activity. (A) Schematic of the murine model for bone marrow transplant and ovariectomy‐induced bone loss in mice. (B) Representative reconstructed 3D µCT images of the proximal tibia in myeloid *Zmat1* knockout mice. Scale bars, 100 µm. (C) Quantification of BV/TV, BMD, BS/BV, Tb.N, and Tb.Th, from µCT images. *n* = 6 per group. (D) TRAP staining of the femora from OVX mice. Scale bars, 200 µm. (E) Quantification of osteoclast surface per bone surface (OC. S/BS, %) in (D). *n* = 6 per group. (F) H&E staining of the femora from OVX‐mice. Scale bars, 200 µm. (G) Quantification of trabecular bone surface per bone surface (Tb.BS/BS, %) in (F). *n* = 6 per group. All bar graphs are presented as the mean ± SD. ^***^ *p* < 0.001; ^****^ *p* < 0.0001; n.s. not significant by two‐way ANOVA for multiple comparisons.

Bone homeostasis is dependent on the coupling between bone formation and resorption. The inhibited bone loss in *Zmat1^−/−^* mice may be caused by decreased osteoclasts or increased osteoblasts generation. Osteoclasts are bone‐resorbing cells that differentiate from hemopoietic cells of the monocyte/macrophage lineage. To determine whether *Zmat1* regulates postmenopausal osteoporosis in mice mainly through osteoclasts, we used bone marrow chimeric mice (Figure 4A; Figure S6A,B). CD45.1 mice reconstituted with *Zmat1*‐deficient bone marrow cells (*Zmat1^−/−^* →WT) showed an increased trabecular bone density and thicker cortical bone in the femur (Figure 4B), compared with those reconstituted with *Zmat1^+/+^* bone marrow cells (*Zmat1^+/+^* →WT) after ovariectomy. Assessment of quantitative morphometric bone parameters revealed significant increase in BV/TV, BMD, Tb.N, and Tb.Th, and a marked reduction in BS/BV in *Zmat1^−/−^* bone marrow chimeric mice (Figure 4C), consistent with the osteoporotic phenotype. Histological and morphological evaluation of femoral sections in mice using TRAP staining (Figure 4D) and H&E staining (Figure 4E) revealed that, compared to *Zmat1^+/+^* mice, *Zmat1^−/−^* chimeric mice exhibited greater bone mass (Figure 4F,G). Further immunohistochemical analysis of osteocalcin (OCN) expression revealed no significant difference in the expression of the osteoblast marker OCN (Figure S6C,D). Taken together, the strongly increased bone mass observed in the *Zmat1^−/−^* bone marrow‐reconstructed OVX experiments was due to defects in osteoclast differentiation.

### 2.5 *Zmat1* Inhibits Bone Remodeling by Repressing Osteoblastogenesis

Bone remodeling mainly depends on the balance between osteoblasts and osteoclasts. During bone remodeling after a fracture, osteoclasts are constantly digested, absorbed into bone segments, and remodeled. In addition, osteoblastic differentiation of bone marrow‐derived mesenchymal stem cells (BMSCs) is important for bone defect regeneration. Human BMSCs obtained from clinical samples were subjected to osteogenic differentiation. We observed that *Zmat1* expression gradually decreased during osteogenic differentiation (Figure S7A). Knockdown of *Zmat1* promoted the osteogenic differentiation of human osteoblasts (Figure S7B,C).

**FIGURE 5:**
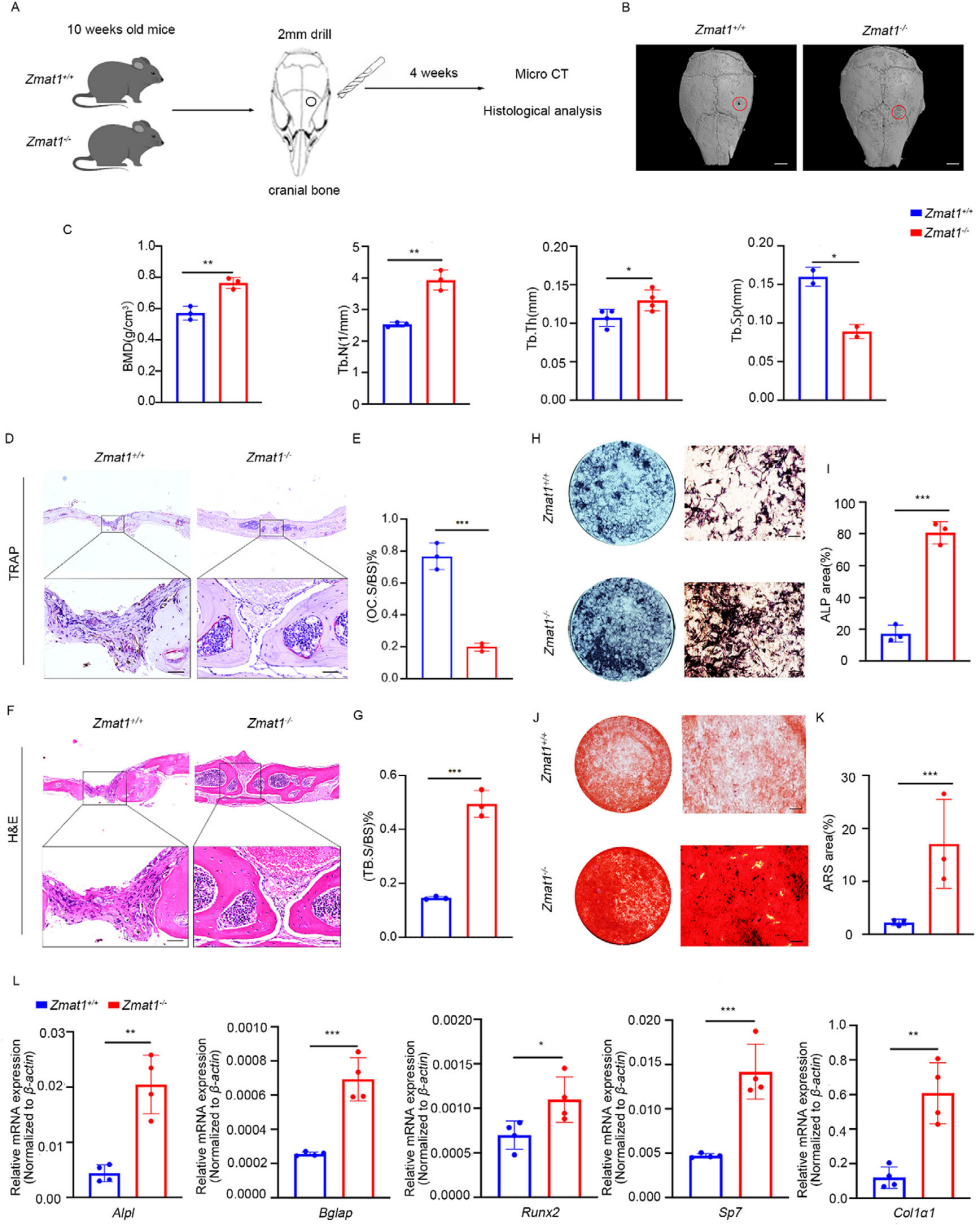
*Zmat1* deficiency promotes osteoblastogenesis and enhances bone remodeling. (A) Schematic diagram showing the of *Zmat1^+/+^* and *Zmat1^−/−^* mice undergoing cranial bone operation of burr hole. (B) Representative micro‐CT images of calvarial bone defects in 10‐week‐old *Zmat1^+/+^* and *Zmat1^−/−^* mice after surgical induction for 4 weeks. Scale bars, 100 µm. (C) Quantitative measurements of BMD, Tb.N, Tb.Th, and Tb.Sp. (D) Representative images of TRAP staining of skull bone sections of 14‐week‐old *Zmat1^+/+^* and *Zmat1^−/−^* mice after surgery. Scale bars, 200 µm. (E) Quantification of osteoclast surface per bone surface (OC. S/BS, %) in (D). *n* = 3 per group. (F) Representative images of H&E staining of cranial bone sections of 14‐week‐old *Zmat1^+/+^* and *Zmat1^−/−^* mice undergoing surgery. Scale bars, 200 µm. (G) Quantification of trabecular bone surface per bone surface (Tb.BS/BS, %) in (F). *n* = 3 per group. (H) Representative images of ALP staining on 21 days of osteoblasts from *Zmat1^+/+^* and *Zmat1^−/−^* mice. Scale bars, 100 µm. (I) Quantification of ALP‐positive area per well for (H). *n* = 3 per group. (J) Representative images of ARS staining on 21 days of osteoblasts from *Zmat1^+/+^* and *Zmat1^−/−^* mice. Scale bars, 100 µm. (K) Quantification of ARS‐stained mineralized area per well for (J). *n* = 3 per group. (L) qPCR analysis of osteogenic biomarker genes *Alpl*, *Bglap*, *Runx2*, *Sp7*, *Col1α1* in osteoblasts with osteogenic induction. All bar graphs are presented as the mean ± SD. ^*^ *p* < 0.05; ^**^ *p* < 0.01; ^***^ *p* < 0.001; n.s. not significant by Student's *t* test.

To investigate whether *Zmat1* was also involved in osteoblast differentiation, we surgically created skeletal defects by drilling holes in the calvaria (Figure 5A). Micro‐CT analysis consistently showed that the cortical gaps in *Zmat1^−/−^* mice were almost completely bridged after four weeks, whereas those in *Zmat1^+/+^* mice were only partially filled (Figure 5B). In addition, BMD, Tb. N and Tb. Th, of the mineralized calli of *Zmat1‐/‐* mice were significantly higher than those of *Zmat1^+/+^* controls, whereas Tb.Sp decreased after *Zmat1* was deleted (Figure 5C). Compared with *Zmat1^+/+^* mice, *Zmat1^−/−^* mice exhibited a decreased number of osteoclasts and a significant increase in bone mass (Figure 5D–G). To confirm whether *Zmat1* deficiency inhibits bone mass loss by regulating bone formation, we evaluated the role of *Zmat1* in osteogenesis. Differentiation measured by ALP staining and mineralization measured by ARS staining of *Zmat1*‐deficient osteoblasts were significantly enhanced (Figure 5H–K). In *Zmat1* deficiency cells, the mRNA expression levels of several key osteogenic markers were markedly increased, including *Alpl*, a critical enzyme for bone matrix mineralization; *Bglap*, which facilitates calcium binding and bone matrix calcification; *Runx2* and *Sp7*, core transcription factors essential for osteoblast differentiation; and *Col1a1*, a major structural component required for maintaining the mechanical integrity of bone tissue [40, 41]. These results confirmed that *Zmat1* deficiency enhanced the osteogenic potential of osteoblasts (Figure 5L).

To clarify the role of osteoblastogenesis, we performed immunohistochemical staining for OCN, a marker of osteoblast differentiation, in tibial sections from both *Zmat1^+/+^* and *Zmat1^−/−^* mice. The OCN‐positive staining in the Sham‐*Zmat1^−/−^* group was not significantly different from that in the Sham‐*Zmat1^+/+^* group (Figure S8A,B). After OVX, the OCN‐positive area was also greater in the *Zmat1^−/−^* group compared with the *Zmat1^+/+^* group. In summary, these findings suggested that the increased bone mass observed in *Zmat1*‐deficient mice after OVX was due to suppressed osteoclastogenesis accompanied by enhanced osteoblast activity. In summary, our findings suggest that *Zmat1* deficiency inhibits osteoclastogenesis and promotes osteoblast differentiation.

### 2.6 ZMAT1 Deficiency Inhibits Osteoclastic Differentiation by Decreasing YAP1 Protein Stability

**FIGURE 6:**
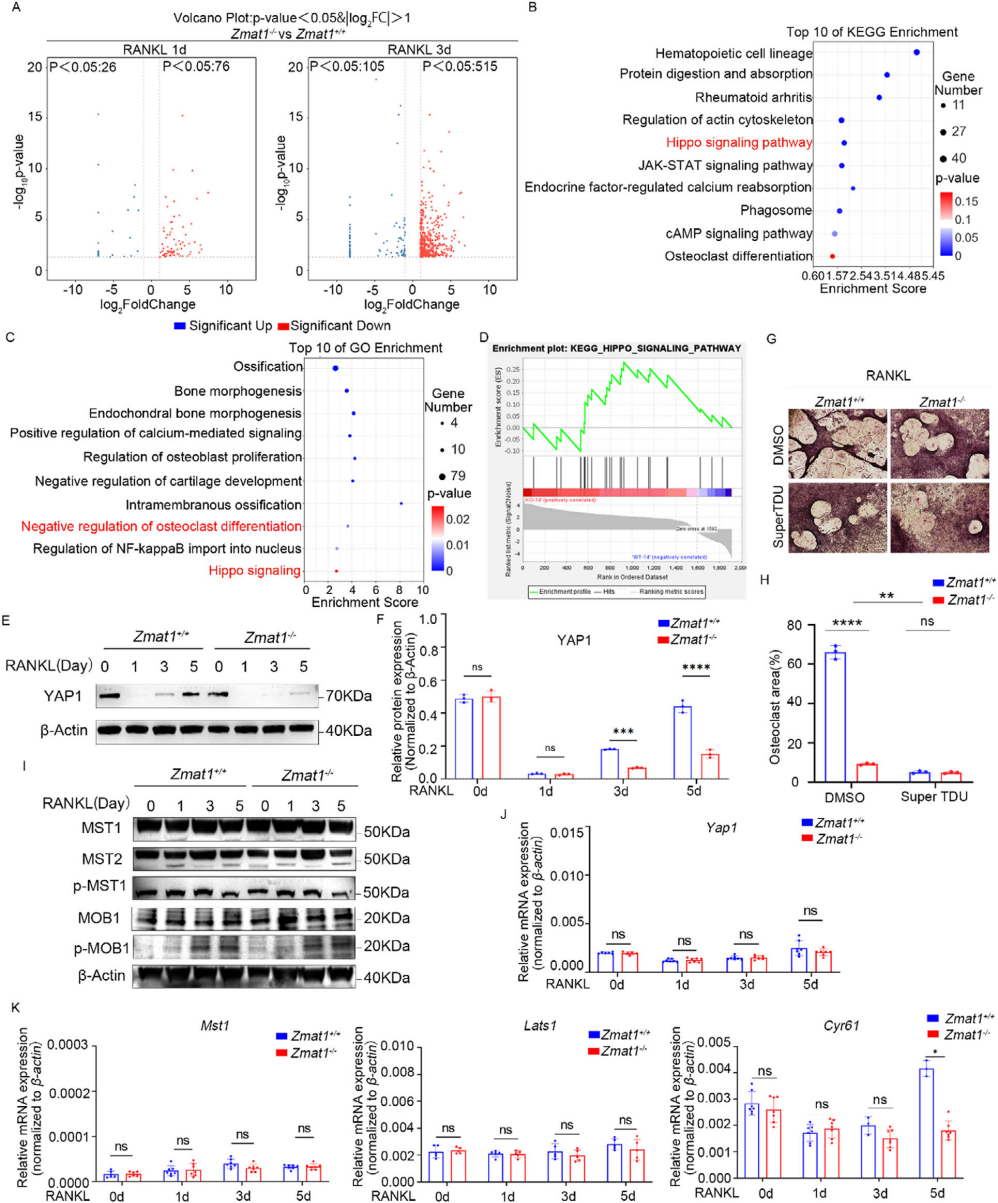
Hippo signaling is essential for ZMAT1‐mediated osteoclast differentiation. (A) Volcano plots of differentially expressed genes (DEGs) in BMDMs from *Zmat1^+/+^* and *Zmat1^−/−^* mice treated with 100 ng/mL RANKL and 50 ng/mL M‐CSF for 1 day (left) and 3 days (right). (B) KEGG enrichment analysis of the DEGs in the BMDMs. (C) GO enrichment analysis of the DEGs in the BMDMs. (D) DEGs were analyzed using GSEA. (E) Representative immunoblotting analysis of YAP1 protein expression in BMDMs treated with 100 ng/mL RANKL and 50 ng/mL M‐CSF followed by the indicated time. (F) Densitometric quantification of phosphorylated YAP1 band intensities from three independent experiments. β‐Actin was used as a loading control. (G) The cells were treated in medium added to 10 µM Super TDU with 100 ng/mL RANKL and 50 ng/mL M‐CSF. TRAP staining was followed in the indicated way. Scale bars, 100 µm. (H) Cumulative data showing area of osteoclasts from three independent experiments of (G). (I) Representative immunoblotting analysis of Hippo signaling pathway protein expression in BMDMs treated with 100 ng/mL RANKL and 50 ng/mL M‐CSF followed by the indicated time. (J,K) qPCR analysis of *Yap1*, *Mst1*, *Lats1* and *Cyr61* in osteoclastogenesis from *Zmat1^+/+^* and *Zmat1^−/−^* mice as indicated by medium and time. All bar graphs are presented as the mean ± SD. ^*^ *p* < 0.05; ^**^ *p* < 0.01; ^****^ *p* < 0.000 1; n.s. not significant by two‐way ANOVA.

To gain insight into the underlying mechanisms by which *Zmat1* suppresses osteoclast differentiation of BMDMs, we performed an unbiased whole transcriptome analysis to identify the genes affected by *Zmat1*. A total of 105 genes was significantly downregulated, and 515 genes were upregulated in *Zmat1^−/−^* osteoclastic cells compared with control groups 3d after RANKL stimulation (Figure 6A); Fewer differences were observed 1d after RANKL stimulation (Figure 6A). Kyoto Encyclopedia of Genes and Genomes (KEGG) enrichment analysis revealed that signaling pathways related to rheumatoid arthritis, regulation of the actin cytoskeleton, osteoclast differentiation, and the Hippo signaling pathway were strongly enriched in the differentially expressed mRNAs (Figure 6B). Gene ontology (GO) analysis also showed that *Zmat1* was related to osteoclast differentiation, bone morphogenesis, and Hippo signaling (Figure 6C). Gene set enrichment analysis (GSEA) confirmed that the Hippo signaling pathway was significantly upregulated in *Zmat1*‐deficient osteoclasts (Figure 6D). These data suggest that *Zmat1* plays an important role in osteoclast differentiation by regulating the Hippo signaling pathway.

Therefore, we tested whether *Zmat1* regulates osteoclastic differentiation via the Hippo signaling pathways. YAP1 is a key effector in Hippo signaling, and the activation of the Hippo pathway leads to YAP1 phosphorylation, cytoplasmic retention, and subsequent degradation [42]. *Zmat1* deficiency significantly suppressed the total YAP1 protein levels after RANKL stimulation (Figure 6E,F). We also observed a sudden and unexpected disappearance of YAP1 at day 1 both in *Zmat1^+/+^* and *Zmat1^−/−^* cells. To investigate the underlying mechanisms, we stimulated BMDMs with RANKL and assessed YAP1 expression at different times points. We observed that YAP1 protein expression gradually decreased from 0 to 24 h (Figure S9A). To address this concern, we performed cycloheximide (CHX) chase assays and proteasome inhibition experiments using MG132 to investigate the mechanism underlying YAP1 downregulation during osteoclast differentiation. CHX chase assays revealed that YAP1 underwent accelerated degradation in the presence of RANKL compared to that in the vehicle group (Figure S9B). Importantly, treatment with the proteasome inhibitor MG132 largely prevented RANKL‐induced reduction of YAP1, confirming that YAP1 degradation is mediated through the ubiquitin‐proteasome pathway (Figure S9C). Collectively, these data demonstrated that the rapid loss of YAP1 during the early stage of osteoclast differentiation was attributable to proteasomal degradation.

To investigate whether YAP1 regulates osteoclastic differentiation, we treated the osteoclast induction system with SuperTDU, an inhibitor of the YAP1‐TEAD interaction, and examined the expression of osteoclast‐related genes such as NFATC1, MMP9, and CTSK (Figure S10A–E). The results showed that the YAP1 inhibitor SuperTDU significantly suppressed osteoclast differentiation. To further confirm that *Zmat1* regulated OC differentiation through YAP1, we treated *Zmat1^−/−^* and *Zmat1^+/+^* cells with SuperTDU, a small‐molecule inhibitor of YAP1, during osteoclastogenesis. Subsequent TRAP staining revealed that SuperTDU rescued the pro‐osteoclastogenic effects of *Zmat1* (Figure 6G,H). These results indicate that YAP1 was responsible for the effects of *Zmat1* during osteoclastogenesis.

We aimed to elucidate the specific role of *Zmat1* in the Hippo signaling pathway, we have included additional immunoblotting analyses of key components of the Hippo signaling cascade, the protein expression levels of these core Hippo signaling pathway molecules were not significantly different in *Zmat1^−/−^* cells compared to *Zmat1^+^/^+^* cells (Figure 6I), suggesting that ZMAT1 does not regulate osteoclast differentiation by activating the entire Hippo signaling pathway. Moreover, mRNA levels of *Yap1*, *Mst1*, and *Lats1* remained unchanged (Figure 6J,K). Thus, *Zmat1* may modulate the expression of YAP1 posttranslationally rather than at the transcriptional level. Consistent with the altered YAP1, the expression of the YAP1‐associated target gene cysteine‐rich angiogenic inducer 61 (*Cyr61*) was modulated at specific stages of osteoclast differentiation (Figure 6K), confirming the suppressed translocation of YAP1 into the nuclei of *Zmat1^−/−^* cells. These results imply that *Zmat1* deficiency inhibits osteoclastic differentiation by post‐transcriptionally regulating YAP1 protein expression.

Furthermore, in vitro osteogenic differentiation assays demonstrated that treatment with the YAP1 inhibitor markedly impaired bone matrix mineralization, as evidenced by reduced ALP and ARS staining (Figure S11A–C). In parallel, the analysis of osteogenic differentiation markers indicated that treatment with the YAP1 inhibitor SuperTDU significantly suppressed osteoblast differentiation (Figure S11D–F). These results showed that Super TDU treatment significantly downregulated the expression of osteoclast‐related genes and also reduced the transcriptional levels of osteoblast‐related genes. Interestingly, we found that *Zmat1* also regulated osteoblast differentiation in a YAP1‐dependent manner (Figure S11G,H).

### 2.7 *Zmat1* Transcriptionally Down‐Regulated E3 Ligase TRIM46

**FIGURE 7:**
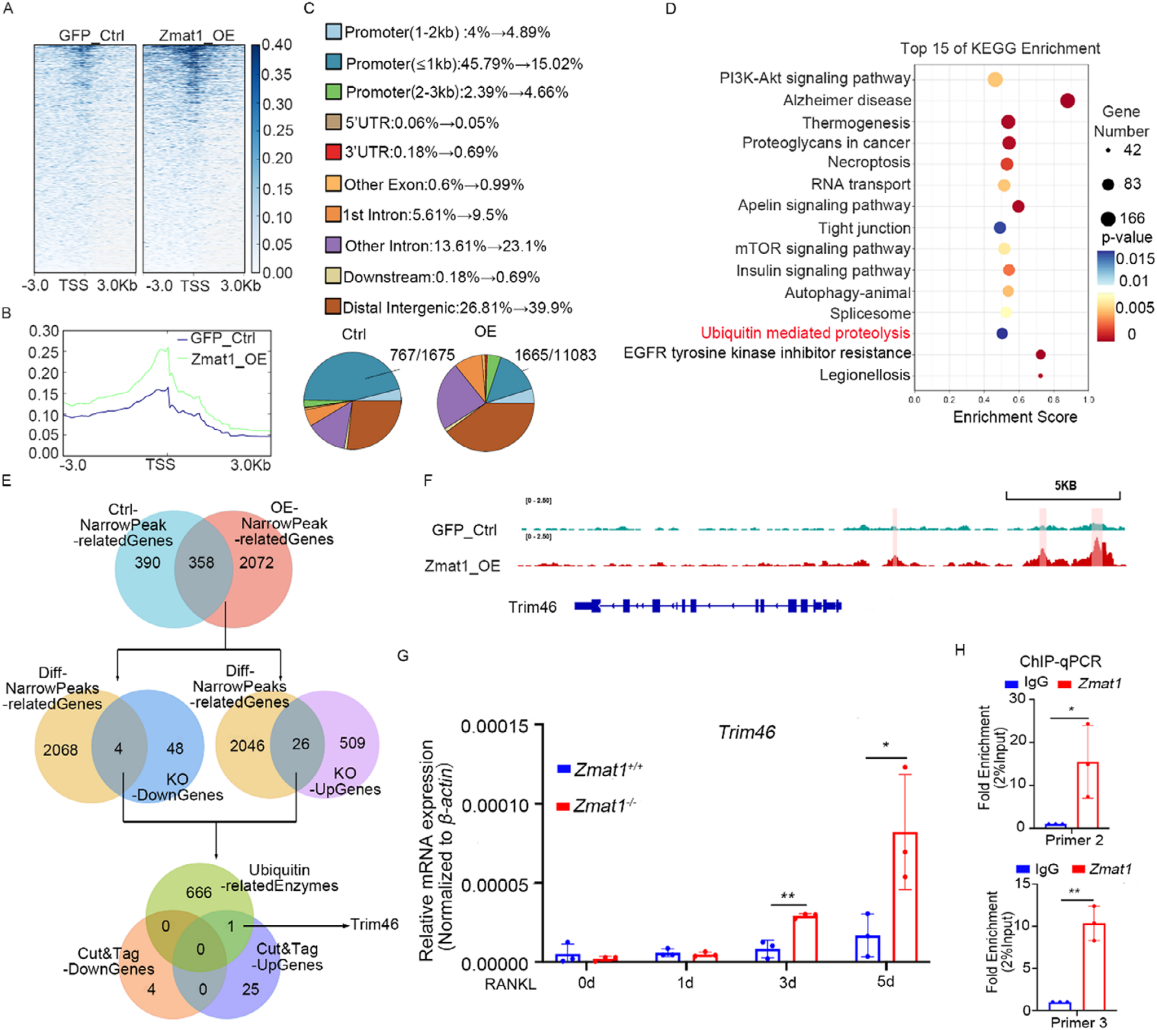
ZMAT1 inactivates *Trim46* transcription. (A) CUT&Tag density heatmap of Flag‐*Zmat1* enrichment in RAW264.7 cells within 3 kb around the transcription start site (TSS). (B) CUT&Tag signal height and position of *Zmat1* and control group relative to TSSs for all genes in RAW264.7 cells. (C) The genomic distribution of *Zmat1* CUT&Tag peaks in RAW264.7 cells. (D) KEGG enrichment analysis of the narrow peaks in the BMDMs. (E) Bioinformatics analysis identified *Trim46* as a downstream target of *Zmat1*. (F) IGV tracks for *Trim46* from *Zmat1* CUT&Tag analysis. (G) qPCR analysis of *Trim46* expression in osteoclastogenesis derived from *Zmat1^+/+^* and *Zmat1^−/−^* mice. (H) ChIP‐qPCR assay of the *Zmat1* status at the *Trim46* promoter and its upstream and downstream regions after *Zmat1* overexpression in RAW264.7 cells. All bar graphs are presented as the mean ± SD. ^*^ *p* < 0.1; ^**^ *p* < 0.01; n.s. not significant by Student's *t* test and two‐way ANOVA.

*Zmat1* could regulate gene transcription [23]. We hypothesised that ZMAT1 functions in osteoclastogenesis as either a transcriptional promoter or suppressor. CUT&Tag sequencing revealed that *Zmat1*‐binding sites were mainly distributed near the transcription start site (TSS) (deposited in the GEO database: GSE274533; Figure 7A–C), suggesting that it functions as a regulator of gene transcription. KEGG analysis revealed different pathways in *Zmat1* overexpressing cells (Figure 7D). Moreover, to identify DEGs directly regulated by *Zmat1*, we analyzed narrow peaks related genes in CUT&Tag‐seq and the DEGs in RNA‐seq. These genes were further integrated with the DEGs in the RNA‐seq between *Zmat1^+/+^* and *Zmat1^−/−^* osteoclastogenesis (Figure 7E). Finally, four *Zmat1*‐induced and 26 *Zmat1*‐repressed binding genes were identified.

Given that protein ubiquitination and subsequent ubiquitin‐proteasome degradation are the major mechanisms for the post‐translational regulation of proteins, we hypothesized that *Zmat1* might regulate the transcription of a ubiquitinase or deubiqutinase for the posttranslational regulation of YAP1. In addition, ubiquitin‐mediated proteolysis was significantly enriched in the KEGG pathway analysis to differentiated peak related genes in CUT&Tag‐seq (Figure 7D). We integrated 30 *Zmat1*‐regulated binding genes with ubiquitin‐related enzymes library (including ligases and deubiquitinases). Among the 30 *Zmat1* directly regulated genes, only one E3 ubiquitin ligase gene, *Trim46*, presented *Zmat1*‐binding signals in its promoters and increased mRNA expressions in *Zmat1* deficient cells (Figure 7E), strongly suggesting its role in *Zmat1* mediated YAP1 regulation. In the integrative genomics viewer (IGV) analysis results of CUT&Tag, there existed narrow peaks bound by ZMAT1 within the *Trim46* promoter (Figure 7F).

Consistent with these results, *Zmat1* deficiency upregulated *Trim46* mRNA expression during osteoclastogenesis (Figure 7G), which suggested the transcriptional repressing function of ZMAT1. ChIP‐qPCR also confirmed the direct enrichment of *Zmat1* at the promoter of the *Trim46* gene (Figure 7H). These data indicated that *Zmat1* binds to the promoter of the *Trim46* gene and suppresses its transcription, which may play a key role in the subsequent regulation of YAP1 degradation and osteoclastogenesis.

### 2.8 TRIM46 Promotes YAP1 Degradation Through K48 Ubiquitination

**FIGURE 8:**
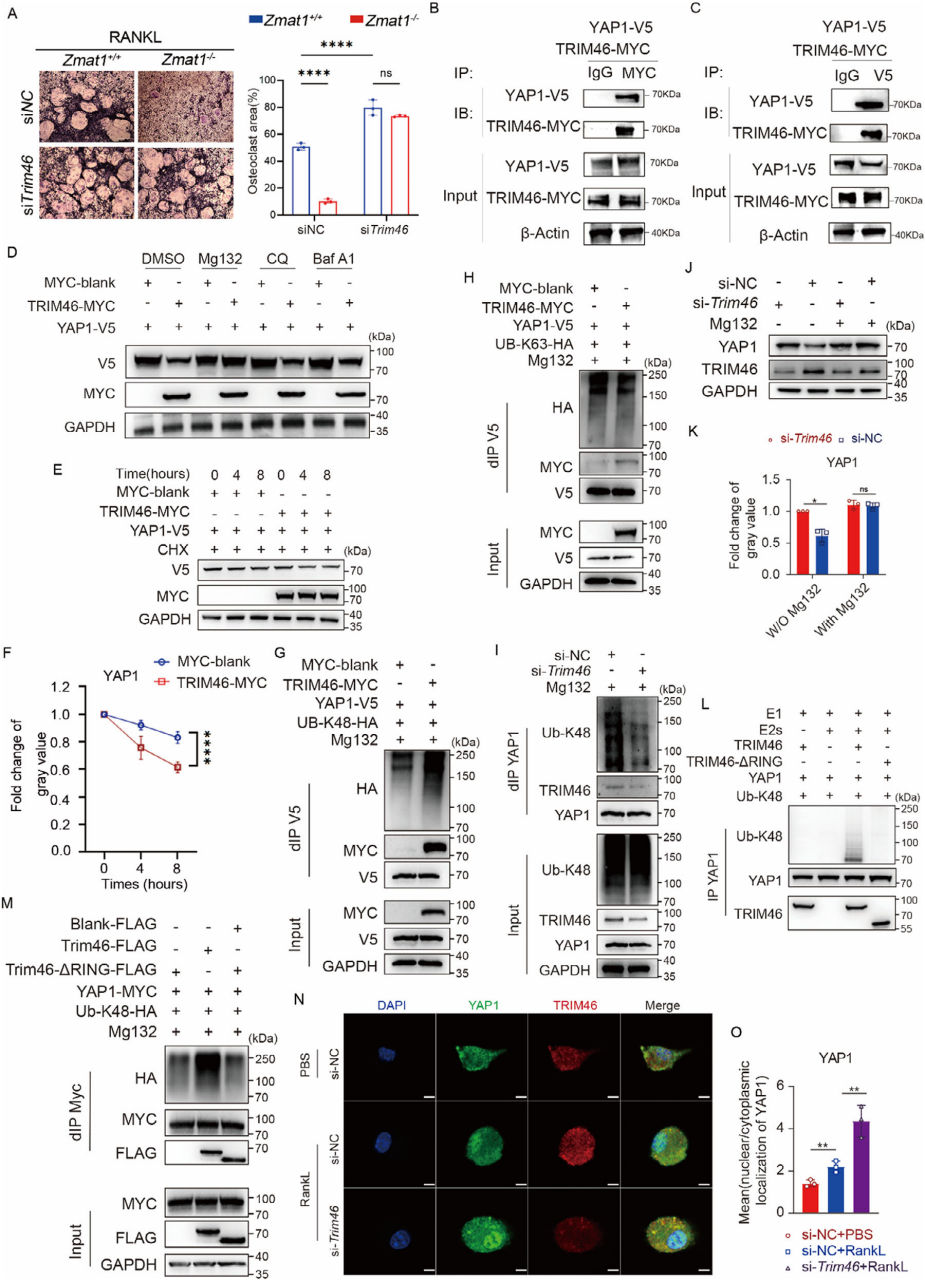
TRIM46 promotes YAP1 degradation through K48 ubiquitination. (A) BMDMs were transfected with siRNA targeting *Trim46* for 48 h, afterwards, BMDMs treated with 100 ng/mL RANKL and 50 ng/mL M‐CSF for 5 days. The left images show TRAP staining results. Quantification of the TRAP‐positive multinuclear cells surface is in the right panel. Scale bars, 100 µm. (B,C) HEK293 T cells were transfected with V5‐*Yap1*, Myc‐*Trim46*. V5 immunoprecipitates or Myc immunoprecipitates were further analyzed by immunoblotting with corresponding antibodies. (D) HEK293 T cells were transfected with or without V5‐*Yap1*, Myc‐*Trim46*, and HA‐K48‐ubiquitin plasmids for 48 h, and the cells were lysed, and proteins were detected by WB. (E) Cycloheximide (CHX) chase assay showing the effect of TRIM46 on YAP1 protein stability. HEK293T cells were co‐transfected with YAP1‐V5 and either MYC‐blank or TRIM46‐MYC, followed by CHX treatment. Cell lysates were collected at 0 h, 4 h, and 8 h. (F) Quantification of YAP1 protein levels in (E). YAP1 band intensities were normalized to GAPDH. (G) HEK293 T cells were transfected with or without V5‐*Yap1*, Myc‐*Trim46*, and HA‐K48‐ubiquitin plasmids for 48 h, and the cells were lysed, and proteins were detected by WB. (H) HEK293 T cells were transfected with or without V5‐*Yap1*, Myc‐*Trim46*, and HA‐K63‐ubiquitin plasmids for 48 h, and the cells were lysed, and proteins were detected by WB. (I) BMDMs were transfected with 20 µM small interfering RNA (siRNA) targeting *Trim46* to decrease its mRNA expression, followed by assessment of alterations in K48‐linked ubiquitination of YAP1. (J,K) BMDMs were transfected with 20 µM *Trim46*‐specific siRNA to suppress its mRNA expression, followed by assessment and quantification of YAP1 protein levels. (L) Using a pre‐mixed ubiquitination assay system containing E1, E2s and reaction buffer, GST pull‐down purified wild‐type TRIM46 (5 µM) or its RING domain deletion mutant (TRIM46‐ΔRING, 5 µM) was incubated with YAP1 substrate. Ubiquitination reactions were analyzed by immunoblotting with anti‐K48‐linked ubiquitin antibody. (M) In vivo ubiquitination assay of YAP1 by TRIM46 truncation mutants. (N) Immunofluorescence analysis of primary bone marrow‐derived macrophages (BMDMs) demonstrates co‐localization of YAP1 and TRIM46. BMDMs were cultured for 3 days and then treated with either PBS or RANKL. Scale bar: 2.5 µm. (O) Statistical analysis of reduced nuclear‐to‐cytoplasmic ratio of YAP1 upon *Trim46* knockdown. ^**^ *p* < 0.05; n.s. not significant by Student's *t* test and two‐way ANOVA.

To explicitly determine whether the impaired osteoclast differentiation observed in *Zmat1*‐deficient mice is caused by the upregulation of TRIM46, we performed rescue experiments. As expected, *Zmat1* deficiency alone significantly suppressed osteoclast formation compared to *Zmat1^+/+^* (Figure 8A). Strikingly, silencing *Trim46* in *Zmat1* ^−^/^−^ BMDMs significantly restored both the area and number of TRAP‐positive multinucleated cells to levels comparable to those of *Zmat1^+/+^* cells (Figure 8A). Consistent, overexpression of TRIM46 greatly rescued the effect of Zmat1 deficiency (Figure S12A), indicating that TRIM46 was necessary for the effect of ZMAT1.

Moreover, to verify whether the enhanced osteoclastogenesis resulting from *Trim46* depletion relies on YAP1 activity, we treated the cells with the YAP1 inhibitor SuperTDU (Figure S12B). Notably, after the addition of SuperTDU, the level of osteoclast formation showed no significant differences with different TRIM46 expression (Figure S12B,C), suggesting the effect of TRIM46 mainly dependents on YAP1 activity. Collectively, these data provide compelling evidence that ZMAT1 promotes osteoclastogenesis by preventing the TRIM46‐mediated degradation of YAP1.

To study how TRIM46 mediates the degradation of YAP1 protein, YAP1 and TRIM46 were ectopically expressed in HEK293T cells, and Co‐IP assays were performed. Notably, TRIM46 was detectable in the anti‐YAP1 immunoprecipitates and vice versa (Figure 8B,C). In addition, MG132, but not CQ or BafA1, rescued the turnover of YAP1 by TRIM46, indicating that TRIM46 mediates YAP1 degradation via the proteasomal pathway (Figure 8D). To determine whether TRIM46 directly regulates YAP1 protein stability, we performed cycloheximide (CHX) chase assays. Immunoblot analysis revealed that YAP1 protein levels declined progressively over time in the presence of TRIM46 overexpression, whereas YAP1 level remained relatively stable in control cells (Figure 8E,F). Since K48 Ubiquitination was responsible for proteasomal protein degradation, we further examined the ubiquitination of YAP1. TRIM46 greatly promoted K48 linked ubiquitination of YAP1, but not K63 ubiquitination (Figure 8G,H). We examined the endogenous K48‐linked ubiquitination and degradation of YAP1 in mouse BMDMs. Our results demonstrated that the genetic knockdown of *Trim46* significantly reduces YAP1 K48‐linked ubiquitination and degradation (Figure 8I–K). To further demonstrate that TRIM46 functions as an E3 ubiquitin ligase for YAP1, we generated truncated TRIM46 and performed in vitro and in vivo ubiquitination assays (Figure 8L,M). Notably, when TRIM46‐ΔRING was substituted for wild‐type TRIM46, YAP1 K48‐linked ubiquitination was abolished, indicating that TRIM46 mediates this modification through its RING domain‐dependent E3 ligase activity (Figure 8L). Additionally, in vivo experiments in HEK293T cells demonstrated that the Really Interesting New Gene (RING) domain of TRIM46 mediates K48‐linked ubiquitination of YAP1 (Figure 8M).

**FIGURE 9:**
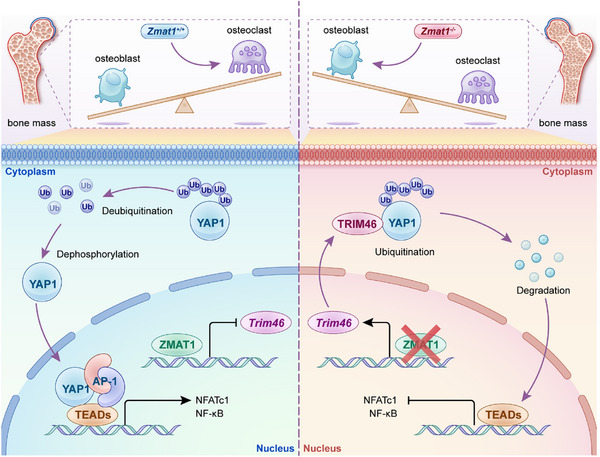
A schematic of *Zmat1* in the regulation of osteoclastogenesis via TRIM46/YAP1 signaling. Normal bone remodeling balance is determined by osteoclast‐induced bone resorption and osteoblast‐mediated bone formation. *Zmat1* deficiency lose the transcriptional inhibition to E3‐ligase TRIM46, which interacts with YAP1 and induces its degradation. This reduces YAP1 translocating to nuclear, which is essential for transcription of key genes in osteoclast differentiation, including NFATc1 and NF‐κB. In contrast, during osteoclast differentiation, *Zmat1* transcriptionally inhibits TRIM46, reduces ubiquitination of YAP1. Therefore, YAP1 enters the nucleus for subsequent activation of osteoclast differentiation related genes. Together, these results indicate that *Zmat1* deficiency alleviates pathological bone loss by activating TRIM46/YAP1 signaling in osteoclasts.

Immunofluorescence analysis of BMDMs demonstrated the co‐localization of YAP1 and TRIM46 (Figure 8N). Finally, we analyzed alterations in the nuclear‐to‐cytoplasmic ratio of YAP1 using immunofluorescence assays. The results demonstrated that RANKL stimulation significantly increases the nuclear‐to‐cytoplasmic ratio of YAP1 in BMDMs. However, upon TRIM46 knockdown, nuclear translocation of YAP1 was markedly enhanced, accompanied by an increase in its nuclear‐to‐cytoplasmic ratio (Figure 8O). Collectively, since the effect of YAP1 on osteoblast and osteoclast differentiation has been revealed [32, 43], our results highlight the promising role of *Zmat1* in the dual targeting of osteoclasts and osteoblasts for the treatment of osteoporosis and related skeletal degenerative disorders (Figure 9).

## 3 Discussion

The role of transcription factors in regulating bone remodeling is a critical area of investigation. In this study, we explored the role of zinc finger matrin‐type 1 (*Zmat1*) in bone homeostasis by focusing on its regulatory functions in osteoclastogenesis and osteoblastogenesis. Our results indicated that during osteoclastogenesis, ZMAT1 acts as a repressor of TRIM46, which in turn regulates the degradation of YAP1 via K48‐linked ubiquitination. *Zmat1* deficiency leads to increased *Trim46* expression and reduced osteoclast activity. In addition, the activity of *Zmat1*‐deficient osteoblasts was enhanced. This highlights the bidirectional regulatory role of ZMAT1 in both osteoclasts and osteoblasts and underscores its complexity and versatility as a transcription factor.

YAP1 is expressed in the osteoblast lineage, which includes committed osteoblast precursors or progenitors, matrix‐producing osteoblast, lining cells, and matrix‐embedded osteocytes [31]. c‐Src/Yes kinases regulate the recruitment of *Runx2* by YAP1 [44]. Additionally, YAP1 stimulate the collagen cross‐linking activity of Loxl2, thereby controlling the differentiation of osteoblasts [45]. In osteoblasts, the expression of type II and IX collagens is dependent on YAP1. These collagen isoforms also regulate osteoclast differentiation [34, 46]. Ubiquitination‐dependent proteasomal degradation of YAP1 is a key regulatory event that controls the level of YAP1 retained in the cytoplasm [47]. YAP1 can be ubiquitinated by E3 ubiquitin ligases that possess the RING domain [48]. This is closely related to the E3 ubiquitin ligases activity. TRIM family members have been shown to play important roles in bone remodeling. *Trim16* and *Trim33* promote osteoblast differentiation [49, 50], while *Trim44* promotes osteoclast differentiation [51]. Additionally, both *Trim38* and *Trim21* have been shown to have bidirectional regulatory effects on the differentiation of osteoblasts and osteoclasts [43, 52].

Notably, YAP1 exhibits a biphasic pattern at protein levels, which does not strictly mirror TRIM46 expression. The non‐linear YAP1 response likely reflects integrated post‐transcriptional and post‐translational regulation of YAP1, rather than a simple linear relationship with TRIM46 alone. Since the rapid loss of YAP1 occurs during the early stage (Day 1) when *Zmat1* expression is low (Figure 7G), other E3 ligases distinct from TRIM46 may drive this initial degradation. In contrast, during the late stage (Day 5), *Trim46* levels are significantly elevated in *Zmat1*‐deficient mice, coinciding with a failure in YAP1 recovery. This suggests that while other mechanisms initiate YAP1 loss, the ZMAT1‐mediated suppression of TRIM46 is the critical determinant for the subsequent re‐expression and maintenance of YAP1.

Our study identifies the ZMAT1‐TRIM46‐YAP1 axis as a previously unknown mechanism that promotes osteoclastogenesis and bone resorption. This finding is innovative in two major aspects: it uncovers ZMAT1 as a novel transcription factor involved in bone metabolism, distinct from the well‐studied regulators of osteoclast differentiation such as NFATc1 and c‐Fos, and reveals a new ubiquitination‐dependent mechanism by which TRIM46 destabilizes YAP1, thereby functionally linking the Hippo signaling pathway to bone homeostasis. Importantly, the dysregulation of this pathway leads to excessive osteoclast activity and pathological bone loss, highlighting its relevance to osteoporosis. In addition, we found that *Zmat1* suppresses osteoblast differentiation. Notably, *Zmat1* exerts a dual regulatory role by promoting osteoclast and inhibiting osteoblast differentiation, thereby providing a novel therapeutic target for the treatment of bone‐related diseases. Given that current osteoporosis therapies primarily target RANKL signaling or bone resorption in a non‐specific manner, our findings provide a potential new therapeutic avenue by targeting ZMAT1 or TRIM46 to restore the balance between bone formation and resorption.

The ZMAT1‐TRIM46‐YAP1 axis represents a novel regulatory module that may functionally interact with canonical pathways involved in bone remodeling. For instance, RANK/RANKL/OPG signaling is indispensable for osteoclast differentiation and bone resorption [53]. It is plausible that ZMAT1 enhances RANKL‐induced osteoclastogenesis by altering YAP1 expression, which in turn facilitates downstream NF‐κB and NFATc1 activation. Similarly, Wnt/β‐catenin signaling, a key regulator of osteoblast activity and bone formation [54], may antagonize the ZMAT1‐driven catabolic effect, suggesting a layered regulatory balance between anabolic and catabolic processes. Furthermore, since YAP1 is a central effector of the Hippo signaling pathway, the ZMAT1–TRIM46–YAP1 axis could act as a convergence point, modulating Hippo signaling and influencing osteoblast–osteoclast coupling. Together, these insights indicate that ZMAT1‐mediated regulation is not an isolated event, but rather integrates into the hierarchical and synergistic network of signaling pathways that maintain bone homeostasis. Although our study elucidates the ZMAT1/TRIM46/YAP1 axis, several limitations warrant further investigation. However, the detailed mechanism by which YAP1 undergoes proteasome‐mediated degradation during the early phase of osteoclastogenesis remains unclear. Moreover, the in vivo relevance of *Zmat1* in human bone homeostasis and its potential as a therapeutic target for osteoporosis requires further validation in clinical settings. Future research should explore the detailed molecular mechanisms underlying ZMAT1's dual regulatory roles, including its interactions with other transcription factors and signaling pathways.

In conclusion, our findings highlight the significance of *Zmat1* in bone metabolism and its potential as a dual therapeutic target for modulating osteoclast and osteoblast activities. These insights will pave the way for the development of novel strategies for the treatment of osteoporosis and other skeletal disorders by targeting the ZMAT1/TRIM46/YAP1 axis.

## 4 Materials and Methods

### 4.1 Study Approval

The human studies were approved by the Shanghai Changhai Hospital Medical Ethics Committee (No. CHEC2025‐488). PBMCs were isolated from blood samples obtained from healthy donors and patients diagnosed with osteoporosis. Written informed consent was obtained from all the participants before sample collection. No additional interventions were performed for the purpose of this study. All in vivo animal experiment and protocols were approved by The Navy Medical University and The Shanghai Changhai Hospital Medical Ethics Committee (No. CHEN (A.E)2025‐048). All animal procedures complied with the institutional guidelines and national regulations for laboratory animal welfare.

### 4.2 Establishment of Global *Zmat1* Knockout Mice

*Zmat1* knockout mice were generated using a C57BL/6 genetic background. Heterozygous mice were intercrossed over three generations to obtain the stable genotypes of *Zmat1^+/+^* and *Zmat1^−/−^* mice. The *Zmat1^+/+^* and *Zmat1^−/−^* experimental mice were the offspring of the heterozygotes. Tail genomic DNA PCR was performed to identify the mice with the expected genotype using the following primers: 5′‐TATTTATTACAAACAGAGGCACTACAGCA‐3′ and 5′‐GCACCTTATCAGGGACTACACCA‐3′ for wild type; 5′‐GTAGATAAACTAATAAACAAGGGCAACA‐3′ and 5′‐TTCCTCAAATACGCCATTCAAA‐3′ for *Zmat1* knockout (Figure S3B). Three‐month‐old mice were subjected to OVX and euthanized six months later.

### 4.3 Cell Culture and Differentiation

The RAW264.7 cell line was obtained from the China Infrastructure of Cell Line Resources (Beijing, China). For osteoclastic differentiation, bone marrow‐derived macrophages (BMDMs) were derived from 4‐to‐8‐week‐old *Zmat1^+/+^* and *Zmat1^−/−^* mice, and then incubated in MEM‐α containing 50 ng/mL M‐CSF for 4 days to generate BMDMs. To generate mature osteoclasts, BMDMs were cultured with 50 ng/mL M‐CSF and 100 ng/mL RANKL (Peprotech) for at least 4 days. RAW264.7 monocytes/macrophages (ATCC) were cultured and maintained in MEM‐II supplemented with 10% FBS (Gibco, USA) and 1% penicillin/streptomycin (Gibco, USA). To induce the differentiation of RAW264.7 cells into osteoclasts, 100 ng/mL RANKL was added to the cells for three more days, and the medium was changed every 2 days. PBMCs were isolated from EDTA‐anticoagulated blood samples obtained from healthy donors and osteoporotic (OP) patients using Ficoll density gradient centrifugation (Lymphoflot, Bio‐Rad). The cells were seeded at a density of 3 × 10^6^ cells/mL in adhesion medium composed of α‐MEM supplemented with 10% fetal calf serum (FCS) and 1% penicillin/streptomycin (PS), followed by incubation at 37°C in a humidified atmosphere containing 5% CO_2_ for 1.5 h to allow monocyte adherence. Non‐adherent cells were removed along with the supernatant, and the adherent monocytes were washed gently with PBS. Subsequently, the cells were cultured in α‐MEM containing 50 ng/mL macrophage colony‐stimulating factor (M‐CSF) and 100 ng/mL receptor activator of nuclear factor κB ligand (RANKL) to induce osteoclast differentiation. For osteogenic differentiation, BMSCs were reseeded at a density of 5 × 10^5^ cells/well in 6‐well plates. BMSCs were then stimulated with Osteogenic Stimulatory Medium (mouse) (Cayen, China). The osteogenic medium was replaced every three days for all cells.

### 4.4 TRAP Staining Assay

TRAP staining was performed after mature osteoclasts were successfully generated in the control group. Cells were fixed with 4% paraformaldehyde at room temperature for 30 min, rinsed three times with PBS (5 min each), and then incubated with TRAP staining solution at 37°C in the dark for 1 h before observation. The specific steps of TRAP staining were performed according to the manufacturer's instructions (Sigma‐Aldrich, 387 A, Germany). TRAP‐positive cells were visualized, and the number of osteoclasts (OCs) per field of view and the number of nuclei per TRAP^+^ cell were quantified.

### 4.5 Actin Ring Formation Assay

BMDMs were treated with 100 ng/mL RANKL for four days. The BMDMs and the mature osteoclasts were fixed with 4% formaldehyde, permeabilized with 0.1% Triton X‐100, and incubated with β‐actin Polyclonal Antibody (Invitrogen, PA1‐183, USA) for 20 min. Finally, the nuclei were stained with DAPI for 2 min. After washing with phosphate‐buffered saline (PBS), the cells were photographed under a fluorescence microscopy.

### 4.6 Bone Resorption Assay

BMDMs were treated with 100 ng/mL RANKL for four days. Finally, BMDMs and mature osteoclasts were completely removed from the dentin discs and stained with hematoxylin. The resorption pits were visualized under a light microscope, measured, and presented as relative pit area (%).

### 4.7 Alizarin Red and ALP Staining

For alizarin red staining, osteogenic BMSCs were collected at 14 days, fixed in 4% paraformaldehyde for 15 min, and stained with 40 mM alizarin red (pH 4.9, Sigma) for 15 min. For ALP staining, the osteoblasts were rinsed with PBS and fixed with 4% paraformaldehyde at room temperature for 20 min. After three washes with PBS, the samples were completely covered with an ALP staining working solution prepared according to the instructions provided with the ALP staining kit (Beyotime, China). The samples were incubated in the dark for 20 min at ambient temperature. After incubation, the staining solution was removed, and the cells were rinsed 1–2 times with distilled water to stop the color reaction. A microscopic examination was then performed.

### 4.8 Plasmid and Lentivirus Construction

Plasmid construction and lentivirus transfection were performed using ViraPower Promoterless Lentiviral Gateway Kits (Invitrogen, USA) according to the manufacturer's instructions.

### 4.9 Small Interfering RNA

siRNA targeting *Zmat1* and *Trim46* were purchased from GenePharma (Shanghai, China). The sequences are listed in Table S2. siRNA transfection was performed using Lipofectamine 2000 (Thermo Fisher Scientific, Cat# 11668019, USA) as described in our previous study. Briefly, RAW264.7 cell lines were seeded in 6‐well plates at a density of 2 × 10^5^ cells per well for 24 h and then transiently transfected with 5 µL of Lipofectamine 2000 and 5 µL of siCon, si*Zmat1* or si*Trim46* for 4 h. Then, the medium was changed to complete α‐MEM, and the knockdown effect of *Zmat1* or *Trim46* was verified by quantitative qPCR and immunoblotting after 48 h.

### 4.10 RNA Isolation and qPCR

Total RNA was isolated from the cells using TRIzol reagent (Invitrogen). Whole cells were collected in 1 mL of TRIzol reagent and homogenized on ice using a tissue homogenizer (PRO). cDNAs were synthesized using a reverse transcription kit (Takara, Japan), and qPCR was performed on a ViiA 7 Real‐Time PCR System (Applied Biosystems) using SYBR (Takara). *β‐actin* was used as an internal control for cDNA. Primers used in this study are listed in Table S1.

### 4.11 Protein Concentration and Immunoblotting Assay

Cells were lysed in RIPA lysis buffer (50 mM Tris‐HCl, pH 7.4, 150 mM NaCl, 1% Nonidet P‐40, and 0.1% SDS). Protein fractions were centrifuged at 13 000 rpm at 4°C for 15 min. Subsequently, supernatants were collected, subjected to sodium dodecyl sulfate‐polyacrylamide gel electrophoresis, and transferred onto NC membranes. The membranes were blocked with 5% BSA and incubated with specific antibodies overnight at 4°C. Primary antibodies used in this study are listed in Table S3. Horseradish peroxidase‐conjugated secondary antibodies (Cell Signaling Technology, USA) were used at a dilution of 1:5000. Antibody complexes were visualized using an enhanced chemiluminescence detection system (Millipore, Burlington, MA, USA).

### 4.12 Co‐Immunoprecipitation (Co‐IP) and Ubiquitination Assay

For standard Co‐IP assays to examine protein interactions, whole‐cell extracts were prepared using lysis buffer after transfection or stimulation and incubated with the appropriate antibodies overnight at 4C. Protein A&G beads (Abmart) were added, and incubation continued for 4 h at 4C. Coprecipitated proteins were washed, eluted with SDS‐loading buffer at 95C for 5 min, and subjected to western blotting analysis. For ubiquitination analysis, a denatured immunoprecipitation (dIP) strategy was employed to eliminate interference from ubiquitinated binding partners. Briefly, cells were harvested in denaturing lysis buffer containing 1% SDS and boiled at 95C for 10 min to disrupt non‐covalent protein interactions. The lysates were then diluted tenfold with standard lysis buffer (without SDS) to reduce the SDS concentration to 0.1% before incubation with primary antibodies and Protein A&G beads as described above.

### 4.13 Inhibitor Experiments

Cells were seeded in six‐well plates at a density of 1.2 × 10^6^ cells/well, with four replicates. Bone marrow‐derived macrophages were treated with a SuperTDU inhibitor to induce osteoclast differentiation, and proteins were extracted after 5 days of RANKL stimulation for subsequent experiments. HEK293T cells were treated with the inhibitors CQ, Baf A1, MG132, and CHX. The medium containing the inhibitors was replaced every other day. DMSO‐treated cells were used as controls.

### 4.14 Immunofluorescence (IF) Staining

OCs or OBs induced as described above were then fixed with 4% PFA for 20 min, permeabilized with 0.1% (v/v) Triton X‐100 for 10 min, and blocked with 5% skim milk for 1 h. Cells were then incubated with primary antibodies (YAP1, CST, Cat# 14074, USA, 1:50; TRIM46, CST, Cat#92574T, USA, 1:200) at 4°C overnight, washed with phosphate‐buffered saline with Tween 20 (PBST) 3 times for 5 min, and then incubated with a fluorescent secondary anti‐mouse antibody (Alexa Fluor 488, green, CST, Cat#8878, USA, 1:100; Alexa Fluor 594, red, CST, Cat#8890S, USA, 1:100). Images were captured using a laser‐scanning confocal microscope (Zeiss LSM 880, Germany).

### 4.15 Ovariectomy‐Induced Bone Loss

Ten‐week‐old female mice were ovariectomized or sham‐operated. Ovariectomized mice were treated with an intravenous injection of Usp26 overexpression adenovirus or control adenovirus starting 2 days after ovariectomy. All mice were euthanized after 2 months, and three mice from each group were randomly assigned for MSCs and BMDMs isolation and subsequently for osteoblastic differentiation and osteoclastic differentiation, respectively. Samples from the remaining 7 mice in each group were collected for micro‐CT scanning and histological analysis.

### 4.16 Cranial Cortical Defect Surgery

Surgery for cranial cortical bone defects was performed as follows. 8‐week‐old *Zmat1^−/−^* mice and littermate controls were anesthetized using an intraperitoneal injection of pentobarbital sodium. An incision was made along the midline of the mouse head to expose the skull. Drill defects in the mouse skull were created using a circular drill bit with a diameter of approximately 2.5 mm. Samples were collected for micro‐computed tomography (CT) scanning and histological analysis 2 weeks after surgery.

### 4.17 Bone Marrow Reconstitution

Bone marrow‐derived cells were obtained by flushing the tibiae and femora of donor mice. Recipient CD45.1 mice were irradiated with 9 Gy and reconstituted by intravenous injection with an equal ratio of BM cells from donor mice (CD45.2 *Zmat1^+/+^* or *Zmat1^−/−^*). The mice were used for at least 8 weeks after bone marrow reconstitution.

### 4.18 Micro‐Computed Tomography

Micro‐CT analysis was performed on the left femur of each mouse as described previously. After fixation with 4% paraformaldehyde, the femurs were scanned on a Skyscan 1172 (Aartselaar, Belgium) with a 10 µm isotropic voxel size, 50 keV, 500 µA. Regions of interest (ROIs) were defined for trabecular and cortical parameters. The trabecular ROI extended from 1 mm proximally to the end of the distal growth plate and over 1 mm toward the diaphysis. The cortical ROI extended from 3 mm proximally to the end of the distal growth plate and over 1 mm toward the diaphysis. The resulting 2D images of the trabecular and cortical bones in relative cross‐sections are shown in grayscale. The bone parameters included trabecular number (Tb.N, 1/mm), bone volume per tissue volume (BV/TV, %), bone mineral density (BMD; g/cm^3^), trabecular separation (Tb.Sp, mm), trabecular pattern factor (Tb.Pf, mm), BS/BV ratio (1/mm), trabecular thickness (Tb.Th, mm), and trabecular separation (Tb.Sp, mm).

For the analysis of cortical bone regeneration, the volume of interest (VOI) was defined as the cylindrical area covering the initial bone defect. The BV/TV and BMD were calculated within the delimited VOI.

### 4.19 Histology H&E and TRAP

Right femurs were isolated, fixed in 4% paraformaldehyde for 24 h, decalcified in 12.5% ethylenediaminetetraacetic acid (EDTA; pH 7.0) for 3 weeks, and embedded in paraffin. Longitudinal sections with a thickness of 8 µm were cut and stained with hematoxylin and eosin (H&E), alkaline phosphatase (Beyotime, China), and TRAP (Sigma, USA), in accordance with the manufacturer's protocols. Bone histomorphometric analysis of OC. S/BS, and TB. S/BS was performed using ImageJ software, and all images were collected using fluorescence microscopy.

### 4.20 The Whole Transcriptome Analysis

To identify the transcription factors specifically involved in RANKL‐induced osteoclast differentiation, we first isolated bone marrow‐derived monocyte/macrophage precursor cells (BMDMs) from wild‐type mice and subjected them to differential stimulation with lipopolysaccharide (LPS; 100 ng/mL) or RANKL (100 ng/mL) for 4 h and 72 h, respectively, to model inflammatory activation and osteoclastogenesis induction. Total RNA was extracted at each time point and subjected to transcriptomic analysis by RNA sequencing (RNA‐seq). To distinguish RANKL‐specific transcriptional changes, we performed comparative expression profiling and filtered out genes that were similarly regulated by LPS and RANKL stimulation. Only genes that exhibited significant differential expression in response to RANKL, but not to LPS, were retained for further analysis. Raw data were submitted to the NCBI BioProject database under the accession number GSE274540. In the study of the mechanisms by which *Zmat1* regulates osteoclasts, total RNA was extracted from BMDMs isolated from *Zmat1^+^/^+^* and littermate *Zmat1^−^/^−^* mice following RANKL stimulation for 1 and 3 days and subjected to transcriptomic analysis. Raw data were submitted to the NCBI BioProject database under the accession number GSE274930. RNA extraction, mRNA library construction, and sequencing were performed as described previously. After the final transcriptome was generated, StringTie and Ballgown were used to estimate the expression levels of all transcripts and genes by calculating the FPKM (FPKM=[total_exon_fragments/mapped_reads(million)×exon_length(kB)]). Differentially expressed transcripts and genes with a fold change > 1.5 or fold change < 0.66 and P value < 0.05 were selected using the R package edgeR. The signaling pathways correlated with differentially expressed mRNAs were enriched using KEGG pathway analysis.

### 4.21 CUT&Tag and Sequencing (Cleavage under Targets & Tagmentation)

The CUT&Tag assay was performed using the Hyperactive Universal CUT&Tag Assay Kit for Illumina (Vazyme, Shanghai, China), according to the manufacturer's instructions. Briefly, RAW264.7 were stimulated with RANKL and then harvested after washing with PBS. Cells (1 × 10^5^) were bound with concanavalin A‐coated magnetic beads and then incubated with an antibody against at 4°C overnight. The next day, samples were incubated with secondary antibody and then with Hyperactive pA/G‐Transposon for 1 h. DNA was treated with TTBL at 37°C for 1 h and extracted, followed by purification for library amplification. Next‐generation sequencing (second‐generation high‐throughput sequencing) was used to sequence the DNA. Genome mapping was performed using Bowtie (version 0.12.8), and clean reads were compared to the reference genome mm10. MACS (version 2.2.7.1) was used to call the peaks. The BAM file and R package DiffBind were used to analyze the differences between the peaks of the two specified samples. The default parameter was set as fold change ≥ 2 or ≤ 1/2 and P value < 0.05, and the differences between the obtained peaks were annotated. The annotation program was the same as the aforementioned peak annotation program, which included different peak locations and annotation information. The motif analysis was conducted using the “HOMER” tool. The HOMER motif was predicted based on the existing database within the peak. The findmotifs Genome.pl tool for HOMER was used to analyze the specific location of the motif at the peak.

### 4.22 Chromatin Immunoprecipitation (ChIP)

BMDMs were first stimulated with RANKL for the indicated times, cross‐linked with 1% (v/v) methanol‐free formaldehyde for 10 min, terminated with a glycine solution, and then subjected to ChIP assays using a ChIP Assay Kit (Beyotime, China). The antibodies against *Zmat1* (2 µL/sample) and RNA Pol II (1 µL/sample) were from Bethyl Laboratories and Millipore respectively. Purified DNA was detected using qPCR and normalized to the input DNA for each sample. The sequences are presented in Table S1.

### 4.23 Statistical Analysis

All experimental procedures and quantitative assessments were performed in a blinded manner. Assumptions of normality and homoscedasticity were checked for each dataset before the parametric analyses. Data were presented as mean ± SD and *p* < 0.05 was considered statistically significant, with the sample size (n) for each analysis set to be ≥ 3. To evaluate significant differences between groups, Student's t‐test was used for comparisons between two groups, while one‐way or two‐way analysis of variance (ANOVA) was applied for multiple group comparisons. In the presence of significant ANOVA results, post‐hoc analyses were conducted using Tukey's test to adjust for multiple comparisons. All statistical analyses were performed using Prism 6.0 (GraphPad Software, La Jolla, CA, USA).

## Author Contributions

X.C., Y.H., and L.C. contributed equally to this study. S.X., S.X., and H.Z. designed the research; X.C. and Y.H. performed the research; L.C., H.Y., J.C., R.L., and J.S. contributed new reagents; M.C., C.D., Z.Z., and L.L. writing – review and edited.

## Conflicts of Interest

The authors declare no conflicts of interest.

## Supporting information




**Supporting File 1**: advs74602‐sup‐0001‐SuppMat.docx.

**Supporting File 2**: advs74602‐sup‐0002‐SourceData.docx.

## Data Availability

The data that support the findings of this study are available from the corresponding author upon reasonable request.
