# Supplementary material for: ZMAT1 Promotes Osteoclastogenesis Through TRIM46 Mediated YAP1 Degradation and Inhibits Osteoblastogenesis
Source: Adv Sci (Weinh). 2026 Mar 2;13(27):e21783. doi: 10.1002/advs.202521783 (PMC13170258; doi:10.1002/advs.202521783)
Supplement: Supplementary file 1 — Supporting File 1: advs74602‐sup‐0001‐SuppMat.docx. [file ADVS-13-e21783-s002.docx]

Title: **ZMAT1 Promotes Osteoclastogenesis through TRIM46 Mediated YAP1 Degradation and Inhibits Osteoblastogenesis**

Author names: Xinyu Chang^1^, Yijin Hou^1^, Likun Cui^2^, Huiqi Yu^1^, Rong Liu^1^, Junhao Sui^1^, Zhong Zheng^1^, Lu Liu^1^, Jie Chen^2^, Mengchen Chen^1^, Chen Ding^1^, Shuogui Xu^1^*, Sheng Xu^2^*, Hao Zhang^1^*.

X. Chang, Y. Hou, and L. Cui contributed equally to this article.

Affiliations:

1Department of Traumatic Orthopedics, Changhai Hospital, Second Military Medical University, Shanghai, China,

2National Key Laboratory of Medical Immunology and Institute of Immunology, Institute of Immunology, Second Military Medical University, Shanghai, China,


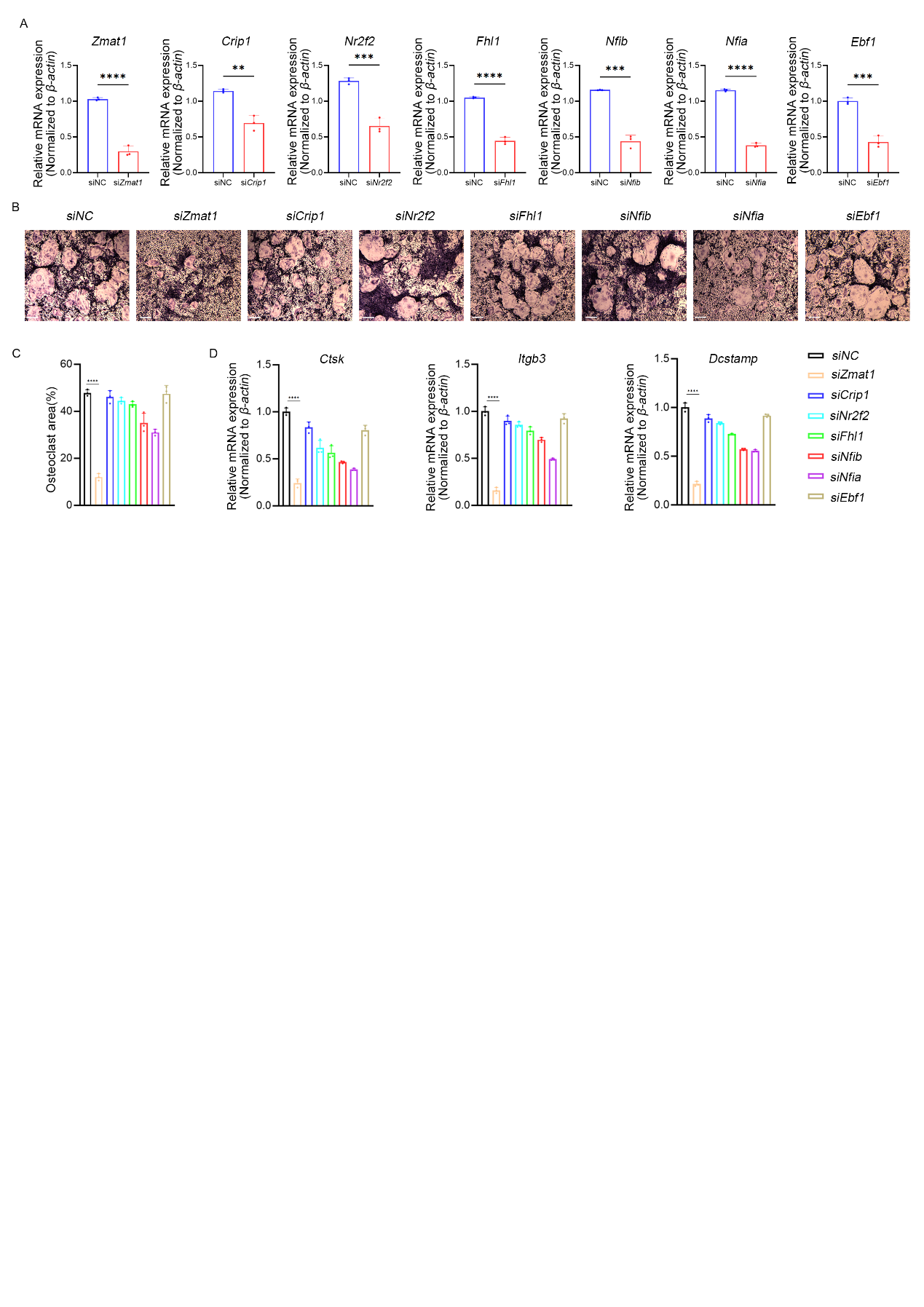


**Supplementary Figure 1. Screening of transcription factors during osteoclast differentiation and identification of *Zmat1* as a potential regulator.** **A** The efficiency of siRNA-mediated knockdown of seven transcription factors (*Zmat1*, *Crip1*, *Nr2f2*, *Fhl1*, *Nfib*, *Nfia*, and *Ebf1*) was validated by qPCR in BMDMs. *β-actin* was used as an internal control. **B** Representative TRAP staining images of osteoclasts differentiated from BMDMs transfected with control siRNA (siNC) or each transcription factor–targeting siRNA. Scale bar, 200 μm. **C** Quantification of osteoclast area (%) based on TRAP staining. **D** Relative mRNA expression levels of osteoclast differentiation markers (*Ctsk*, *Itgb3*, and *Dcstamp*) in BMDMs transfected with the indicated siRNAs, normalized to *β-actin*. All bar graphs are presented as the mean ± SD. ****p < 0.0001 by Student’s *t* test and one-way ANOVA.


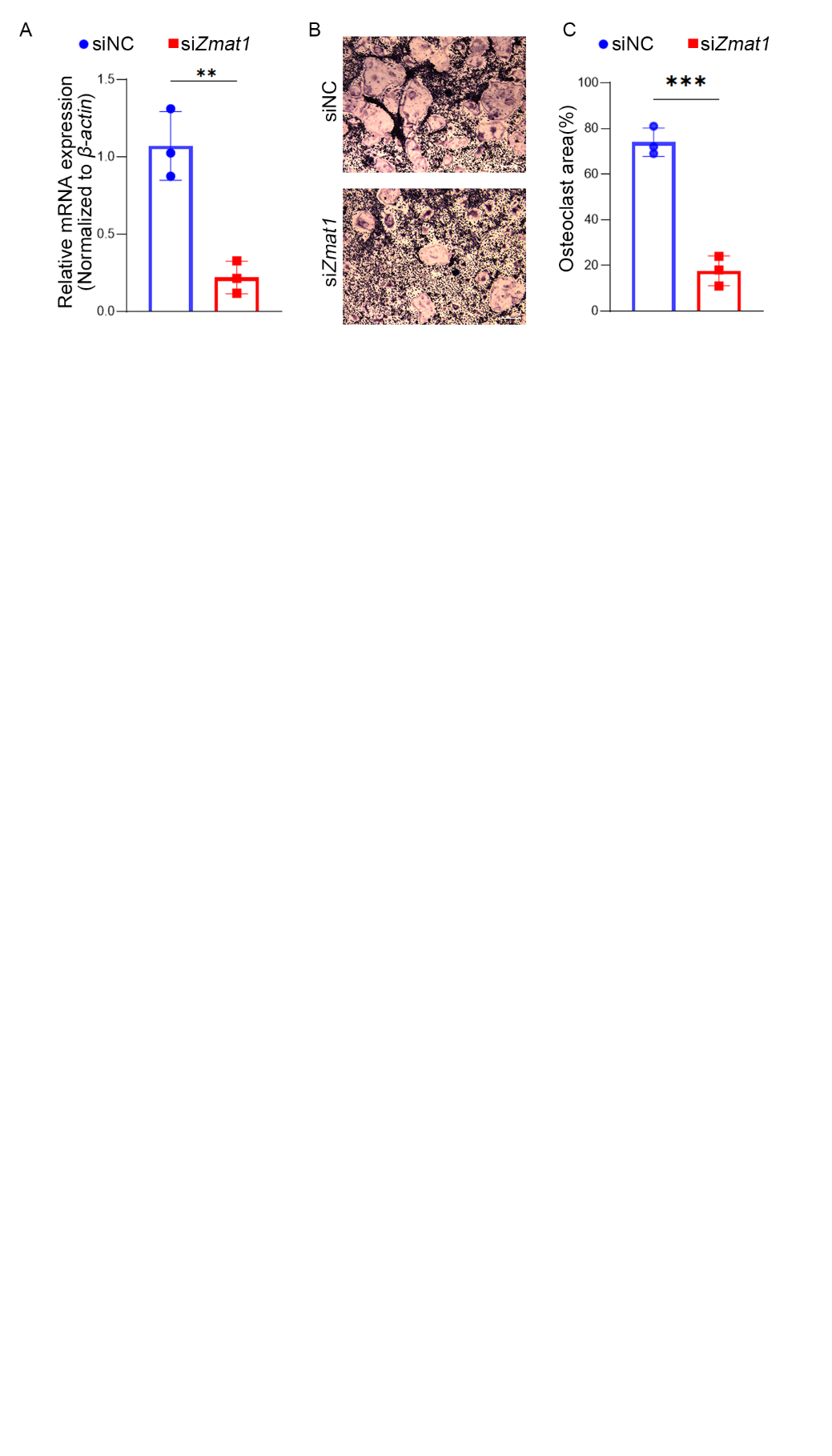


**Supplementary Figure 2. *Zmat1* deficiency reduces osteoclastogenesis*.* A** Knockdown of endogenous *Zmat1* by siRNA. Relative mRNA expression of *Zmat1* in BMDMs from siNC or si*Zmat1* was validated via genotyping by qPCR. **B** Analysis of osteoclast differentiation by TRAP staining. Scale bars represent 100 μm. **C** Quantitative analysis of TRAP staining results for **B**. All bar graphs are presented as the mean ± SD. **p < 0.01; ***p < 0.001; n.s. not significant by Student’s *t* test.


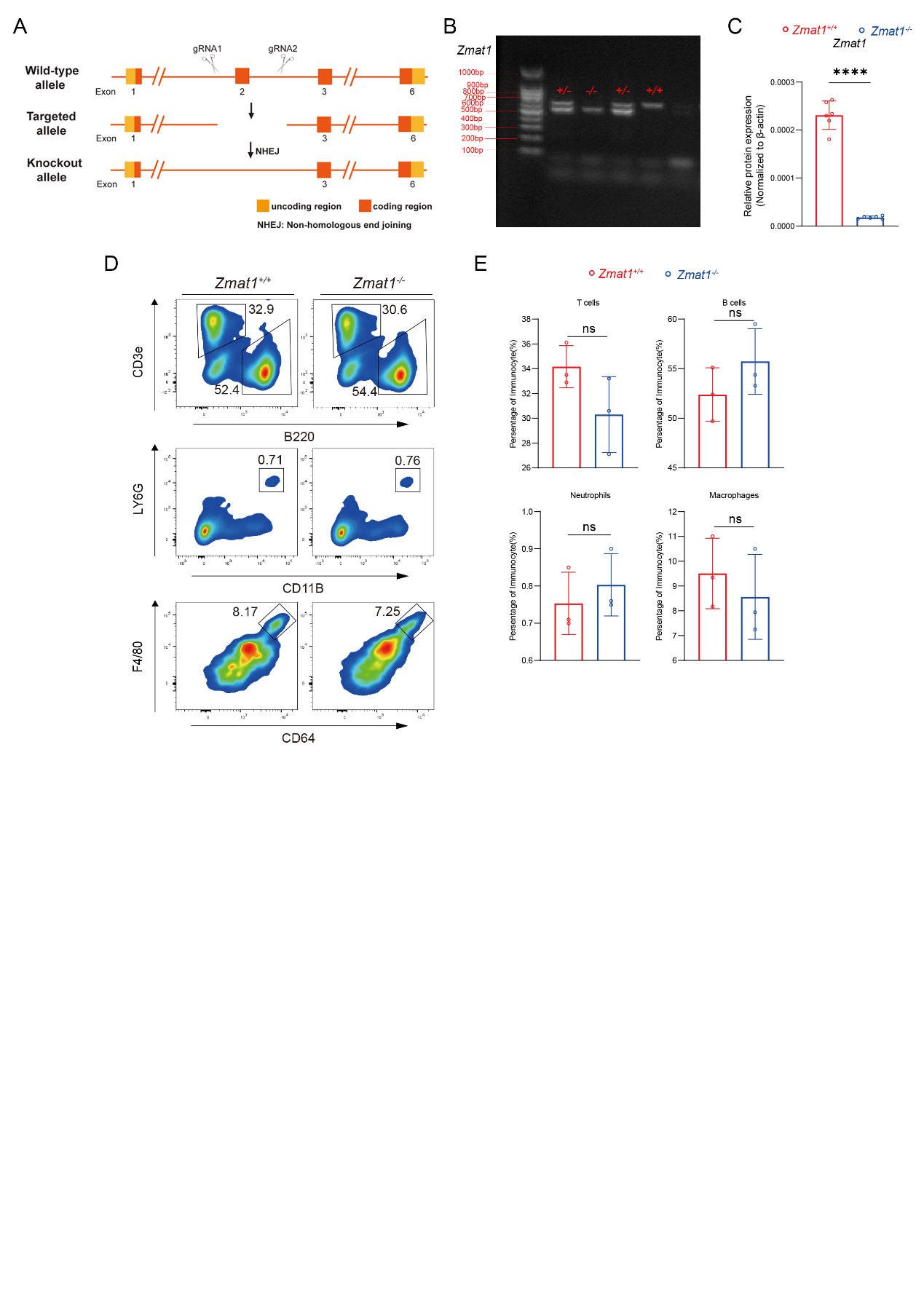


**Supplementary Figure 3. *Zmat1* deficiency does not affect monocyte/macrophage development. A** *Zmat1* global knockout (*Zmat1^-/-^*) mice were designed and generated. **B** Genotype identification via PCR amplification. We performed PCR on genomic DNA extracted from mouse tail tissue using primers specifically designed to distinguish between the wild-type (WT) and knockout alleles. The PCR results demonstrated clear and distinguishable band sizes for *Zmat1^⁺/⁺^* and *Zmat1^⁻/⁻^* mice (corresponding to 675bp and 514bp, respectively), *Zmat1^+/−^* mice exhibited two bands. **C** Relative mRNA expression of *Zmat1* in BMDMs from *Zmat1^+/+^* and *Zmat1^-/-^* mice was validated by qPCR. n=6. **D** Flow cytometry plots showing the gating strategy and frequencies of CD3e+ T cells, B220+ B cells, LY6G+ neutrophils, and F4/80+ CD64+ macrophages in bone marrow cells from *Zmat1^+/+^* and *Zmat1^-/-^* mice. **E** Quantitative analysis of the percentage of T cells, B cells, neutrophils, and macrophages in bone marrow, expressed as mean ± SD (n = 3 mice per group). All bar graphs are presented as the mean ± SD. *p < 0.05; n.s. not significant by Student’s *t* test.


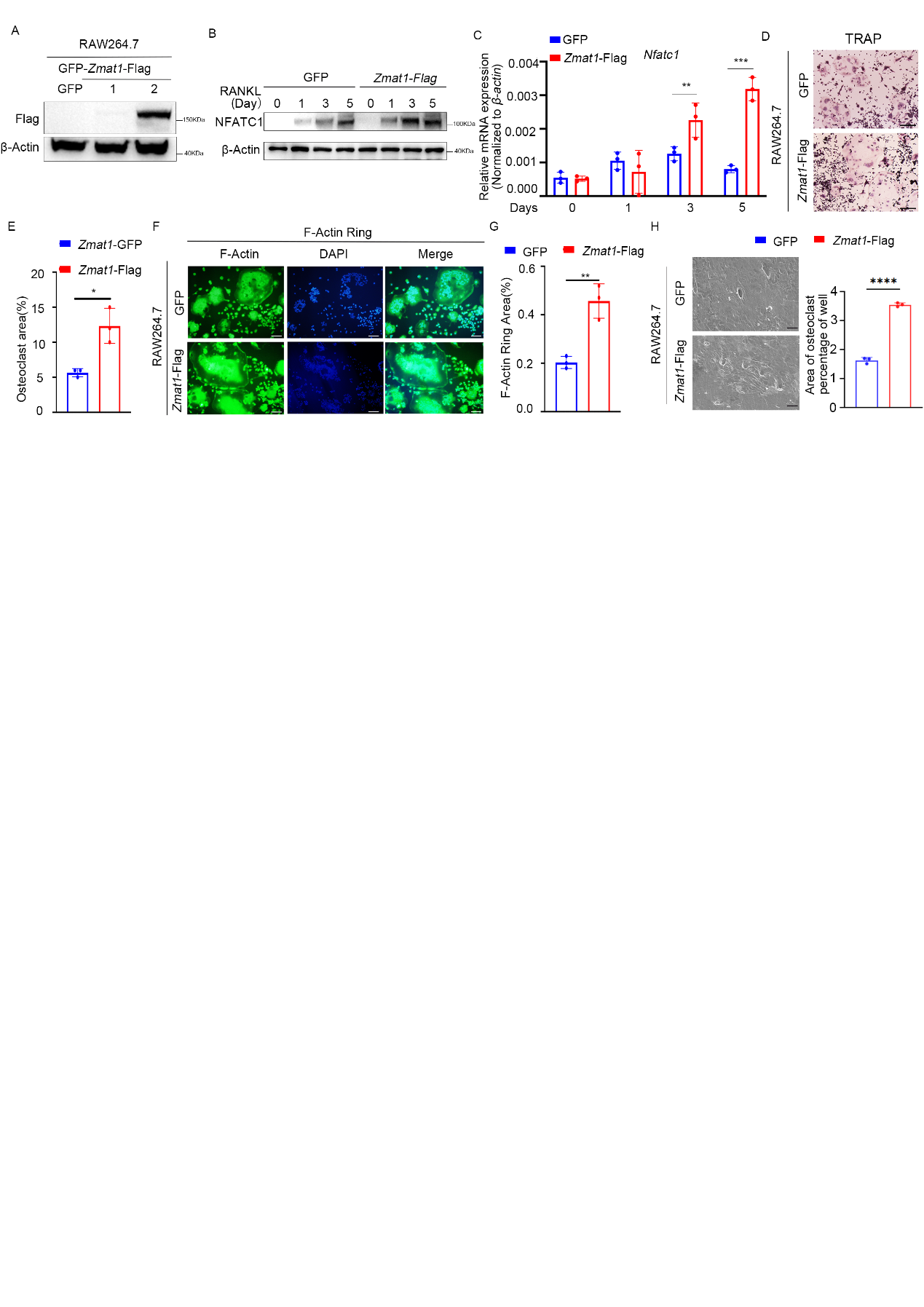


**Supplementary Figure 4. ZMAT1 overexpression promotes osteoclast differentiation**. **A** Overexpressed ZMAT1 was detected by western blot with an anti-Flag antibody. **B** Representative immunoblot of NFATc1 in RAW264.7 cells overexpressing ZMAT1 or the control group. β-Actin was used as a loading control. Osteoclast precursor cells were cultured with M-CSF and RANKL in day0, 1,3 and 5. **C** RT-qPCR analysis of *Nfatc1* mRNA from GFP or *Zmat1*-Flag during osteoclastogenesis at day 0, 1,3 and 5. Normalized relative to *β-actin* mRNA. n = 3. **D, E** Representative TRAP staining of *Zmat1*-overexpressed RAW264.7 and quantitative analysis of TRAP positive staining area are shown. **F** Fluorescence microscopy of F-actin ring stain in mature osteoclasts. Scale bars represent 100 μm. **G** Quantification of F-actin area, expressed as the percentage of total cell area. n = 3. **H** Representative images and quantification of bone resorption pits formed by mature osteoclasts. All bar graphs are presented as the mean ± SD. *p < 0.05; **p < 0.01; ***p < 0.001; n.s. not significant by Student’s *t* test and two-way ANOVA.


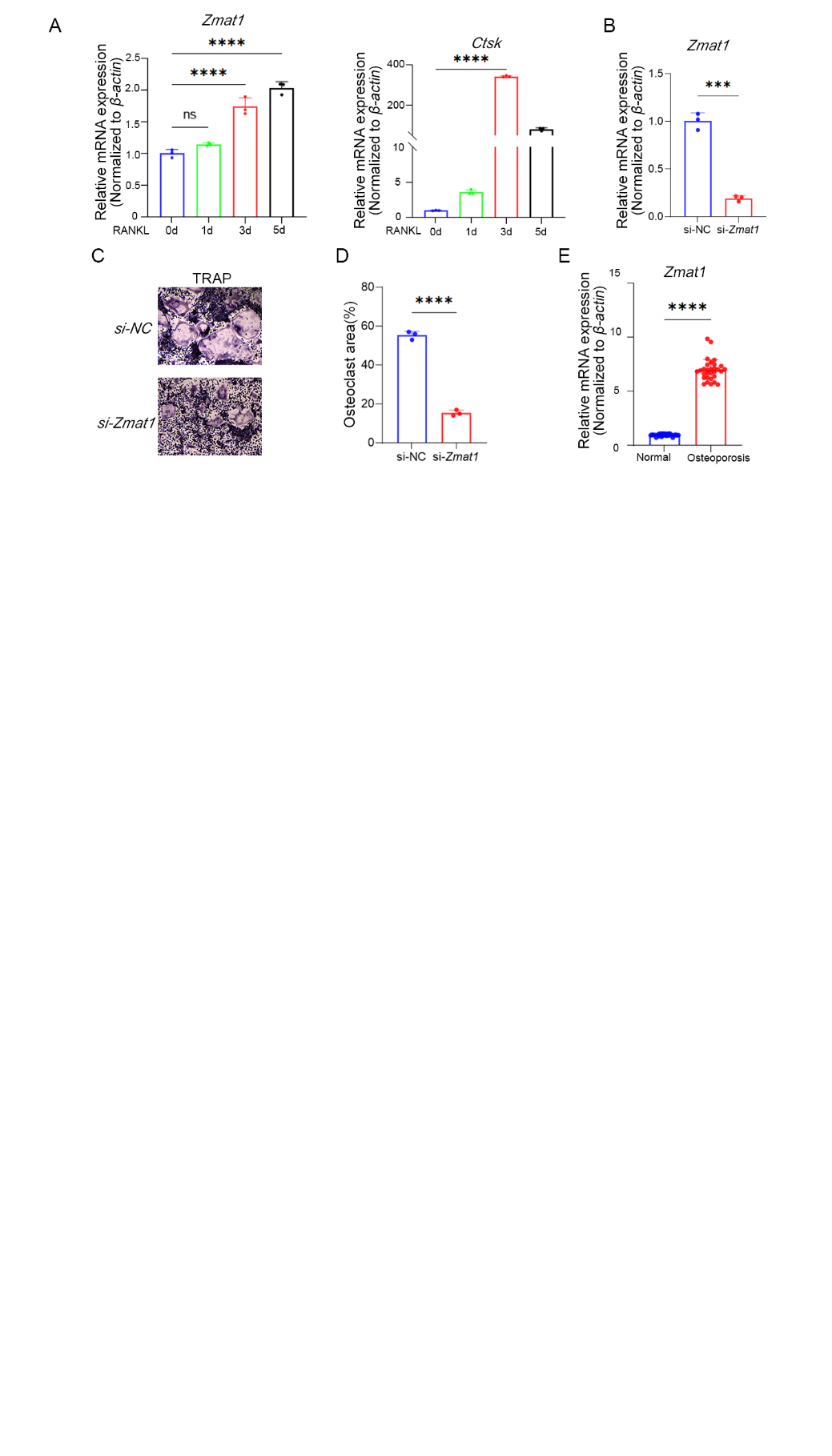


**Supplementary Figure 5. *Zmat1* regulates the differentiation of human osteoclasts in vitro.** **A** *Zmat1* and *Ctsk* mRNA expression in PBMC-derived monocytes undergoing osteoclast differentiation upon RANKL stimulation at days 0, 1, 3, and 5. **B** qPCR analysis showing the relative mRNA expression level of *Zmat1* after siRNA-mediated knockdown during human osteoclast differentiation. n=3. **C** Analysis of human osteoclast differentiation by TRAP staining. Scale bars represent 100 μm. **D** Quantitative analysis of TRAP staining results for **C**. **E** mRNA expression of *Zmat1* in PBMCs from normal and osteoporosis patients (n=30). All bar graphs are presented as the mean ± SD. ***p < 0.001, ****p < 0.0001 by one-way ANOVA.


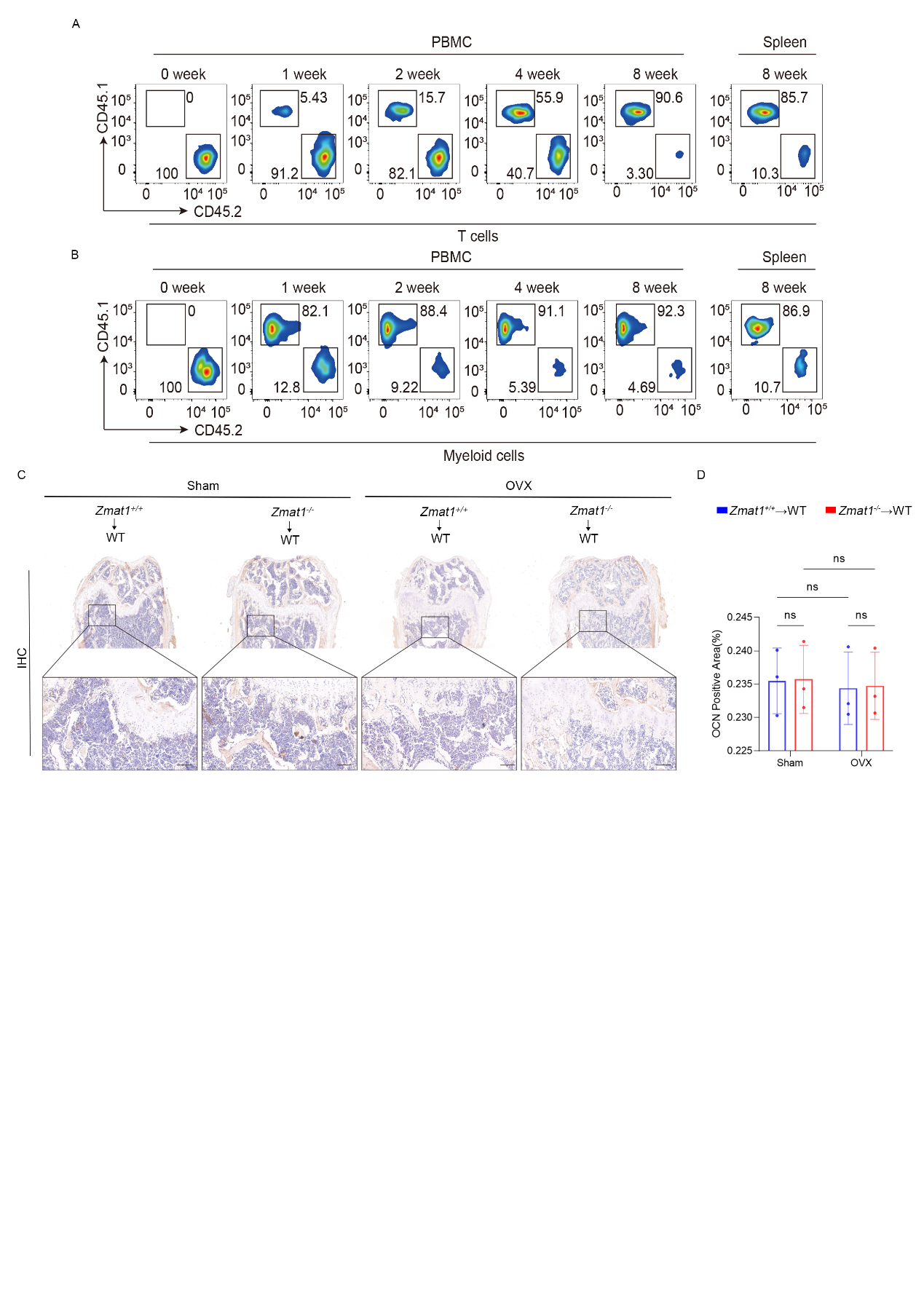


**Supplementary Figure 6. Bone marrow transplantation model validation in *Zmat1^+/+^*/*Zmat1^-/-^* recipient mice.** CD45.1 donor-derived bone marrow cells were transplanted into lethally irradiated (8Gy) CD45.2 *Zmat1^+/+^/Zmat1^-/-^* recipient mice. **A** Peripheral blood T cell percentages were analyzed at 0, 1, 2, 4, and 8weeks post-transplantation to assess reconstitution kinetics. Recipient mice were euthanized at 8 weeks for splenic T cell proportion analysis by CD45.1/CD45.2 staining. **B** Peripheral blood myeloid cell percentages were measured at 0, 1, 2, 4, and 8 weeks post-transplantation as indirect indicators of myeloid cell reconstitution kinetics. Splenic myeloid cell proportions were analyzed upon euthanasia at the 4-week endpoint. **C** Representative immunohistochemical staining of osteocalcin (OCN) in femoral sections from *Zmat1^+/+^* and *Zmat1^−/−^* mice under Sham or ovariectomy (OVX) conditions. Insets show higher-magnification views of the trabecular bone region. Scale bar = 100 μm. **D** Quantification of OCN-positive area (%). Data are presented as mean ± SD (n = 3). n.s. not significant by two-way ANOVA.


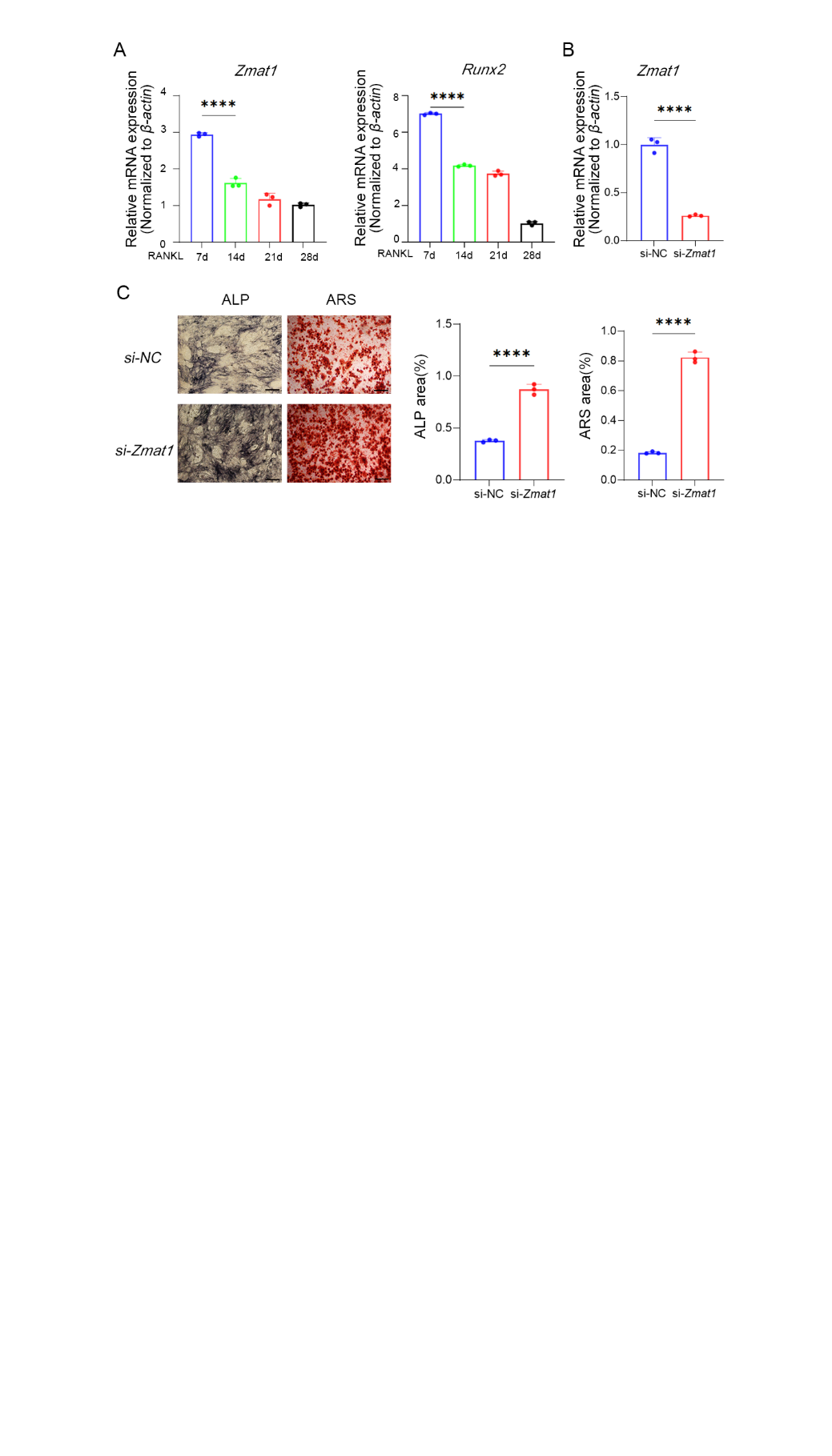


**Supplementary Figure 7. *Zmat1* regulates the differentiation of human osteoblasts in vitro. A** *Zmat1* and *Runx2* mRNA expression in human BMSCs undergoing osteogenic differentiation at days 7, 14, 21, and 28. n=3. **B** qPCR analysis showing the relative mRNA expression level of *Zmat1* after siRNA-mediated knockdown of human BMSCs. **C** Analysis of human osteoblast differentiation by ALP and ARS staining. Scale bars represent 100 μm. All bar graphs are presented as the mean ± SD. ***p < 0.001, ****p < 0.0001 by one-way ANOVA.


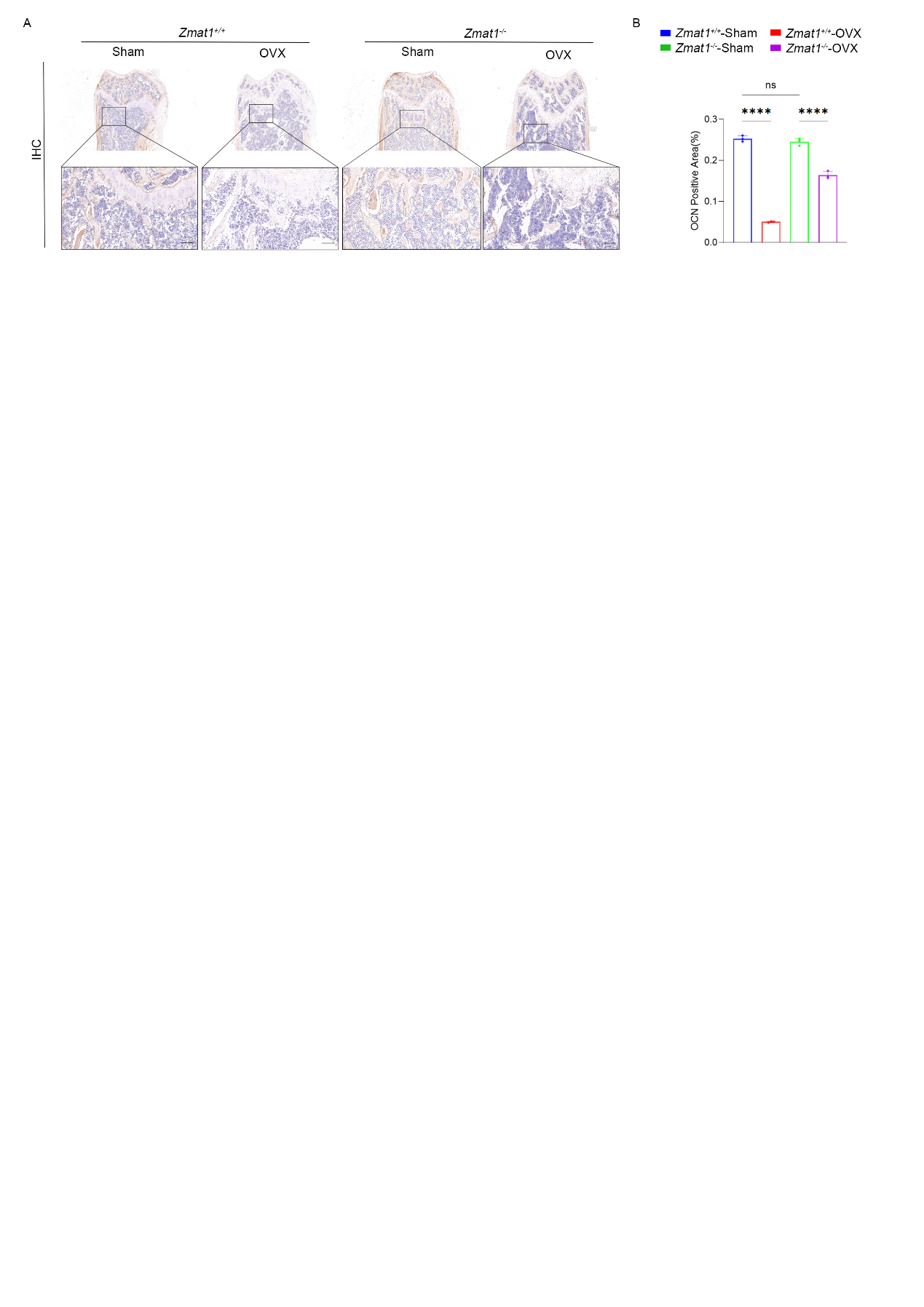


**Supplementary Figure 8. Immunohistochemical analysis of OCN expression in femoral sections from *Zmat1^+/+^* and *Zmat1^−/−^* mice. A** Representative immunohistochemical staining of osteocalcin (OCN) in femoral sections from *Zmat1^+/+^* and *Zmat1^−/−^* mice under Sham or ovariectomy (OVX) conditions. Insets show higher-magnification views of the trabecular bone region. Scale bar = 100 μm. **B** Quantification of OCN-positive area (%). Data are presented as mean ± SD (n = 3). **p < 0.01, ****p < 0.0001 by one-way ANOVA.


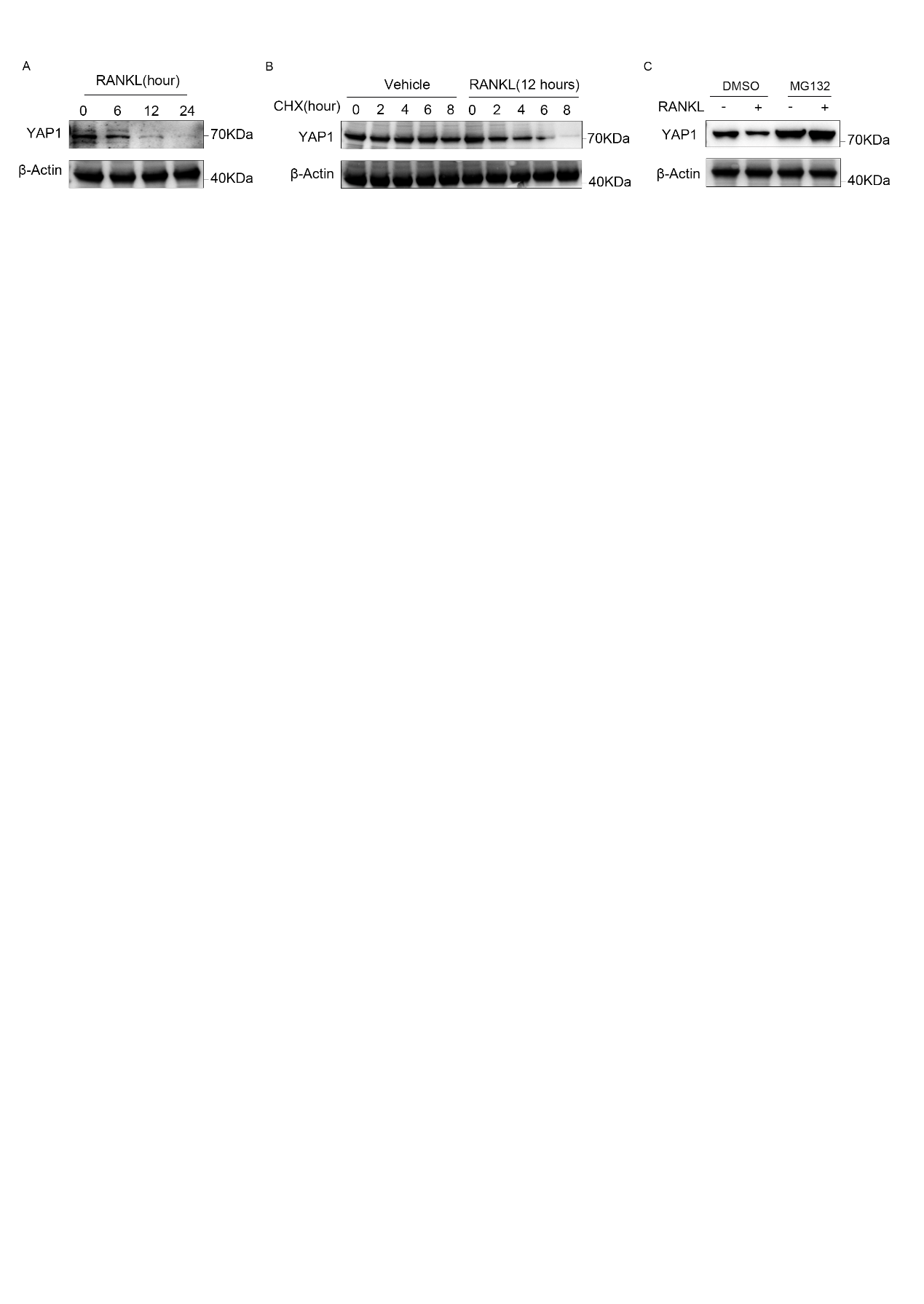


**Supplementary Figure 9. The rapid loss of YAP1 during the early stage of osteoclast differentiation is primarily attributed to proteasome-mediated degradation.** **A** YAP1 protein expression gradually decreased following RANKL stimulation at 0 h, 6 h,12 h and 24 h. **B** CHX chase assays revealed that YAP1 underwent accelerated degradation in the presence of RANKL compared with the vehicle group. **C** YAP1 protein levels under RANKL stimulation with or without the proteasome inhibitor MG132.


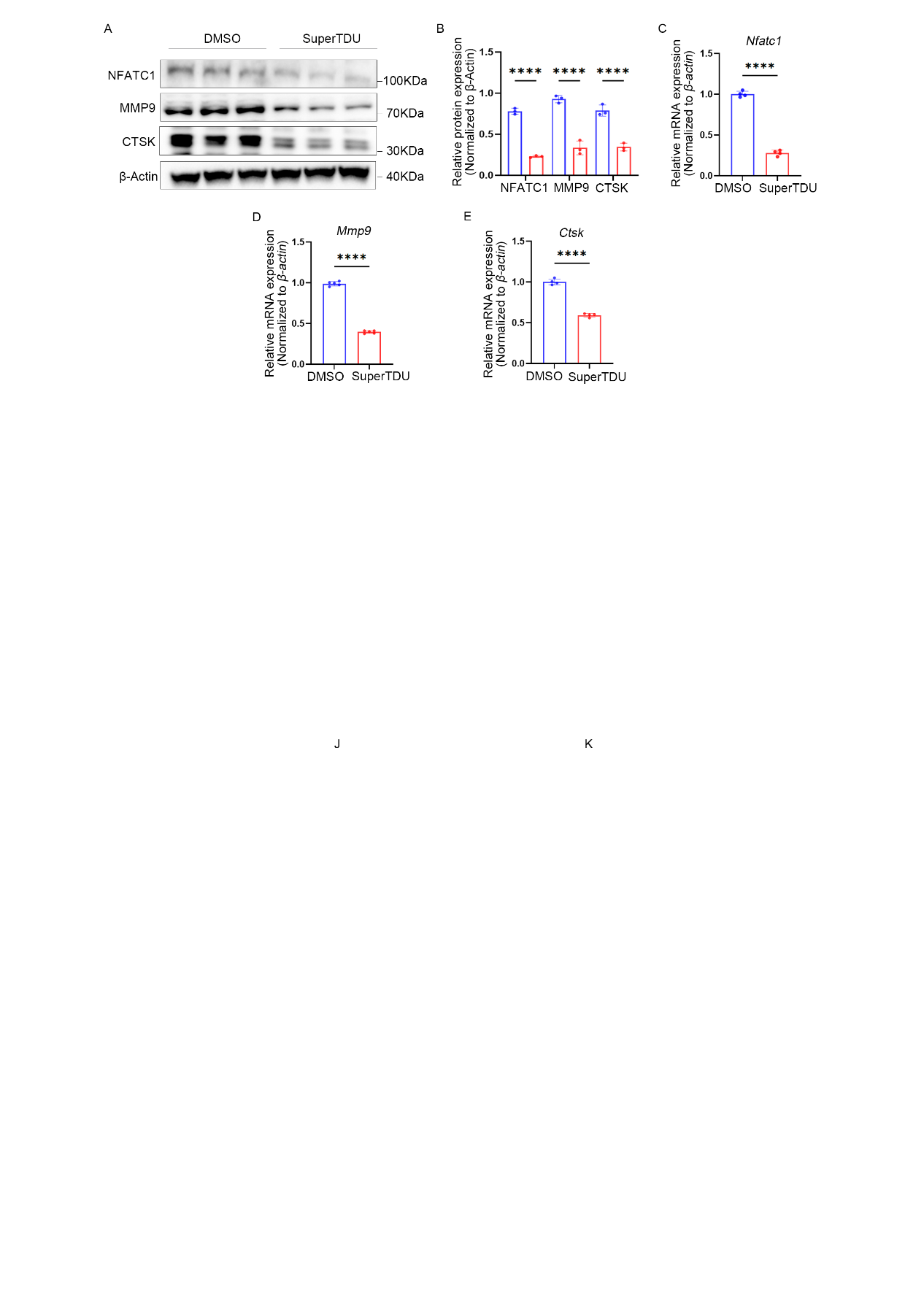


**Supplementary Figure 10. YAP1 directly regulates the formation of osteoclasts**. **A** Representative immunoblotting analysis of NFATC1, MMP9, and CTSK protein expression in BMDMs treated in medium added to 10μM Super TDU with 100 ng/ml RANKL and 50 ng/ml M-CSF followed by the indicated time. **B** Densitometric quantification of NFATC1, MMP9, and CTSK band intensities from three independent experiments. β-Actin was used as a loading control. **C-E** qPCR analysis of *Nfatc1*, *Mmp9*, and *Ctsk* in osteoclastogenesis. Cells were cultured in medium containing 10μM Super TDU with 100 ng/ml RANKL and 50 ng/ml M-CSF. All bar graphs are presented as the mean ± SD. ****p < 0.0001 by Student’s *t* test and two-way ANOVA.


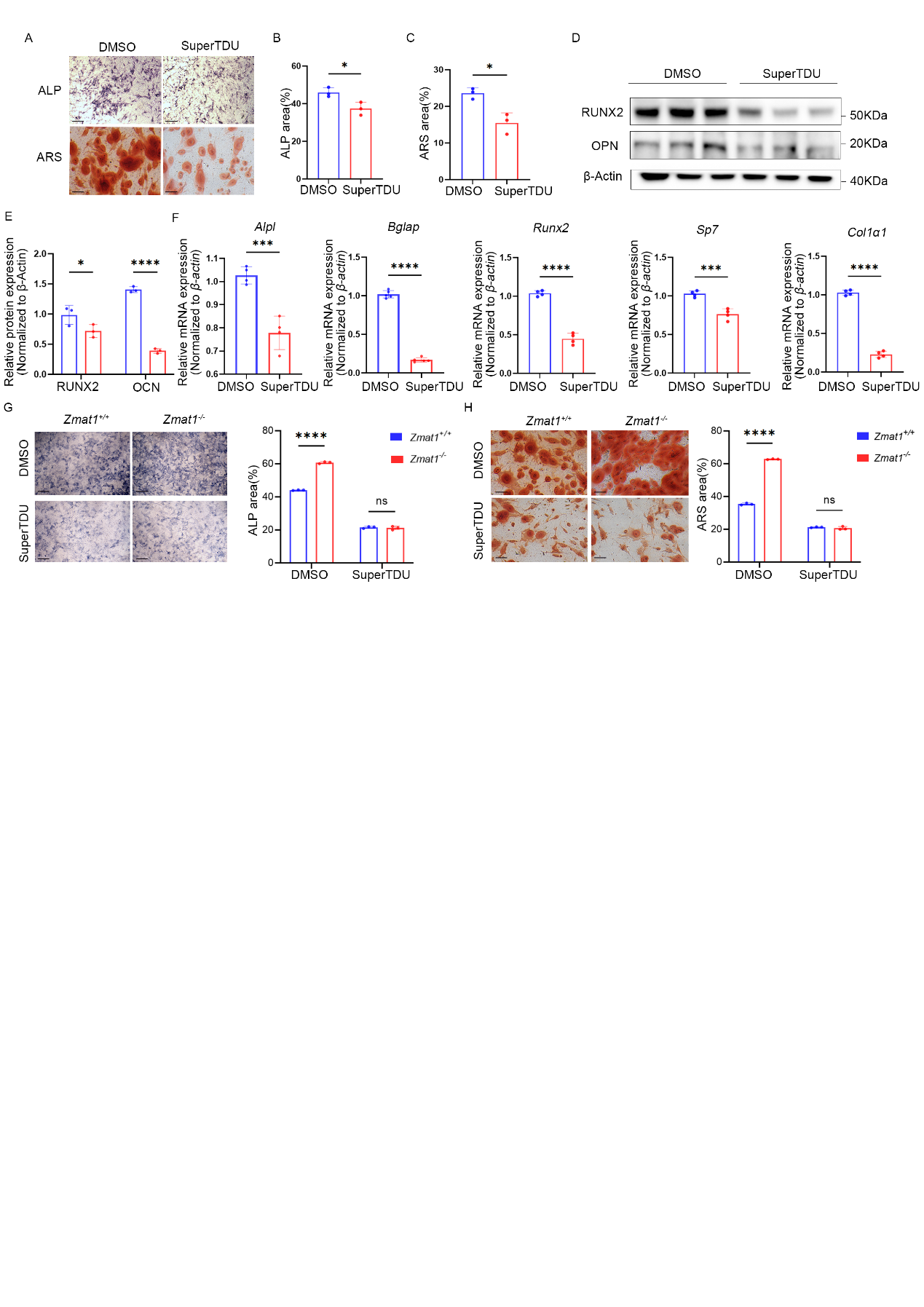


**Supplementary Figure 11. YAP1 regulates the formation of osteoblasts. A** Representative image of ALP and ARS staining of osteoblasts cultured for 21 days in medium containing 10μM Super TDU. Scale bars, 50μm. **B-C** ALP and ARS staining surface percent was calculated for **A**. **D** Representative immunoblotting analysis of RUNX2 and OPN protein expression in osteoblasts cultured in medium containing 10μM SuperTDU. **E** Densitometric quantification of RUNX2 and OPN band intensities from three independent experiments. β-Actin was used as a loading control. **F** qPCR analysis of osteogenic biomarker genes *Alpl*, *Bglap*, *Runx2*, *Sp7*, and *Col1α1* in osteoblasts with osteogenic induction. **G** Representative images of ALP staining and surface percent were calculated for osteoblasts from *Zmat1^+/+^* and *Zmat1^-/-^* mice cultured in medium containing SuperTDU. Scale bars, 50μm. **H** Representative images of ARS staining and quantification of ARS-positive surface area in osteoblasts derived from *Zmat1^+/+^* and *Zmat1^-/-^* mice cultured in medium containing SuperTDU. Scale bars, 50μm. All bar graphs are presented as the mean ± SD. *p < 0.05; ***p < 0.001; ****p < 0.0001; n.s. not significant by Student’s *t* test and two-way ANOVA.


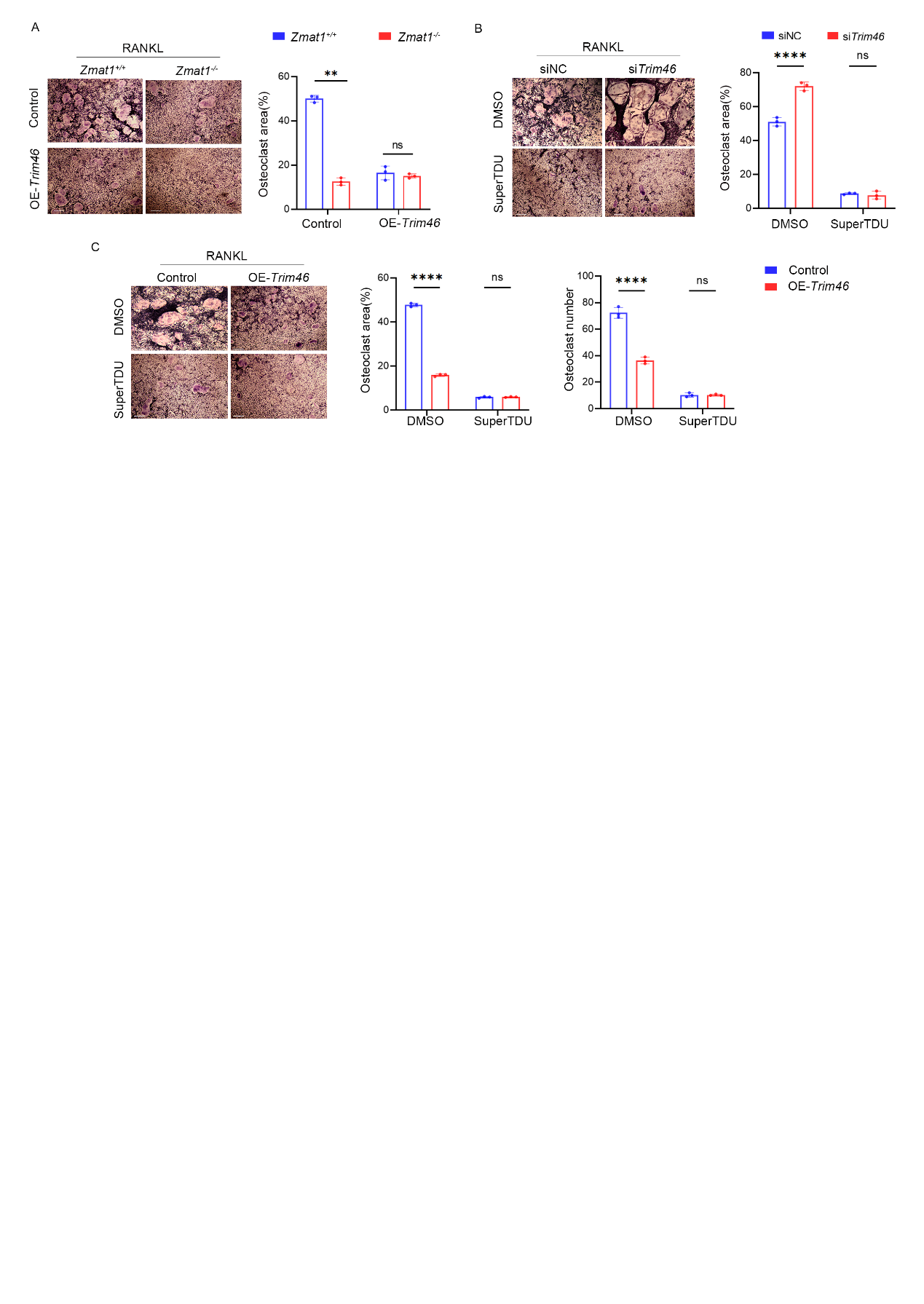


**Supplementary Figure 12. *Trim46* promotes osteoclastogenesis in a YAP1-dependent manner. A** Representative TRAP staining images (left) and quantification of osteoclast area (right). *Zmat1^⁺/⁺^* and *Zmat1^⁻/⁻^* BMDMs were transfected with a control vector or a *Trim46* overexpression plasmid (OE-Trim46), followed by RANKL stimulation. **B** Representative TRAP staining images and quantification showing the effect of YAP1 inhibition on *Trim46*-depleted cells. BMDMs were transfected with si*Trim46* or siNC and treated with DMSO or the YAP1 inhibitor SuperTDU (10 μM).

**C** Representative TRAP staining images and quantification of BMDMs transfected with OE-Trim46 or control vector and treated with DMSO or the YAP1 inhibitor SuperTDU (10 μM). Scale bars, 100μm. All bar graphs are presented as the mean ± SD. ****p < 0.0001, **p < 0.01, *p < 0.1; n.s. not significant by two-way ANOVA.

Supplemental Table 1. The sequence of gene-specific primers used for qPCR

| Specifies | Gene name | Sequence of forward and reverse Primers (5’ to 3’) |
| --- | --- | --- |
| Mus musculus | *Zmat1*  *Nfatc1*  *Ctsk*  *Calcr*  *Itgb3*  *Dcstamp*  *β-actin*  *Yap1*  *Cyr61*  *Mst1*  *Lats1*  *Trim46*  *ChIP-1*  *ChIP-2*  *ChIP-3*  *ChIP-4*  *ChIP-5*  *ChIP-Ctrl*  *Runx2*  *Bglap*  *Alpl* | ATGCTCACCAAAGATGGATGAACCC  ATGCTGGAGGAGGCTTCACAATAAC  GACCCGGAGTTCGACTTCG  TGACACTAGGGGACACATAACTG  GAAGAAGACTCACCAGAAGCAG  TCCAGGTTATGGGCAGAGATT  GCAACGCTTTCACTTCTGAGA  GTTCCCACTGCATTGTCCACA  CCACACGAGGCGTGAACTC  CTTCAGGTTACATCGGGGTGA  GGGGACTTATGTGTTTCCACG  ACAAAGCAACAGACTCCCAAAT  GGCTGTATTCCCCTCCATCG  CCAGTTGGTAACAATGCCATGT  ACCCTCGTTTTGCCATGAAC  TGTGCTGGGATTGATATTCCGTA  GCAGATCCCTTTCAGAGCGG  CTCACCACTGAATGACTTCCAG  AAGGCCCGACAGTCCAGAA  AAAGCCAGAAGGGTACAGACA  CCTCAGGGATTCTCGGATCTC  CCTCAGGGATTCTCGGATCTC  GGTGAGGATATGCAGACCTTCA  TTGTGGGTACAAGGCAGCAC  CTTTCTTCTGTGTATGTTCGGGG  ACGTTTCATTTAACTTCCTGGTCC  CTTCTGTGTATGTTCGGGGGTTG  CACGTTTCATTTAACTTCCTGGTC  TTCTTCTGTGTATGTTCGGGGGT  CCACGTTTCATTTAACTTCCTGGTC  TTCTGTGTATGTTCGGGGGTTGG  TCCACGTTTCATTTAACTTCCTGGT  TGCGTTTTCGCTTTCTTCTGTGTAT  CATTTAACTTCCTGGTCCCCCAAC  TGTACTGGACCCAAGCGACA  ATGGTTGGAAAAGGGGTTGAGTAT  ATGCTTCATTCGCCTCACAAA  GCACTCACTGACTCGGTTGG  CTTGGTGCACACCTAGCAGA  CTCCCTCATGTGTTGTCCCT  CCAACTCTTTTGTGCCAGAGA  GGCTACATTGGTGTTGAGCTTTT |

| Homo sapiens | *Sp7*  *Col1α1*  *Zmat1*  *Ctsk*  *Runx2* | ATGGCGTCCTCTCTGCTTG  TGAAAGGTCAGCGTATGGCTT  GCTCCTCTTAGGGGCCACT  CCACGTCTCACCATTGGGG  CTGCTGACCATCAAGCAGGT  TGGACTGCTCTTTCTCTGGC  AGGCAGCTAAATGCAGAGGG  CAATGGCCACAGAGACAGGT  GCGGTGCAAACTTTCTCCAG  GACTCTGTTGGTCTCGGTGG |
| --- | --- | --- |

Supplemental Table 2. The sequence of siRNA

| Specifies | Gene name | Sequence of forward and reverse siRNA (5’ to 3’) |
| --- | --- | --- |
| Mus musculus  Homo sapiens | *Zmat1*  *Trim46* | CTGAACATCAAATTAAAGAATCT  GACTTGTAGTTTAATTTCTTAGA  TTCATGGTTTATAAACAATAAAC  AAGTACCAAATATTTGTTATTTG |
|  | *Crip1* | TGAGAAATGTGGAAAGACA |
|  |  | ACAGAAAGGTGTAAAGAGT |
|  | *Nr2f2* | AUAUAUGGCCAGUUAAAACUG |
|  |  | GUCAAAUUGACCUGUAUAUA |
|  | *Fhl1* | AAGGAGGTGCACTATAAGAAC |
|  |  | CAAGAATATCACTGGAGGAA |
|  | *Nfib* | AAGCCACAAUGAUCCUGCCAAGAAU |
|  |  | AUUCUUGGCAGGAUCAUUGUGGCUU |
|  | *Nfia*  *Ebf1*  *Zmat1* | GAAAGUUCUUCAUACUACAGCAUGA |
|  |  | AGUACGACAUCAUUCUUUGAAAG  UACACUAUGGCUUGUUUUGUC  AUGUGAUACCGAACAAAAACAG  UUUGCAUUUGAUUUUGCUGGA  CAGCAAAAUCAAAUGCAAAUG |

Supplemental Table 3. The category of antibodies

| Specifies |  | Product name |
| --- | --- | --- |
| Mus musculus | CathepsinK  NFATc1  OCN  β-Actin  YAP  Flag  V5  Myc  HA | CST, #57056  CST, #5861  CST, #59757  CST, #8457  CST, #14074  CST, #14793  CST, #13202  CST, #18583  CST, #3724 |

Supplemental Table 4. The category of inhibitors

| Specifies |  | Product name |
| --- | --- | --- |
|  | Super TDU  CQ  Baf A1  MG132  DMSO | MCE, HY-P1727  MCE, HY-17589A  MCE, HY-100558  Merck, M7449  MCE, HY-Y0320 |
