# Supplementary material for: ZMAT1 Promotes Osteoclastogenesis Through TRIM46 Mediated YAP1 Degradation and Inhibits Osteoblastogenesis
Source: Adv Sci (Weinh). 2026 Mar 2;13(27):e21783. doi: 10.1002/advs.202521783 (PMC13170258; doi:10.1002/advs.202521783)
Supplement: Supplementary file 2 — Supporting File 2: advs74602‐sup‐0002‐SourceData.docx. [file ADVS-13-e21783-s001.docx]

Fig2A-Zmat1^+/+^


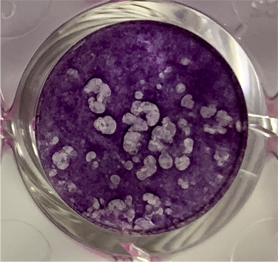

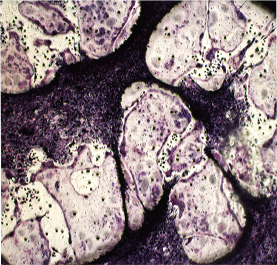


Fig2A-Zmat1^-/-^


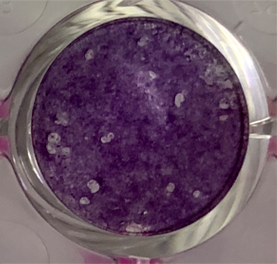

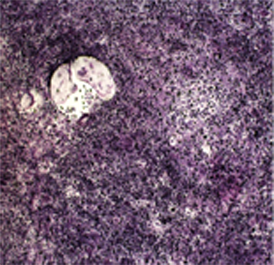


Fig2B- Zmat1^+/+^-F-actin


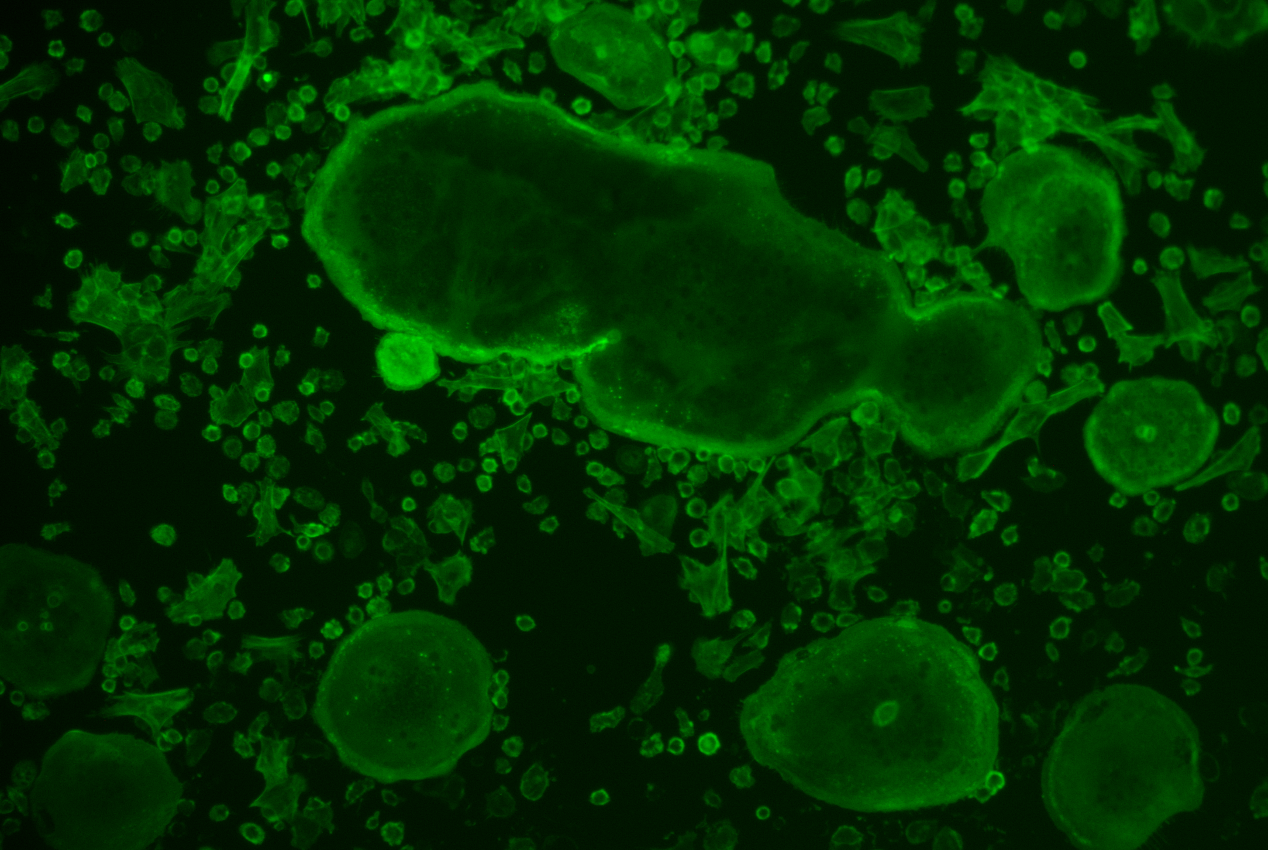


Fig2B- Zmat1^+/+^-DAPI


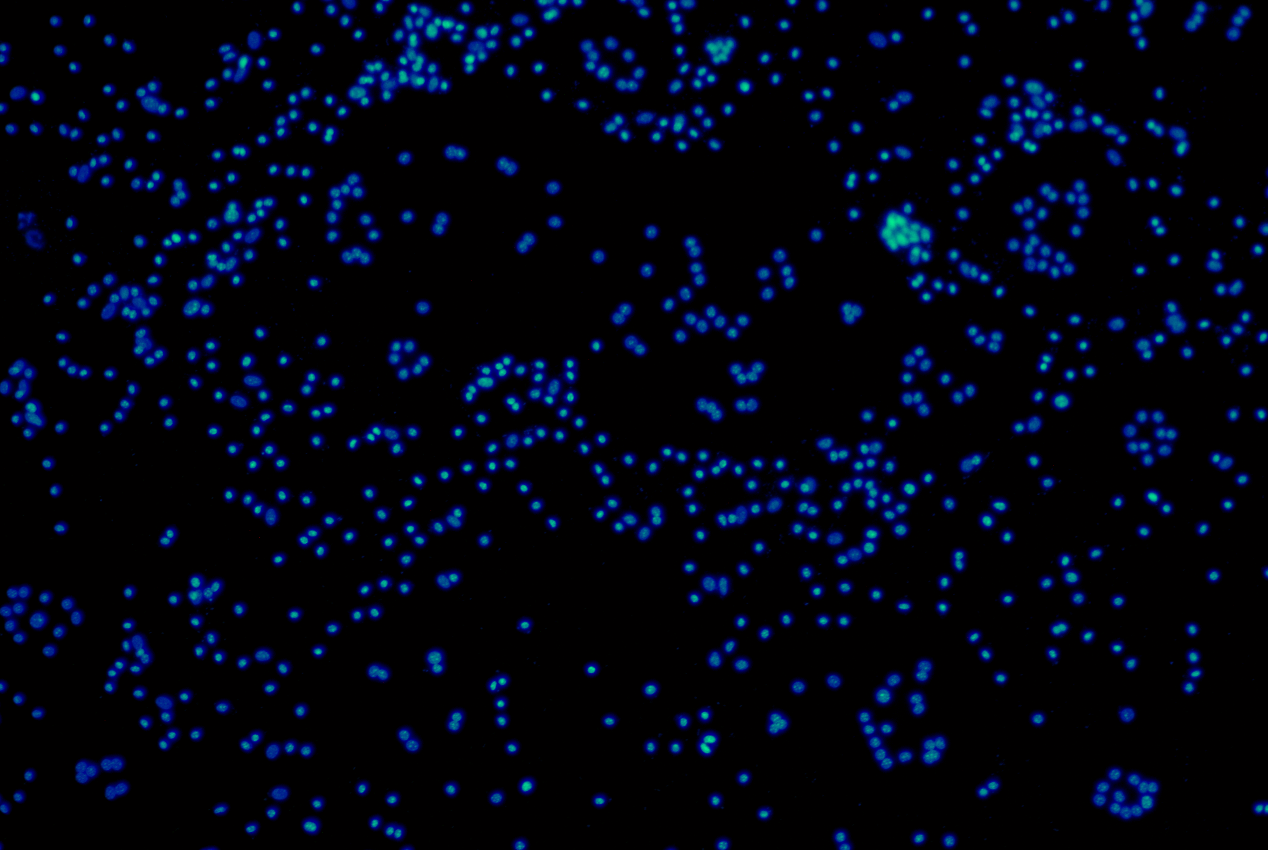


Fig2B- Zmat1^+/+^-Merge


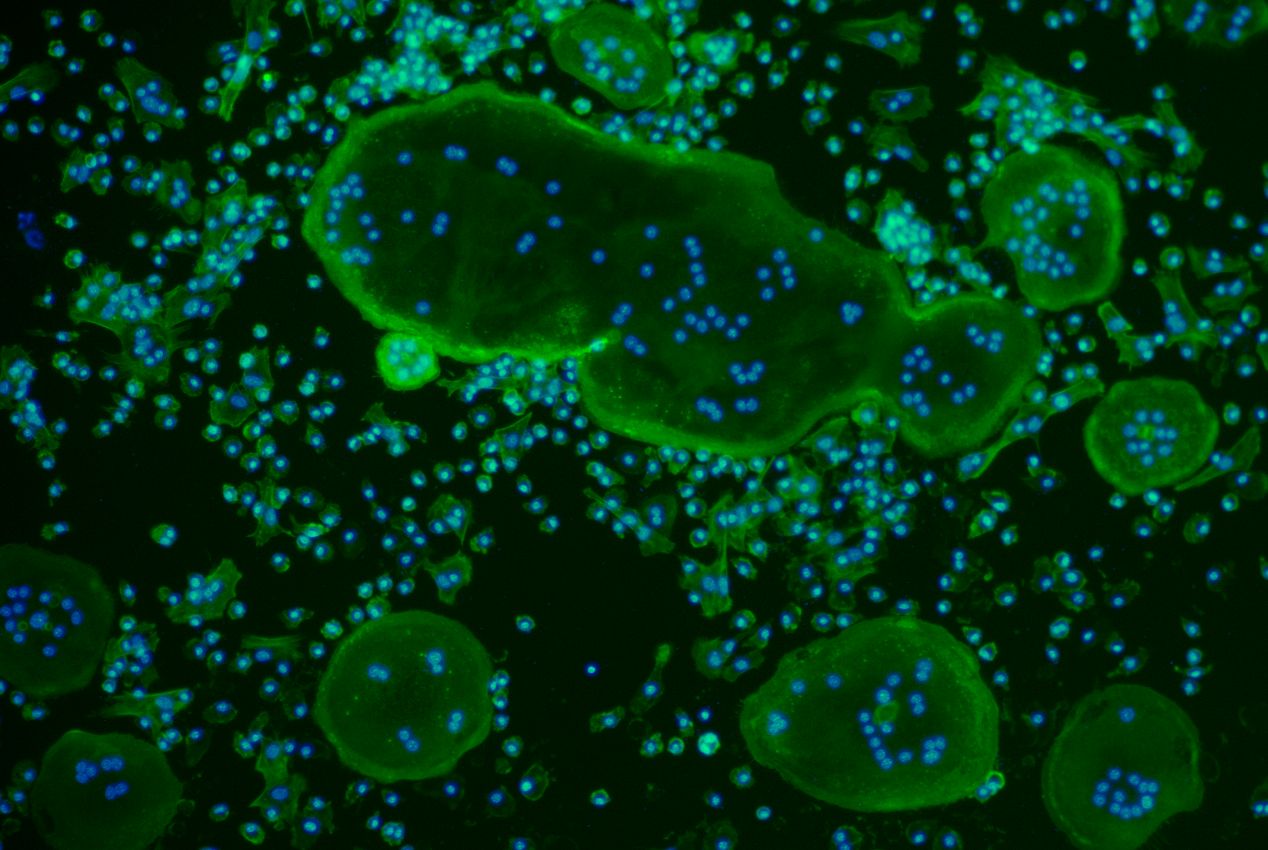


Fig2B- Zmat1^-/-^-Factin


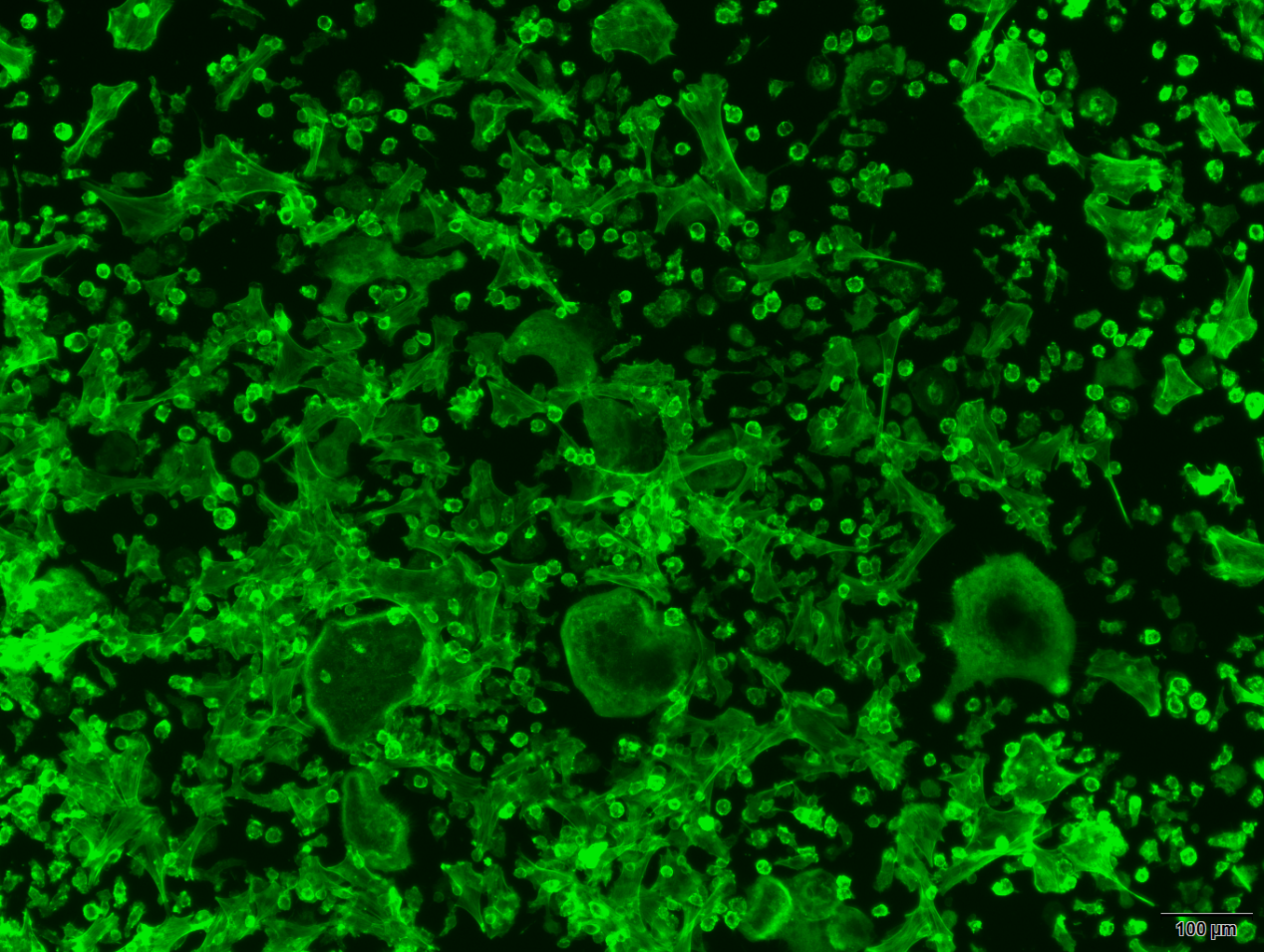


Fig2B- Zmat1^-/-^-DAPI


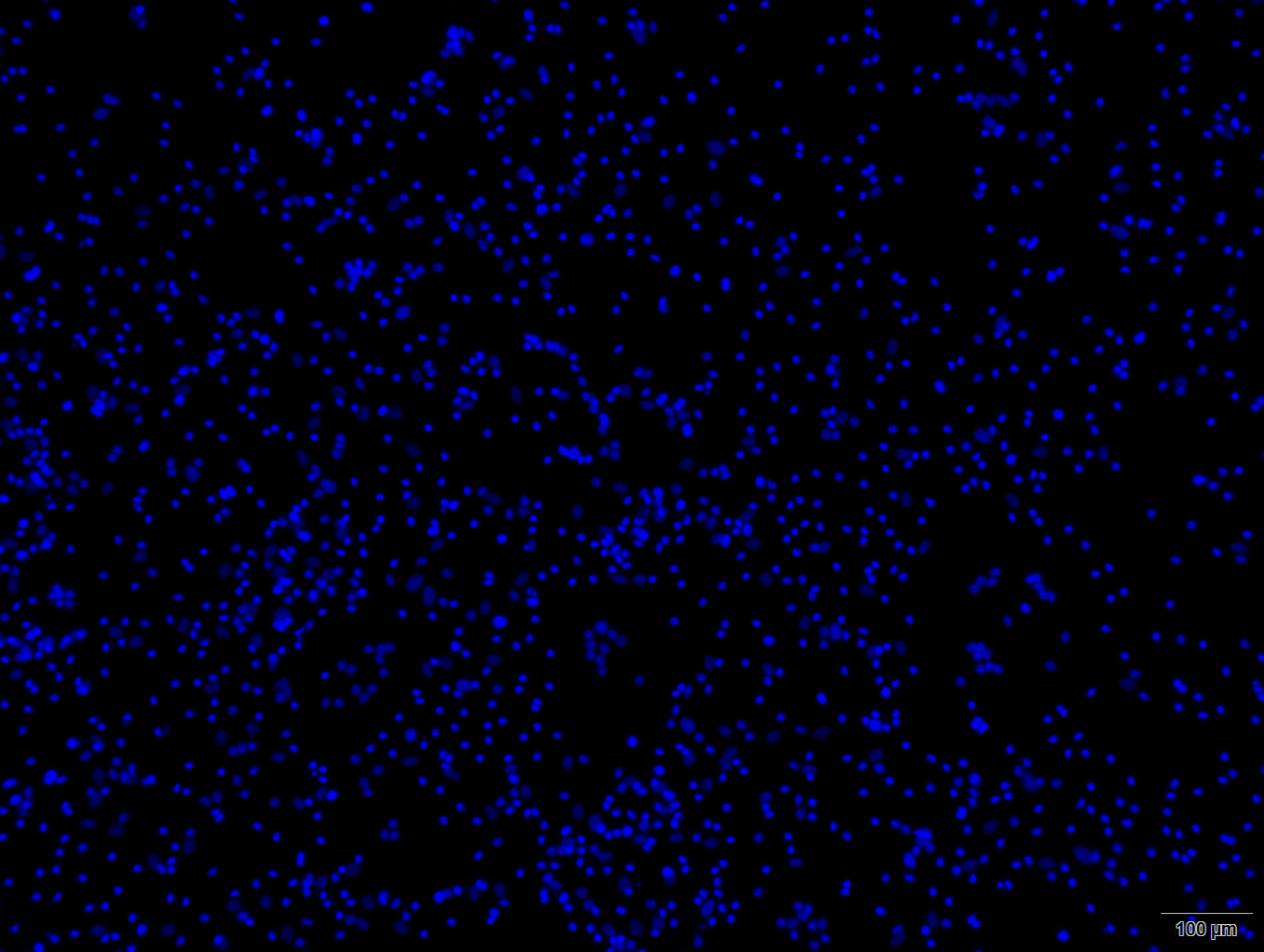


Fig2B- Zmat1^-/-^-Merge


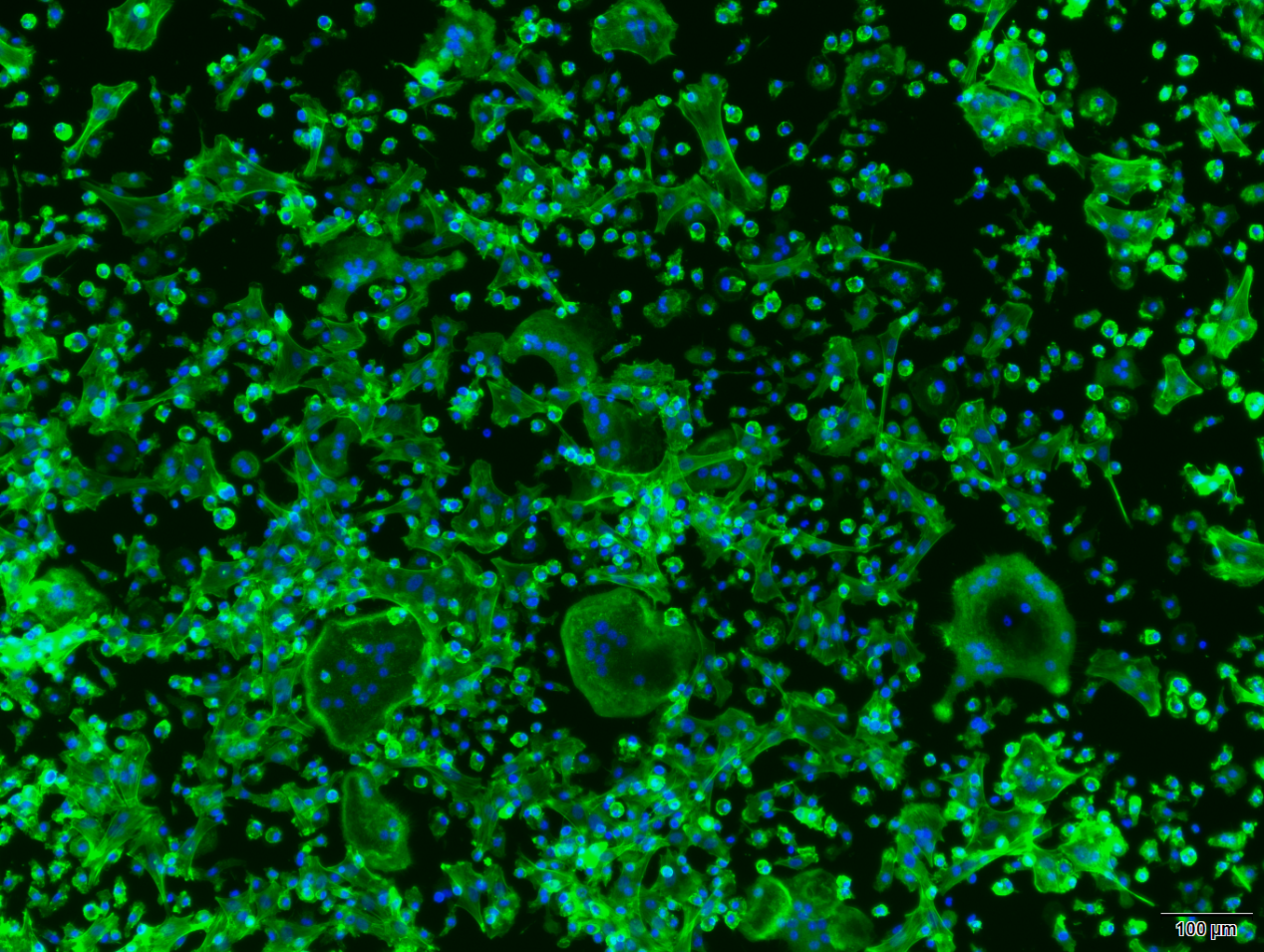


Fig2C- Zmat1^+/+^


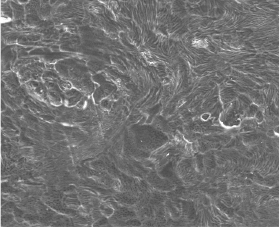


Fig2C- Zmat1^-/-^


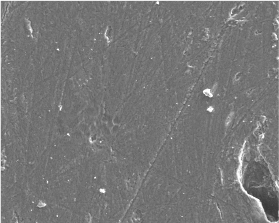


Fig2D


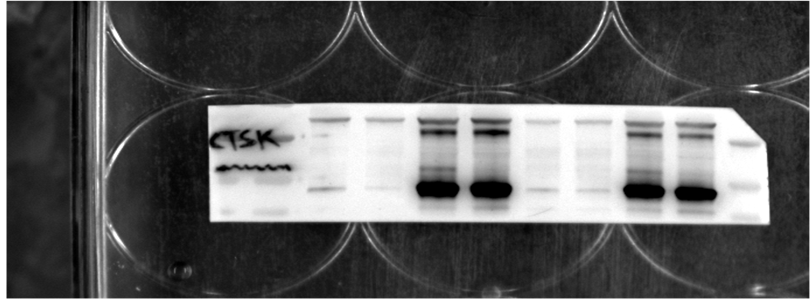

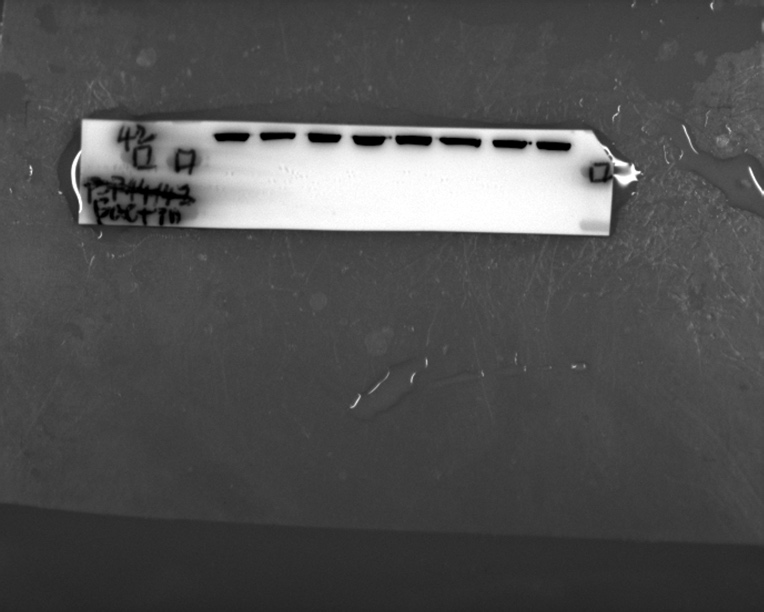


marker(40kDa)

β-Acitn(42kDa)

marker(35kDa)

CathepsinK(37kDa)


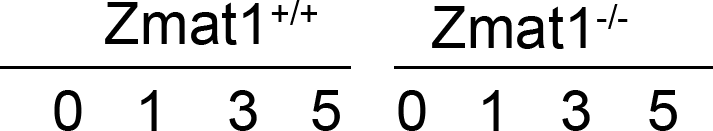


RANKL

Fig3A-Zmat1^+/+^-Sham


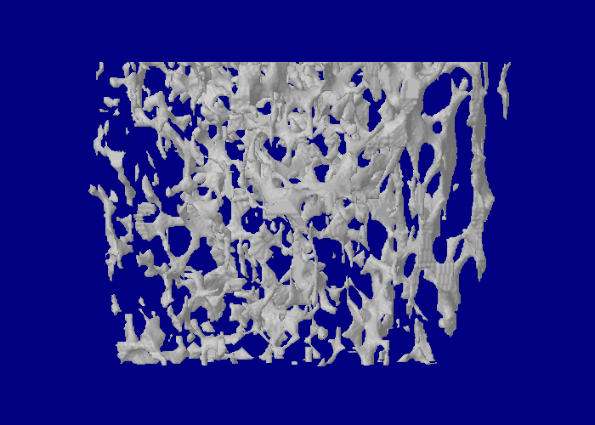

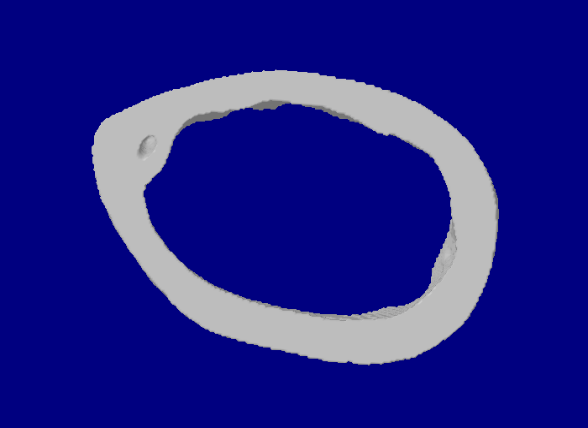


Fig3A-Zmat1^+/+^-OVX


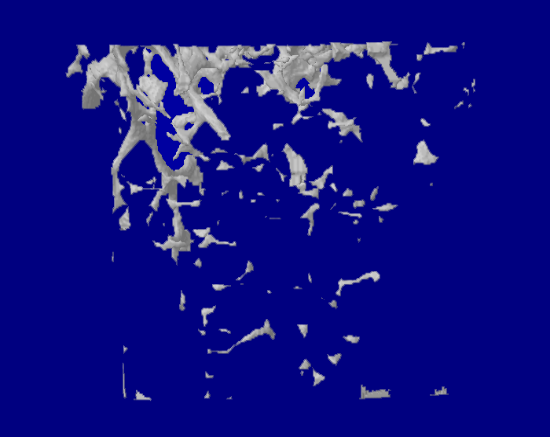

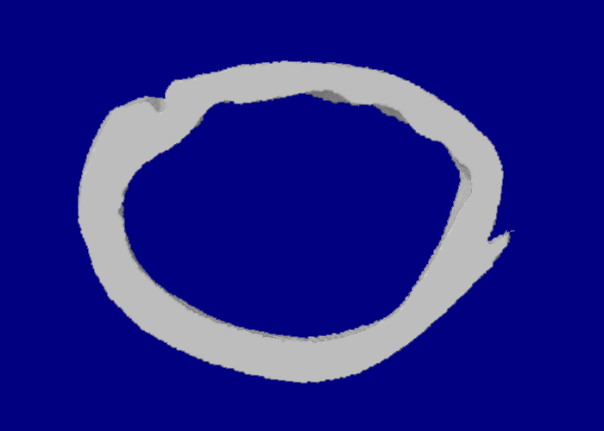


Fig3A-Zmat1^-/-^-Sham


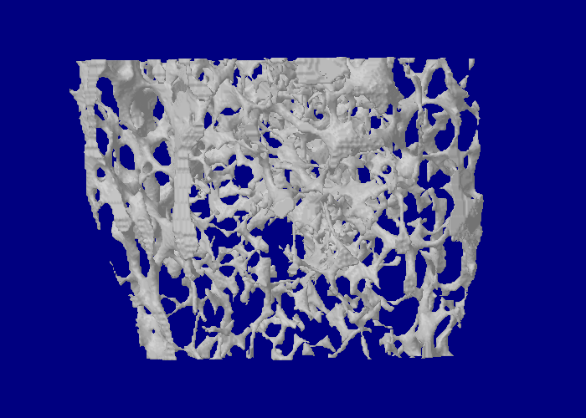

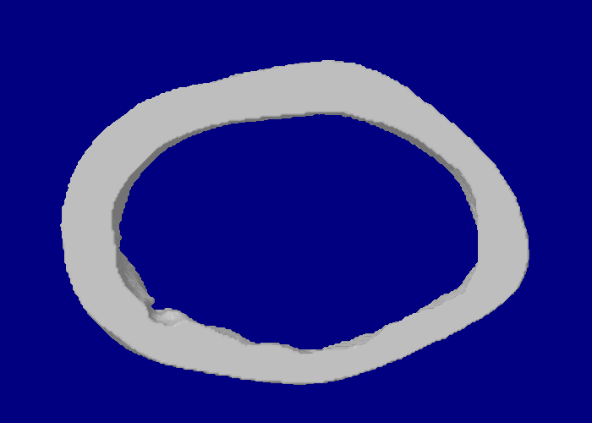


Fig3A-Zmat1^-/-^-OVX


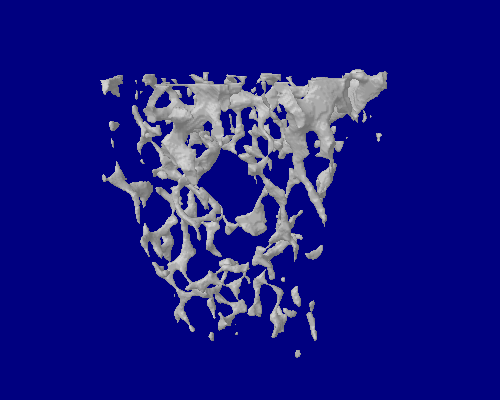

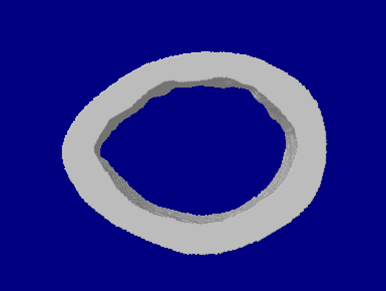


Fig3D-Zmat1^+/+^-Sham


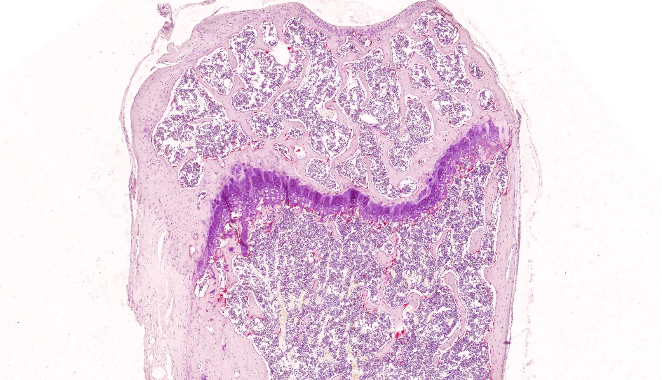


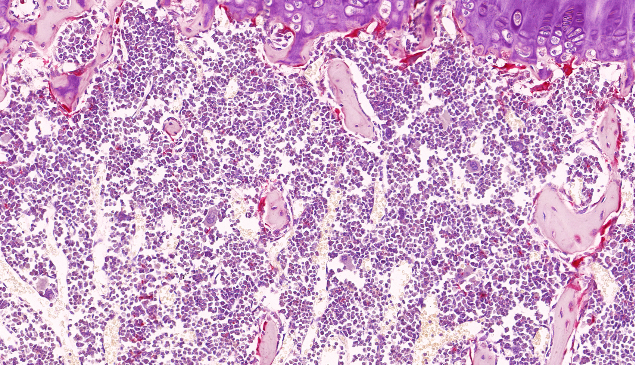


Fig3D-Zmat1^+/+^-OVX


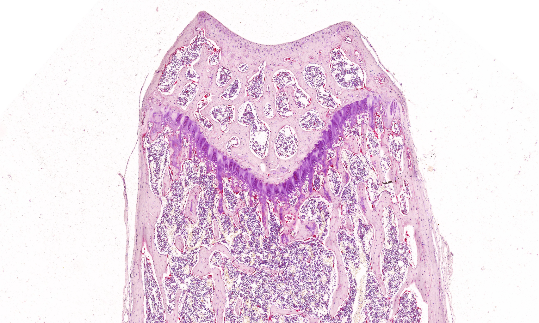


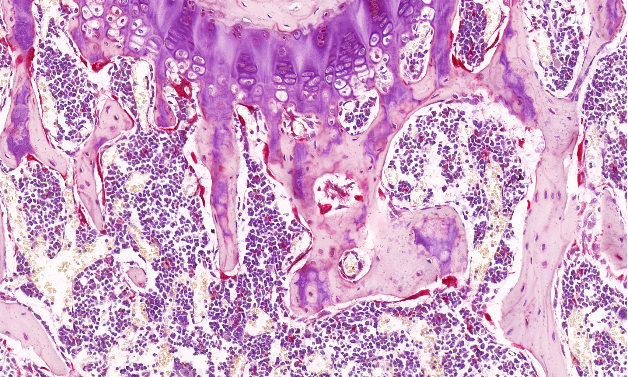


Fig3D-Zmat1^-/-^-Sham


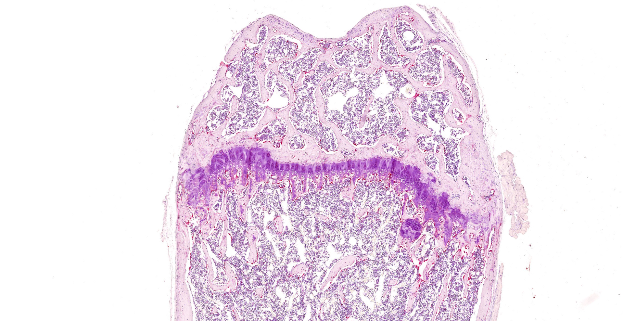


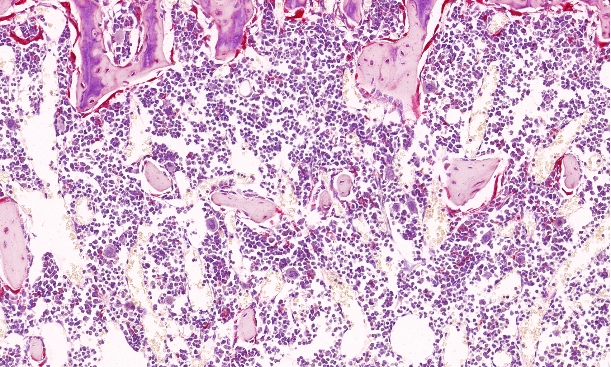


Fig3D-Zmat1^-/-^-OVX


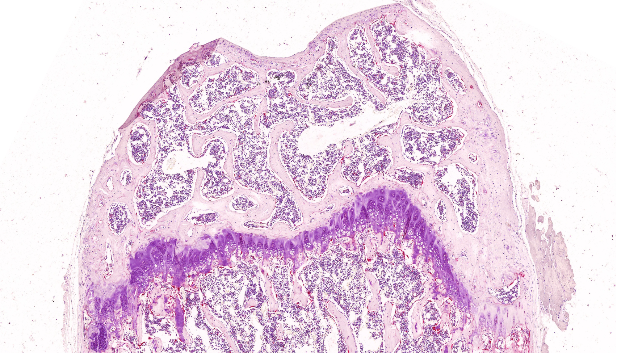


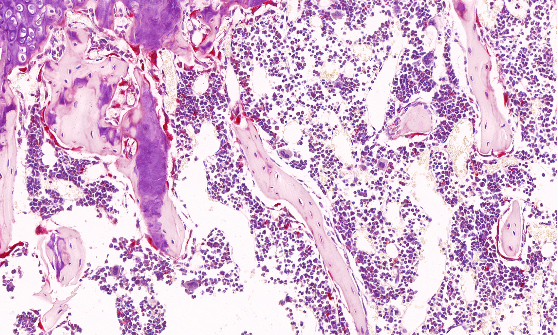


Fig3F-Zmat1^+/+^-Sham


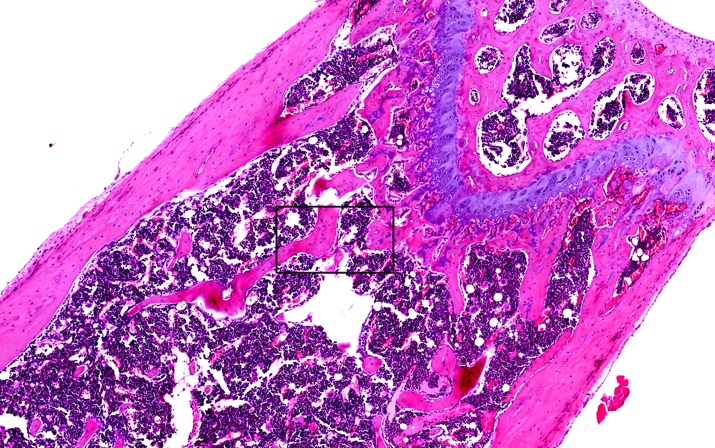


^
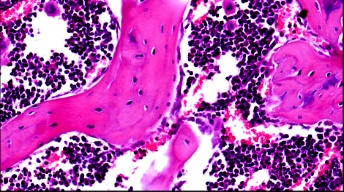
^

Fig3F-Zmat1^+/+^-OVX


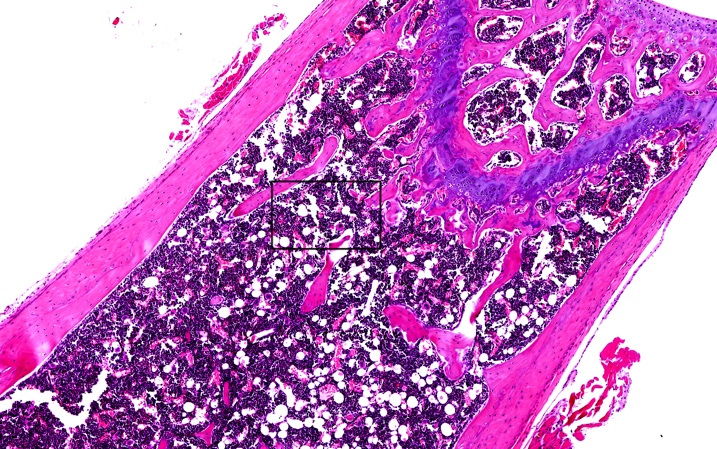


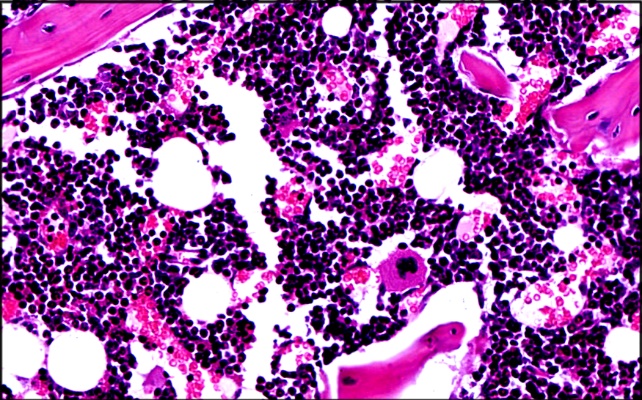


Fig3F-Zmat1^-/-^-Sham


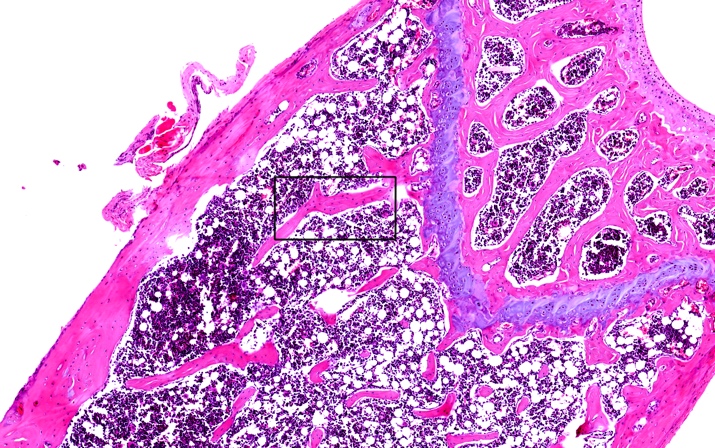


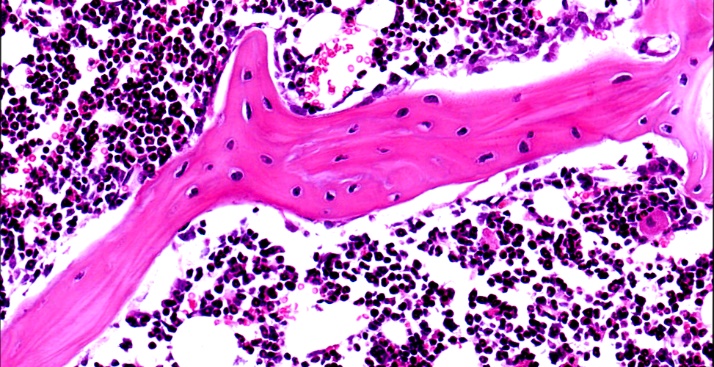


Fig3F-Zmat1^-/-^-OVX


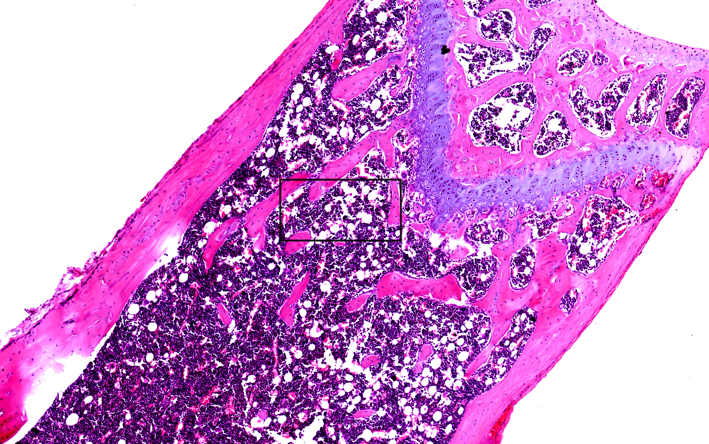


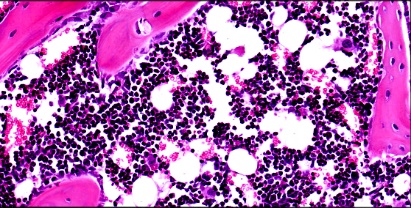


Fig4B-Sham-Zmat1^+/+^-WT


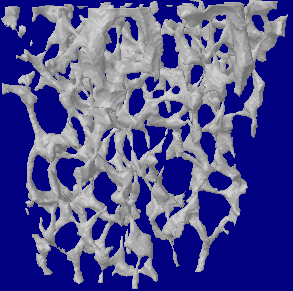

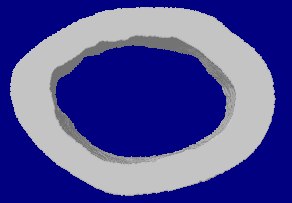


Fig4B-Sham-Zmat1^-/-^-WT


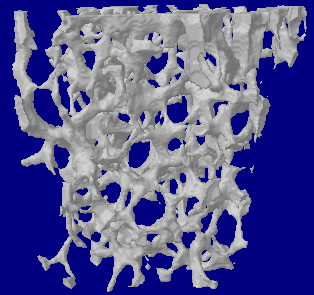

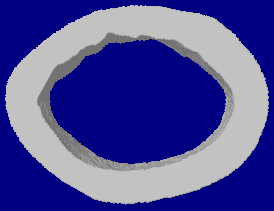


Fig4B-OVX-Zmat1^+/+^-WT


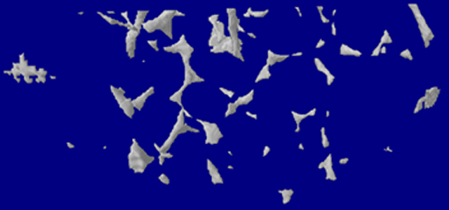

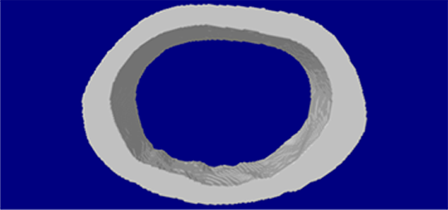


Fig4B-OVX-Zmat1^-/-^-WT


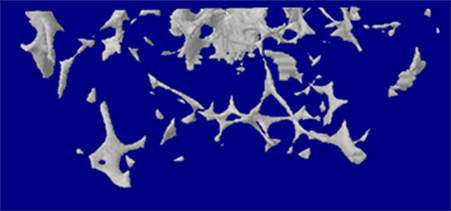

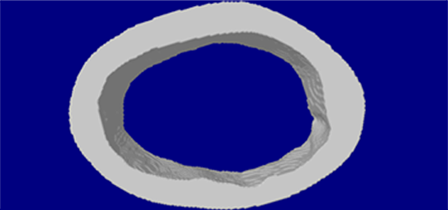


Fig4D-sham-Zmat1^+/+^-WT


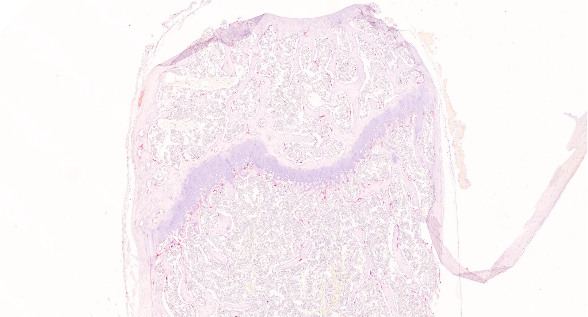

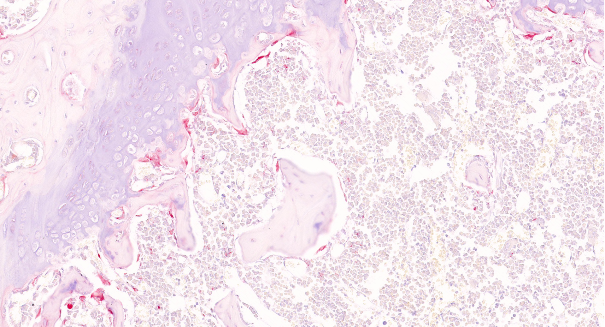


Fig4D- sham-Zmat1^-/-^-WT


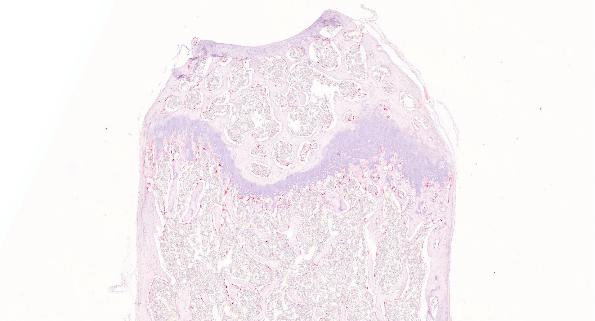

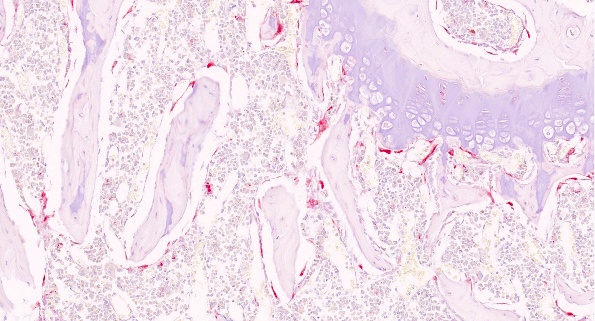


Fig4D-OVX-Zmat1^+/+^-WT


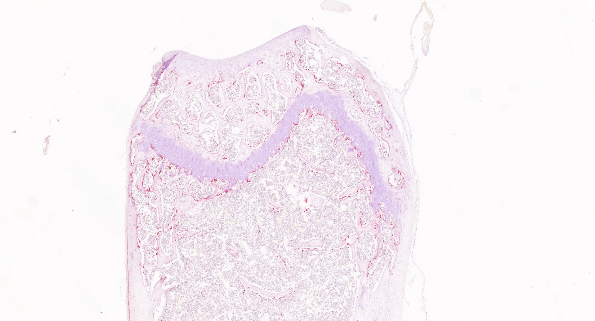

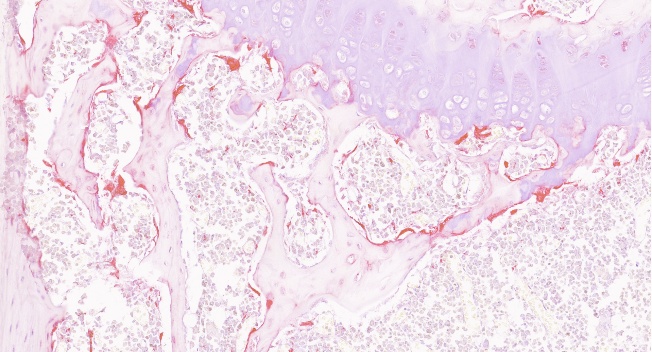


Fig4D- OVX-Zmat1^-/-^-WT


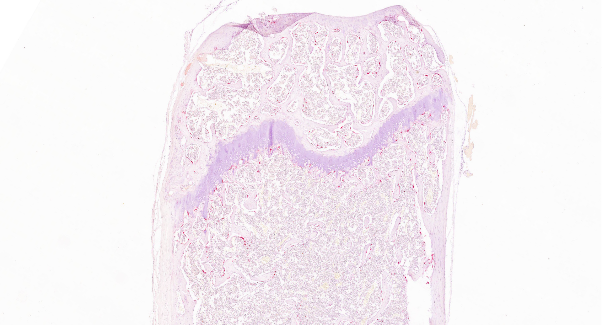

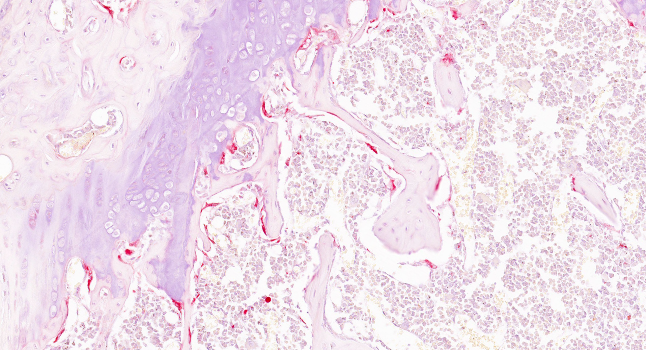


Fig4F-sham-Zmat1^+/+^-WT


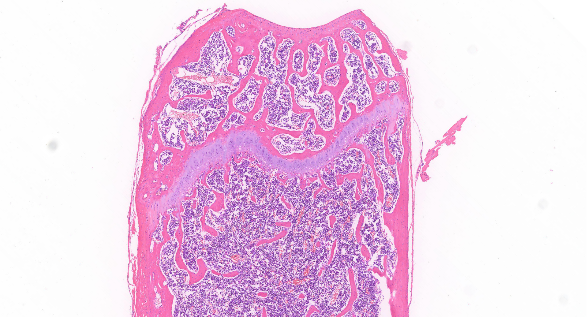

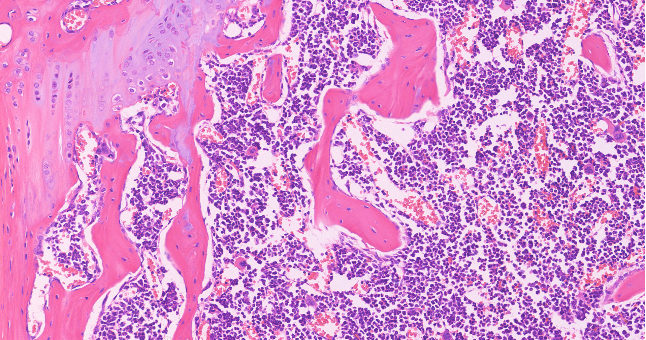


Fig4F- sham-Zmat1^-/-^-WT


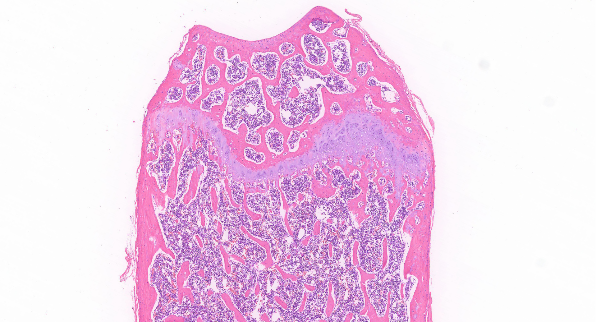

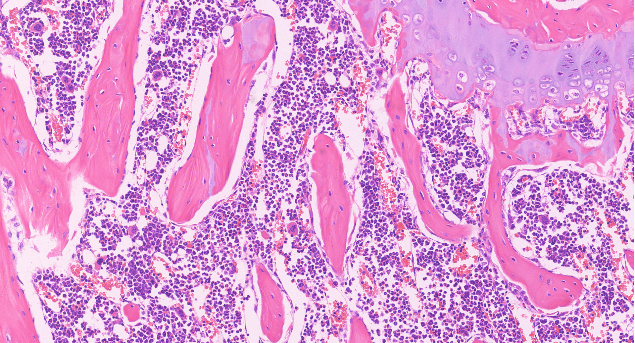


Fig4F-OVX-Zmat1^+/+^-WT


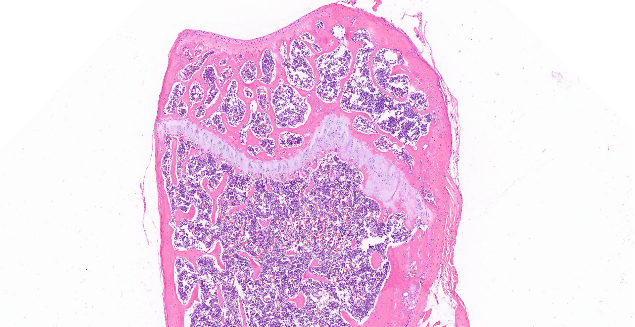

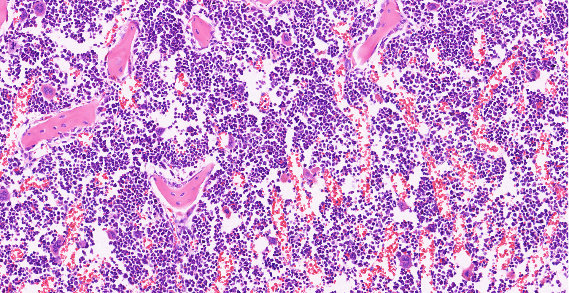


Fig4F- OVX-Zmat1^-/-^-WT


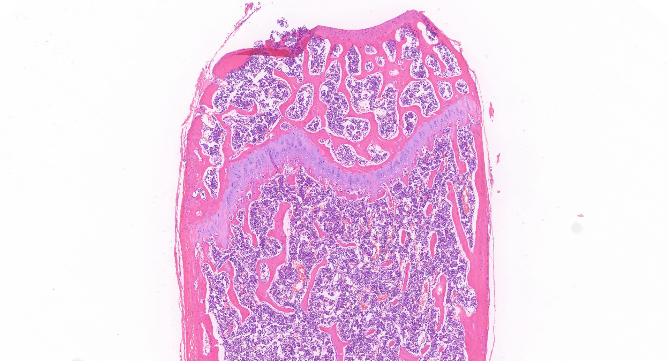

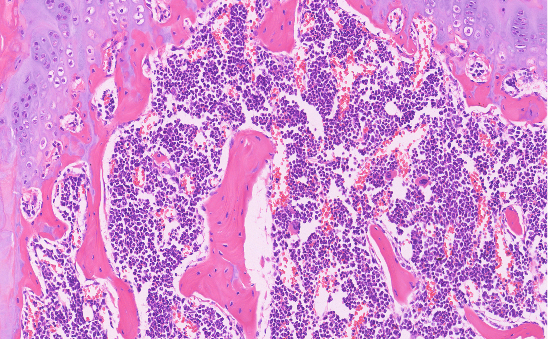


Fig5B-Zmat1^+/+^


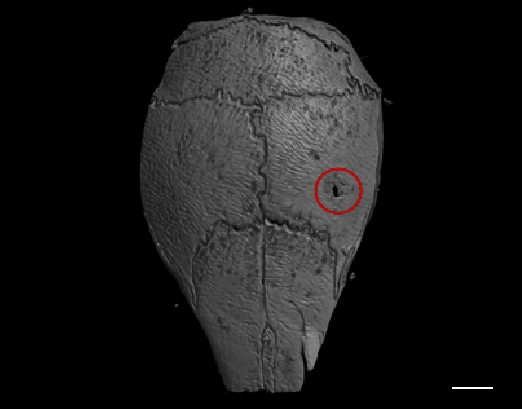


Fig5B-Zmat1^-/-^


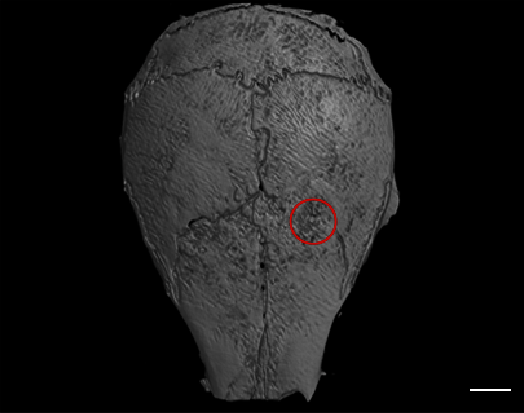


Fig5D-Zmat1^+/+^


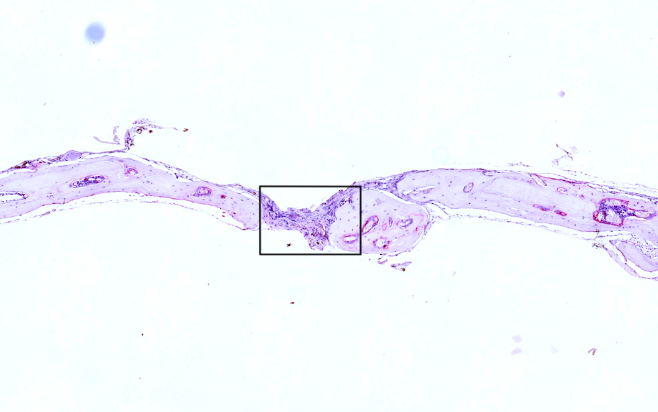


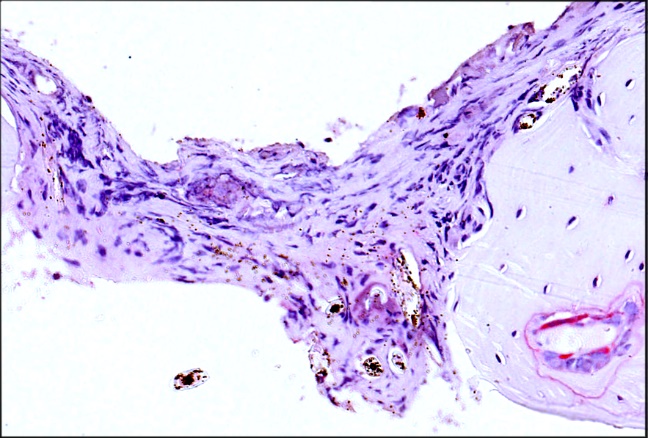


Fig5D-Zmat1^-/-^


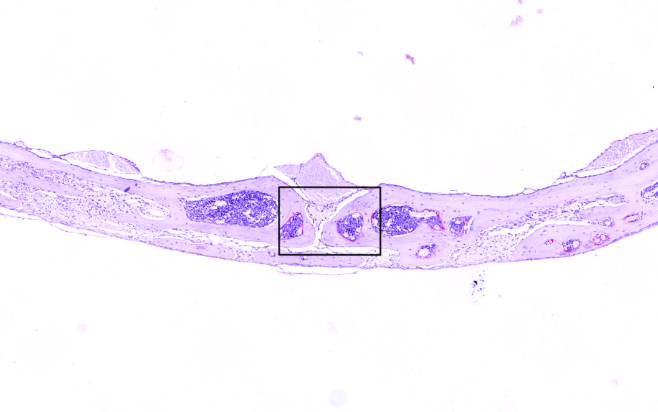


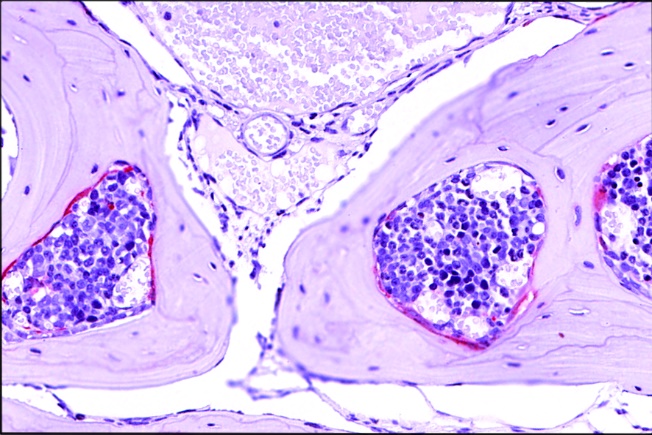


Fig5F-Zmat1^+/+^


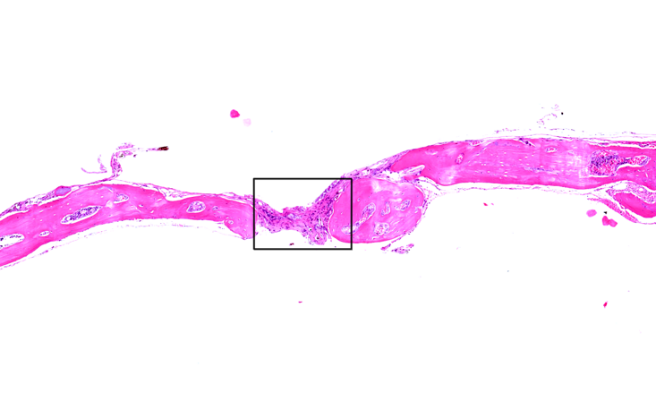


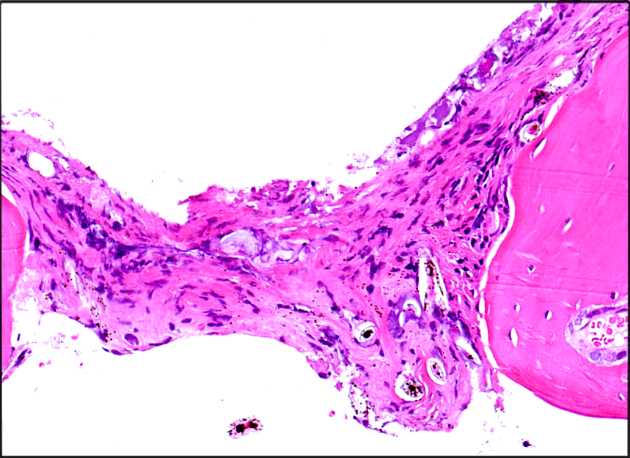


Fig5F-Zmat1^-/-^


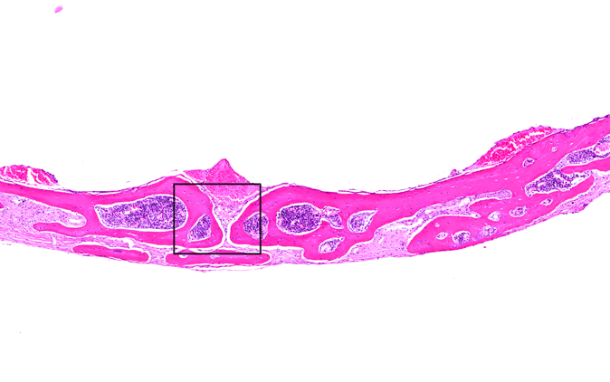


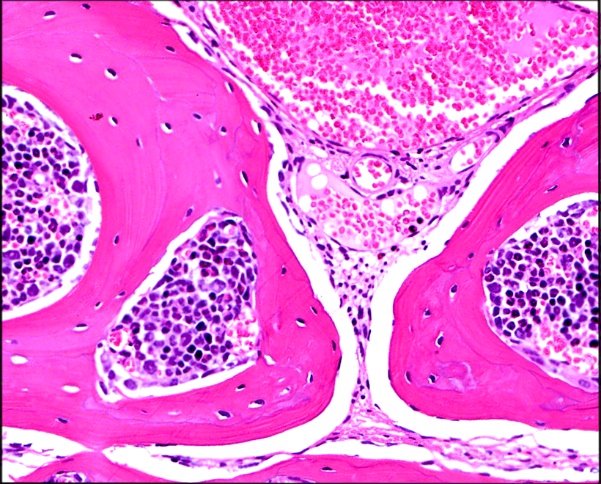


Fig5H-Zmat1^+/+^


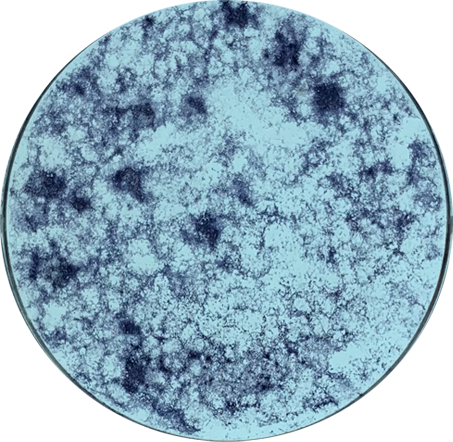

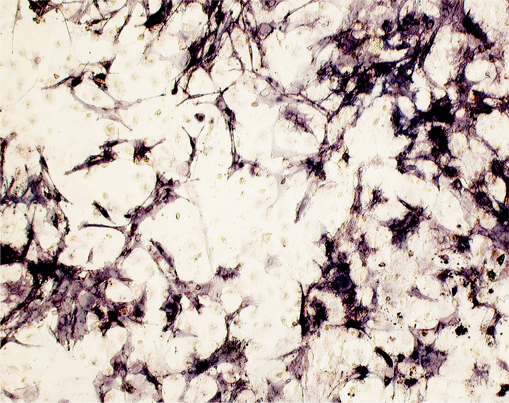


Fig5H-Zmat1^-/-^


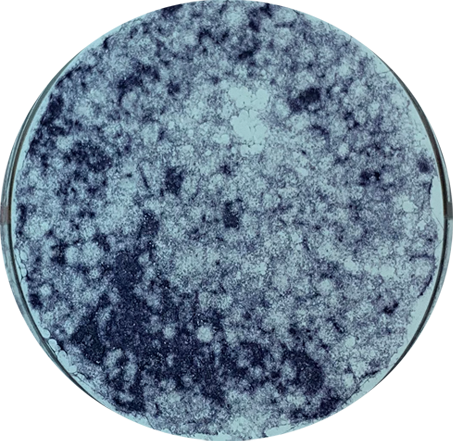

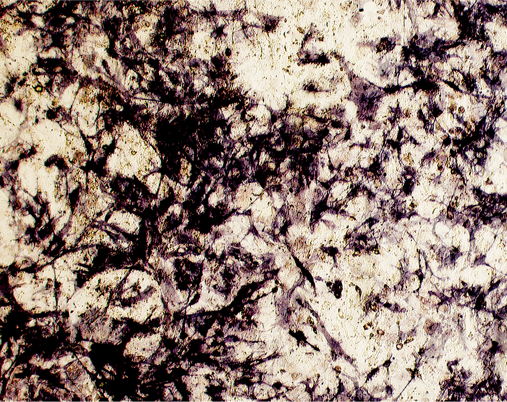


Fig5J-Zmat1^+/+^


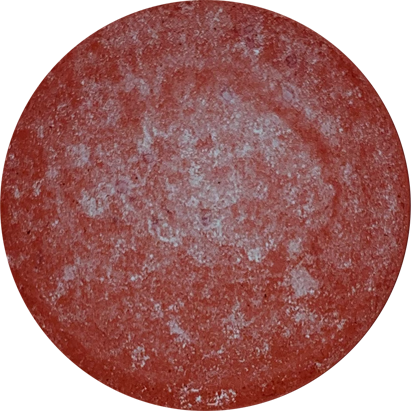

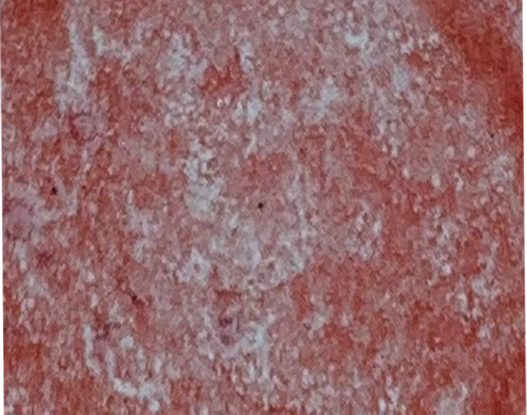


Fig5J-Zmat1^-/-^


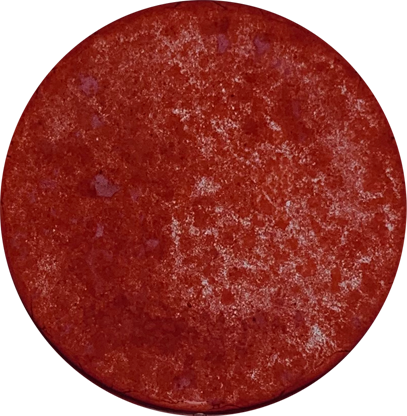

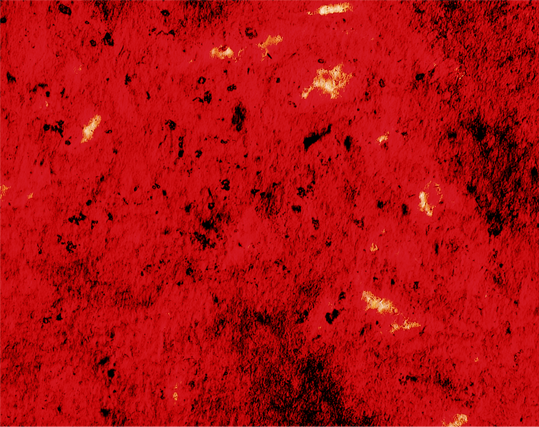


Fig6D


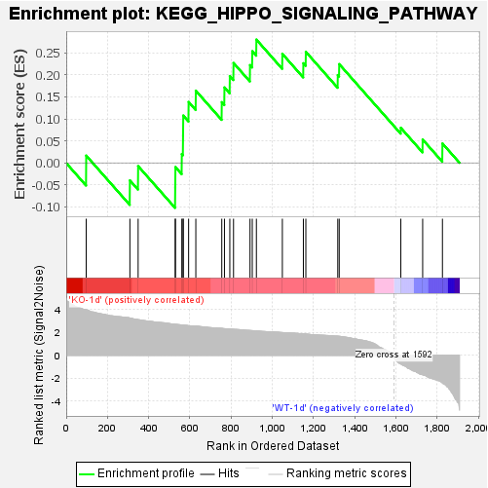


Fig6E


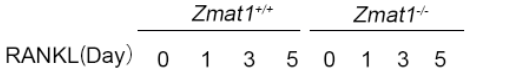


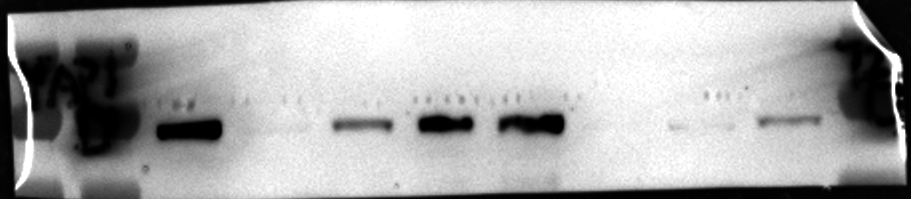


β-Actin(45kDa)

marker(70kDa)

YAP1(72kDa)


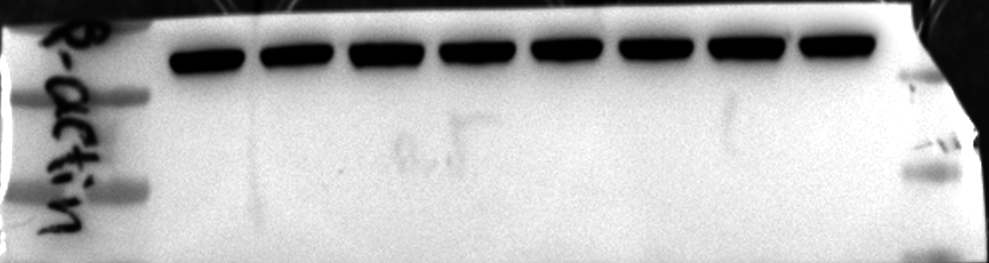


marker(40kDa)

Fig6G-Zmat1^+/+^-DMSO


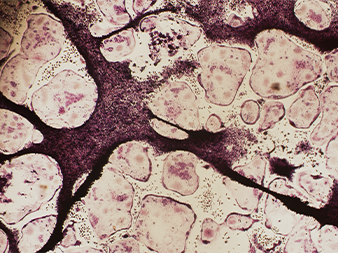


Fig6G-Zmat1^+/+^-SuperTDU


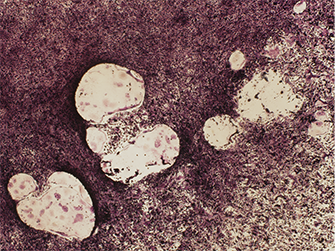


Fig6F-Zmat1^-/-^-DMSO


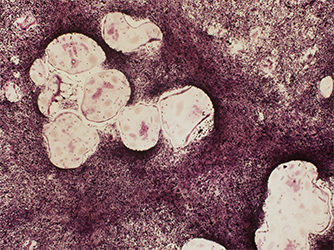


Fig6F-Zmat1^-/-^- SuperTDU


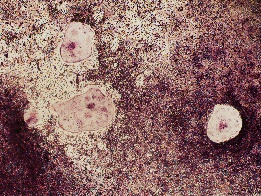


Fig6I


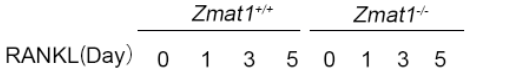


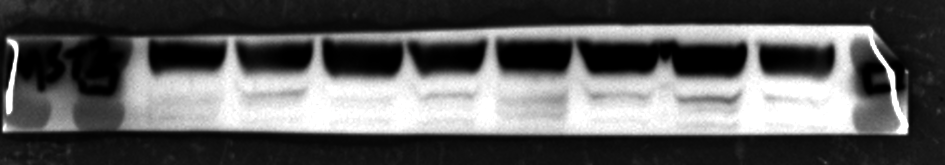


MST1(50kDa)


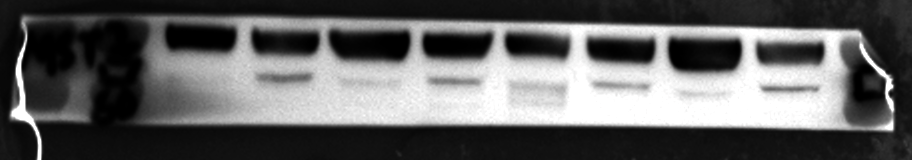


MST2(50kDa)


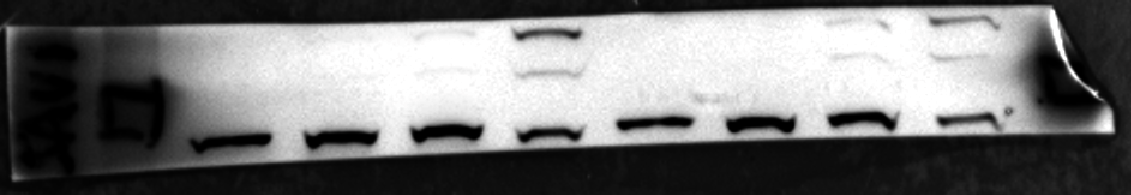


p-MST1(50kDa)


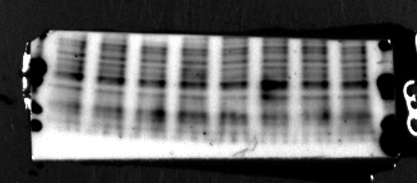


MOB1(20kDa)


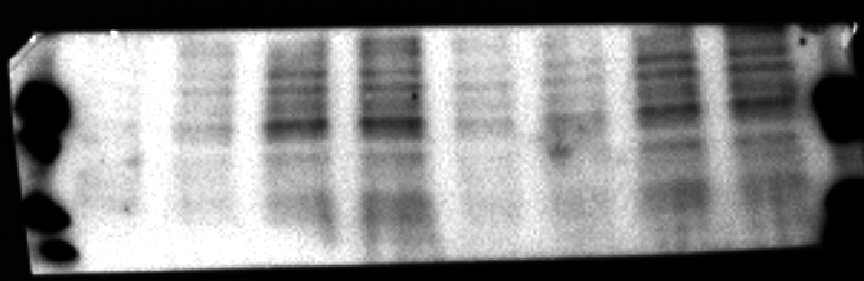


p-MOB1(20kDa)


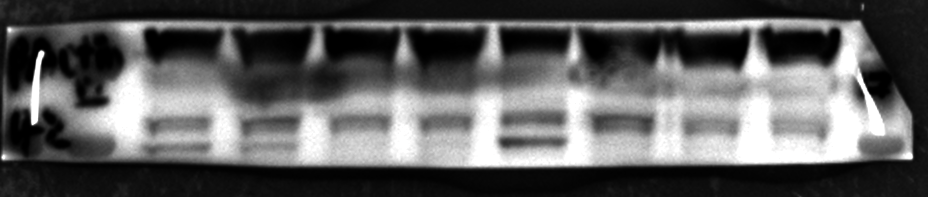


β-ACTIN(45kDa)

Fig8A -Zmat1^+/+^-siNC


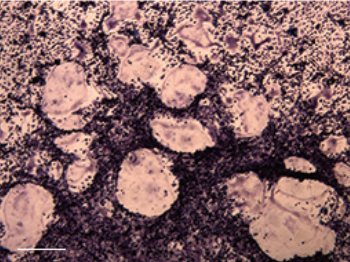


Fig8A -Zmat1^-/-^- siNC


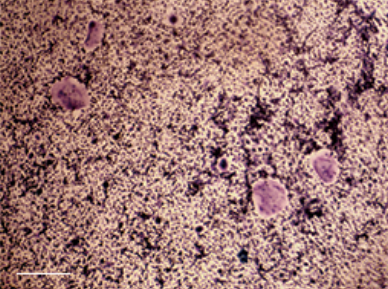


Fig8G -Zmat1^+/+^- siTrim46


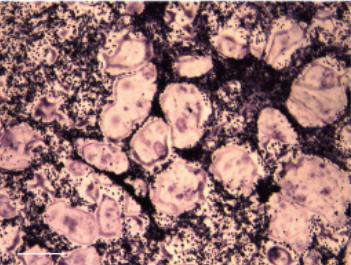


Fig8G -Zmat1^-/-^-siTrim46


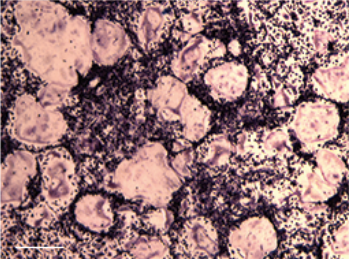


Fig8B

YAP1-V5

Input

TRIM46-MYC

IB:

YAP1-V5

β-Actin

TRIM46-MYC

IgG

MYC

IP:

YAP1-V5

TRIM46-MYC

marker(72kDa)

marker(72kDa)

marker(72kDa)

marker(72kDa)

marker(40kDa)

(72kDa)

(84kDa)

(72kDa)

(84kDa)

(42kDa)

Fig8C

YAP1-V5

Input

TRIM46-MYC

IB:

YAP1-V5

β-Actin

TRIM46-MYC

IgG

V5

IP:

TRIM46-MYC

YAP1-V5

marker(72kDa)

marker(95kDa)

marker(72kDa)

marker(72kDa)

marker(40kDa)

(72kDa)

(84kDa)

(72kDa)

(84kDa)

(42kDa)

Fig8D

70KDa

100KDa

IB:V5

70KDa

IB:MYC

40KDa

35KDa

IB:GAPDH

Fig8E

70KDa

IB:V5

100KDa

70KDa

IB:MYC

45KDa

40KDa

IB:GAPDH

Fig8G

MYC-blank + -

TRIM46-MYC - +

100KDa

100KDa

100KDa

150KDa

250KDa

35KDa

40KDa

70KDa

70KDa

70

70KDa

70KDa

70KDa

Input

IP:V5

HA

MYC

V5

GAPDH

MYC

V5

Mg132 + +

UB-K48-HA + +

YAP1-V5 + +

Fig8H

MYC-blank + -

35KDa

40KDa

70KDa

70KDa

100KDa

70KDa

100KDa

150KDa

70KDa

70KDa

100KDa

250KDa

UB-K63-HA + +

YAP1-V5 + +

TRIM46-MYC - +

Input

IP:V5

HA

MYC

V5

MYC

GAPDH

V5

Fig8I

250KDa

100KDa

150KDa

Ub-K48

70KDa

dIP YAP1

100KDa

TRIM46

70KDa

70KDa

YAP1

150KDa

250KDa

Ub-K48

70KDa

100KDa

100KDa

Input

70KDa

TRIM46

70KDa

YAP1

40KDa

35KDa

GAPDH

Fig8J

70KDa

YAP1

100KDa

70KDa

TRIM46

35KDa

40KDa

GAPDH

Fig8L

250KDa

100KDa

150KDa

Ub-K48

70KDa

70KDa

YAP1

100KDa

TRIM46

55KDa

70KDa

Fig8M

dIP-IB: HA

100KDa

250KDa

70KDa

1. dIP-IB: MYC& ② input-IB：MYC

70KDa

100KDa

1. dIP-IB: MYC& ② input-IB：MYC

70KDa

Input-IB：MYC

70KDa

Fig S1B-siNC

Fig S1B-siZmat1

Fig S1B-siCrip1

Fig S1B-siNr2f2

Fig S1B-siFhl1

Fig S1B-siNfib

Fig S1B-siNfia

Fig S1B-siEbf1

Fig S2B-siNC

Fig S2B-siZmat1

Fig S4A

Flag

（130KDa）

marker(100kDa)

β-Actin

（42KDa）

marker(40kDa)

FigS4B

NFATc1(95kDa)

marker(100kDa)

marker(40kDa)

β-Acitn(42kDa)

5

RANKL

Fig S4D-GFP

Fig S4D-Zmat1-Flag

Fig S4F-GFP-F-Actin

Fig S4F -GFP-DAPI

Fig S4F -GFP-Merge

Fig S4F -Zmat1-Flag-F-Actin

Fig S4F -Zmat1-Flag-DAPI

Fig S4F -Zmat1-Flag-Merge

Fig S4H -GFP

Fig S4H -Zmat1-Flag

Fig S5C -si-NC

Fig S5C -si-Zmat1

Fig S6C-Sham-Zmat1^+/+^→WT

Fig S6C-Sham-Zmat1^-/-^→WT

Fig S6C-OVX-Zmat1^+/+^→WT

Fig S6C-OVX-Zmat1^-/-^→WT

Fig S7C -si-NC-ALP

Fig S7C -si-Zmat1-ALP

Fig S7C -si-NC-ARS

Fig S7C -si-Zmat1-ARS

Fig S8A-Zmat1^+/+^-Sham

Fig S8A-Zmat1^+/+^-OVX

Fig S8A-Zmat1^-/-^-Sham

Fig S8A-Zmat1^-/-^-OVX

Fig S9A

YAP1(72kDa)

β-Acitn(42kDa)

Fig S9B

YAP1(72kDa)

β-Acitn(42kDa)

Fig S9C

YAP1(72kDa)

β-Actin(42kDa)

Fig S10A

SuperTDU

DMSO

NFATC1

100KDa

100KDa

MMP9

70KDa

CTSK

30KDa

40KDa

50KDa

β-Actin

Fig S11A-DMSO-ALP

Fig S11A-DMSO-ARS

Fig S11A-SuperTDU-ALP

Fig S11A-SuperTDU-ARS

Fig S11D

SuperTDU

DMSO

70KDa

50KDa

RUNX2

OPN

20KDa

50KDa

β-Actin

40KDa

Fig S11G-DMSO-Zmat1^+/+^

Fig S11G-SuperTDU-Zmat1^+/+^

Fig S11G-DMSO-Zmat1^-/-^

Fig S9L-SuperTDU-Zmat1^-/-^

Fig S11H-DMSO-Zmat1^+/+^

Fig S11H-SuperTDU-Zmat1^+/+^

Fig S11H-DMSO-Zmat1^-/-^

Fig S11H-SuperTDU-Zmat1^-/-^

Fig S12A-Control-Zmat1^+/+^

Fig S12A-OE-*Trim46*-Zmat1^+/+^

Fig S12A-Control-Zmat1^-/-^

Fig S12A-OE-*Trim46*-Zmat1^-/-^

Fig S12B-DMSO-siNC

Fig S12B-SuperTDU-siNC

Fig S12B-DMSO-siTrim46

Fig S12B-SuperTDU-siTrim46

Fig S12C-DMSO-Control

Fig S12C-SuperTDU-Control

Fig S12C-DMSO-OE-*Trim46*

Fig S12C-SuperTDU- OE-*Trim46*
